# *In silico* Study of Iron, Zinc and Copper Binding Proteins of *Pseudomonas syringae* pv. *lapsa*: Emphasis on Secreted Metalloproteins

**DOI:** 10.3389/fmicb.2018.01838

**Published:** 2018-08-21

**Authors:** Ankita Sharma, Dixit Sharma, Shailender K. Verma

**Affiliations:** Centre for Computational Biology and Bioinformatics, School of Life Sciences, Central University of Himachal Pradesh, Kangra, India

**Keywords:** *P. syringae* pv. *lapsa*, metal binding proteins, plant–pathogen interaction, secreted metalloproteins, *in silico*

## Abstract

The phytopathogenic bacteria, *Pseudomonas syringae* pv. *lapsa* (*P. syringae* pv. *lapsa*) infects the staple food crop wheat. Metalloproteins play important roles in plant-pathogen interactions. Hence, the present work is aimed to predict and analyze the iron (Fe), zinc (Zn), and copper (Cu) binding proteins of *P. syringae* pv. *lapsa* which help in its growth, adaptation, survival and pathogenicity. A total of 232 Fe, 307 Zn, and 38 Cu-binding proteins have been identified. The functional annotation, subcellular localization and gene ontology enriched network analysis revealed their role in wide range of biological activities of the phytopathogen. Among the identified metalloproteins, a total of 29 Fe-binding, 31 Zn-binding, and 5 Cu-binding proteins were found to be secreted in nature. These putative secreted metalloproteins may perform diverse cellular and biological functions ranging from transport, response to oxidative stress, proteolysis, antimicrobial resistance, metabolic processes, protein folding and DNA repair. The observations obtained here may provide initial information required to draft new schemes to control microbial infections of staple food crops and will further help in developing sustainable agriculture.

## Introduction

Transition metals are essential micronutrients for all living forms principally due to their ability to couple with proteins to form metalloproteins (Bowman et al., 2016). Approximately one-third of all the studied proteins require at least one metal ion to carry out its function (Shi and Chance, 2011). The interactions between protein and bound prosthetic metal atom in metalloproteins help them to complete the diverse functions of catalysis, regulation and structure stabilization (Andreini et al., 2013; Zhang et al., 2016).

The first-row transition metals such as iron (Fe), zinc (Zn), and copper (Cu) are vital for growth and proliferation of nearly all the organisms. Iron metal has incomplete “d” orbital which permits it to attain different oxidation states and redox chemistry (Cotton, 2000). As a result of this unique chemistry, iron act as cofactor for number of enzymes and has essential roles in oxygen metabolism, electron transport, tricarboxylic acid (TCA) cycle, lipid metabolism and peroxide detoxification (Krewulak and Vogel, 2008; Cornelis et al., 2011; Zhang, 2014; Ma et al., 2015). Zinc is one of the most abundant transition metal in the cell and plays important activity in structural stabilization and catalytic mechanisms of various proteins. Zinc containing proteins function in the broad range of cellular processes like storage, DNA replication, reactive oxygen species (ROS) detoxification, regulation of gene expression, carbohydrate oxidation and alcoholic fermentation (Murakami and Hirano, 2008; Maret, 2013; Ma et al., 2015). Copper is also an essential metal micronutrient for structural stabilization of proteins (Kaim and Rall, 1996) and for the catalysis of multiple redox activities of transport, denitrification and oxidative respiration (Cobine et al., 2006; Tavares et al., 2006; Festa and Thiele, 2012). Copper is also vital for various metabolic processes related to energy production, melanin biosynthesis and antioxidant defense (Kim et al., 2008).

Metals play key roles in various biochemical processes but are toxic in excess. It is also known that the concentration and availability of essential transition metals in the environment have significant roles in the plant-pathogen relationship. The competition between plant and phytopathogen for these essential micronutrients is a decisive factor for upshot, establishment and proliferation of infection (Fones and Preston, 2013). The phytopathogen invades the host cell and deregulates the host metal metabolism through nutrition alteration and genetic mutation which further raises the susceptibility for the infection (Fatima and Senthil-Kumar, 2015). In response, the host evolves the remedies that either block metal acquisition of the phytopathogen which restrict the availability of the nutrients or storm the phytopathogen with the higher concentration of metals (Hood and Skaar, 2012).

*Pseudomonas syringae (P. syringae)* is a Gram-negative, aerobic, polar flagella bearing rod-shaped phytopathogenic bacteria. *P. syringae* encompasses 64 pathovars, which could infect a number of agriculturally important plants such as tomato, wheat, barley, pea and apple (Young, 2010). *P. syringae* pv. *lapsa* is an obligate pathogen of wheat (*Triticum* spp.). The infection of *P. syringae* pv. *lapsa* results in poor grain quality, yield reduction and economic loss (Kong et al., 2016). *P. syringae* accounts for approximately 5–50% of wheat yield loss, worldwide (Valencia-Botín and Cisneros-Opez, 2012). Wheat is a staple food for majority of the global population and mineral micronutrients play important role in its growth, development and various biochemical processes (Verma et al., 2017). The numerous attempts have been made to biofortify it for mineral micronutrients, such as iron and zinc (Verma et al., 2016a,b). The wheat plant and its bacterial pathogen compete for these micronutrients (Sharma et al., 2017). A number of virulence determinants which contribute in the pathogenesis of phytobacteria are extracellular polysaccharides, flagella, secreted proteins, phytohormones, phytotoxin, multidrug efflux pumps and detoxification enzymes (Ichinose et al., 2013). The metal homeostasis has also been illustrated as a key virulence factor for the phytobacterial pathogenicity (Bronstein et al., 2005; Taguchi et al., 2010; Fones and Preston, 2013).

The scarcity and excess of the essential metals must be synchronized to attain metal equilibrium in an organism (Chandrangsu et al., 2017). To gain insight into the metal homeostatic mechanisms of an organism the preliminary requirement is to unveil the complete facts and knowledge of the metalloproteins encoded by those organisms. Several experimental techniques like immobilized metal affinity chromatography and mass spectrometry, inductively coupled plasma atomic emission spectrometer, metal-affinity columns and shift assay, nuclear magnetic resonance spectroscopy and high throughput X-ray absorption spectroscopy are used for the prediction of metalloproteins (Shi et al., 2011; Lu et al., 2012). The major analytical challenge which hampers the prediction of metalloproteins using these techniques is to preserve the native structure of the metalloproteins during analysis. To study metal-protein interactions, use of conventional techniques are also limited because these require denaturation of the proteins which may result in disruption of weak ionic interactions of the metalloprotein bonds (Lothian et al., 2013). Further, the experimental approaches are very costly, time-consuming, require specialized equipments and skilful handling during sample preparation which limit their use to analyze the whole metalloproteome of an organism (Lubec and Afjehi-Sadat, 2007). Recent advances in the whole genome sequencing and annotation technologies have provided the access to the whole genome and proteome of different organisms. A number of bioinformatics tools and databases are also available to explore whole proteome for identification and analysis of metalloprotein motifs (Mallick et al., 2011; Andreini et al., 2013). The literature study directs us to hypothesize that Fe, Zn, and Cu-binding proteins may have the significant impact on growth, development and virulence of *P. syringae* pv. *lapsa*. To understand role of metalloproteins and metal homeostasis mechanism of the selected phytopathogen, the primary requirement is to attain complete knowledge of all the Fe, Zn, and Cu-binding proteins. Therefore, this study was aimed at proteome wide identification of metalloproteins of wheat-phytopathogen *P. syringae* pv. *lapsa* ATCC 10859 using a systematic *in silico* approach, in which multiple computational programs have been used in combination to maintain the prediction accuracy. Additionally, due to the importance of transition metals in co-regulation of secreted metalloproteins, we have also identified the secreted metalloproteins which may be involved in the virulence of the bacteria. In this comprehensive *in silico* report, we are providing the list of putative candidate metalloproteins which may be effective start-up data for experimental analysis and could be targeted to reduce the virulence of the pathogenic bacteria. Furthermore, this will also enhance our knowledge regarding phytobacterial virulence and metal homeostasis.

## Materials and Methods

### Retrieval of Proteome Dataset and Identification of Iron, Zinc, and Copper Binding Sequence Motifs

We have retrieved the entire dataset of Fe, Zn, and Cu-binding proteins available at MetalPDB (Andreini et al., 2013). MetalPDB is a database which provides the detailed insight into the interactions of metal-binding biological macromolecules and focused on minimal functional site prediction of metalloproteins. MetalPDB database contains the data regarding features of metalloproteins from PDB, Pfam, CATH and SCOP databases. To search the proteome against MetalPDB database we have retrieved the entire dataset of Fe, Zn, and Cu-binding proteins available at MetalPDB. The whole proteome dataset of *P. syringae* pv. *lapsa* containing 4962 proteins, was retrieved from RefSeq National Center for Biotechnology Information (NCBI) ftp server^1^ (Kong et al., 2016). All of the 4962 proteins were searched against Fe, Zn, and Cu-binding proteins dataset of MetalPDB at expect value (*e*-value) 0.00001. The protein sequences which showed homology equal to and below the cut-off *e*-value were selected for further analysis.

### Identification of Iron, Zinc, and Copper Binding Structural Motifs and Analysis of Metal Interactions

The 3D structure modeling of selected proteins was carried out using Protein Homology/analogY Recognition Engine v 2.0 (Phyre2) computational program (Kelley et al., 2015). Phyre2 shape tertiary structure of proteins using HMM-HMM alignment based remote homology techniques. The high throughput modeled proteins above the threshold criteria of confidence ≥ 90% and query coverage ≥ 50% were selected for prediction of structural metal ion binding patterns. A fragment transformation method based server Metal Ion Binding site (MIB) (Lu et al., 2012) was used to scan the structural motifs for binding with metal ions Fe^2+^, Fe^3+^, Zn^2+^, Cu^+^, and Cu^2+^. In fragment transformation method the fragments of query proteins structurally align to the template of metal ion binding residues and alignment score was assigned to each residue of query protein on the basis of sequence and structure conservation evaluation, which is generated by BLOSUM62 substitution matrix and root mean square deviation of C-alpha carbons of structural local alignment. The predicted residues bind to metal ion are those which score above the assigned threshold alignment score. The overall accuracy of the MIB server is 94.6% with 60.5% true positive rate and 5% false positive rate. Ligplot^+^, (Laskowski and Swindells, 2011) a ligand-protein interaction visualization tool was used to visualize and analyze the protein-metal ions interactions. The protein structures carrying metal ions binding up to most effective interaction radii of 3.5Å were selected (Yamashita et al., 1990).

### Functional Annotation

The functional classification of the selected metalloproteins was done by analyzing conserved domains, family and superfamily using tools, InterProScan (Jones et al., 2014), Pfam (Finn et al., 2016) and National Center for Biotechnology Information -Conserved Domain Database (NCBI-CDD) (Marchler-Bauer et al., 2015) at default parameters. InterProScan database consists of integrated “signatures” or “predicted models” from diverse databases which represent the protein domain, functional sites and families. InterProScan provides robust, java based architecture which allow the biologist to analyze large genome scale protein-functional classification. The Pfam protein families database perform multiple sequence alignment to construct hidden markov model profile which is used to annotate protein families. CDD have experimentally derived site annotations which include active sites, protein-protein interaction sites, chemical binding and complement. In CDD the alignment models of representative sequence fragments are generated according to domain boundaries as found in 3D structure of the protein. Further, these alignments annotate the conserved features and model the structurally conserved cores of domain families. The putative Fe, Zn, and Cu-binding proteins were further classified broadly and clustered on the basis of illustration of identified functional domains for their probable biological roles using MEGA6 (Tamura et al., 2013) and BioEdit (Hall, 1999). The clustergram was visualized by EvolView (Zhang et al., 2012) program.

### Subcellular Localization

The subcellular localization of selected metalloproteins was done by CELLO (Yu et al., 2004), PSORTb v 3.0.2 (Yu et al., 2010) and Gneg-mPLoc (Shen and Chou, 2010). PSORTb program uses six modules each of having different module examining specific localization sites. By uniting the predictions from all the modules a Bayesian network is created which is used to predict the final localization of proteins on the basis of performance accuracies of each module. CELLO tool utilizes multilayered support vector machine classifiers to predict subcellular localization of Gram-negative bacteria. These multiple features vectors are based on multiple n-peptide compositions. Gneg-mPLoc server utilizes K-nearest neighbor algorithm to cluster the Swiss-Prot proteins with experimentally annotated subcellular localization on the basis of their amino acid properties and Gene Ontology (GO) terms for the prediction of subcellular localization of Gram-negative bacteria. The overall accuracy of CELLO, PSORTb and jackknife success rate of Gneg-mPLoc servers for prediction of subcellular localization of Gram-negative bacterial protein is 89, 98.3, and 85.5% respectively. In case of discrepancy between the results obtained from different servers, the consensus of results from two or more than two programs were considered for assigning precise subcellular locations.

### Building of Grouped GO Biological Functionally Annotated Network of Predicted Iron, Zinc, and Copper Binding Proteins

The GO biological function annotations (Ashburner et al., 2000) of all the predicted Fe, Zn, and Cu- binding proteins were done using ClueGO v2.3.3 (Bindea et al., 2009). ClueGO v2.3.3 is a Cytoscape v3.5.1 (Shannon et al., 2003) plug-in which was used to construct, visualize and analyze the GO enriched network. The GO biological process (BP) in the ClueGO program was represented by the node and the contacts between the GO BP terms on the basis of the overlap of their genes association, indicated by the edge. The kappa score (Huang et al., 2007) was used for the functional grouping of the GO terms and their enrichment significance was represented by the size of the nodes.

### Homology Search With Host Proteome and Identification of Secreted Proteins

The identified metalloproteins were searched against the host proteome *(Triticum aestivum*) using BLASTp (Basic Local Alignment Search Tool) (Altschul et al., 1990) at *e*-value 0.00001. The non-homologous proteins with *e*-value score more than 0.00001 were shortlisted. The selected proteins were further screened for Sec and Tat-dependent secretory pathways using SignalP 4.1 (Petersen et al., 2011) and TatP 1.0 server (Bendtsen et al., 2005) respectively. SignalP program is based on the combination of several artificial neural networks and predicts the signal peptide and its cleavage site with 87% accuracy (Bendtsen et al., 2004b). TatP server was used to screen Tat-motif, which is based on a combination of two artificial neural networks and predict the twin-arginine signal peptide and its cleavage site with 91% accuracy. The proteins with signal peptide were considered as Sec-dependent classical secreted proteins and proteins with Tat motif were considered as Tat-dependent secreted proteins. The proteins without signal peptides may secrete in an independent manner (non-classically secreted proteins) (Muesch et al., 1990) therefore, remaining proteins were examined by SecretomeP 2.0 (Bendtsen et al., 2004a) SecretomeP server uses artificial neural network architecture to predict non-classical secreted proteins which are based on sequence derived-protein features (amino acid composition, transmembrane helixes, gravy, protein disorder, secondary structure and instability index). The sensitivity of SecretomeP is 40% and is based on three-fold cross-validation and calculations of the correlation coefficient of the combinations of proteins features yielding best performance. The proteins with sec-score ≥ 0.5 were considered as non-classical secreted proteins. Further, the mature classically secreted proteins, Tat-dependent secreted proteins and non-classically secreted proteins were analyzed for the presence of hydrophobic regions (transmembrane alpha helix). This is because hydrophobic regions act as stop transfer signal and anchor the proteins in the inner-membrane. The transmembrane alpha helix prediction was done by HMM-based Transmembrane Hidden Markov Model (TMHMM v 2.0) (Krogh et al., 2001) and Hidden Markov Model for topology prediction (HMMTOP) (Tusnady and Simon, 2001) having accuracy is 79 and 78% respectively. These shortlisted proteins also checked for their subcellular localization which is described in the previous section. The proteins without membrane anchor transmembrane helix and found to be localized either in periplasm, outermembrane or extracellular region were selected. These selected proteins which are either secreted by Sec-dependent, Tat-dependent and non-classical secretory pathways, having absence of transmembrane helix and localized either to periplasm, outer-membrane or extracellular space were categorized as secreted metalloproteins.

### Molecular Docking of Secreted Metalloproteins

The molecular metal ion docking in selected secreted metalloproteins was done manually on the basis of the template from MetalPDB by replacement of the coordinates into the proteins using PyMol (DeLano, 2002). The structures of the template and selected protein were aligned and coordinates of metal ion and ligand were saved. Coot 0.7.2 (Emsley and Cowtan, 2004) was used to merge both the pdb files. The docked proteins were visualized by PyMol for studying the interactions between metal ion(s) and amino acid residues.

## Results

**FIGURE 1:**
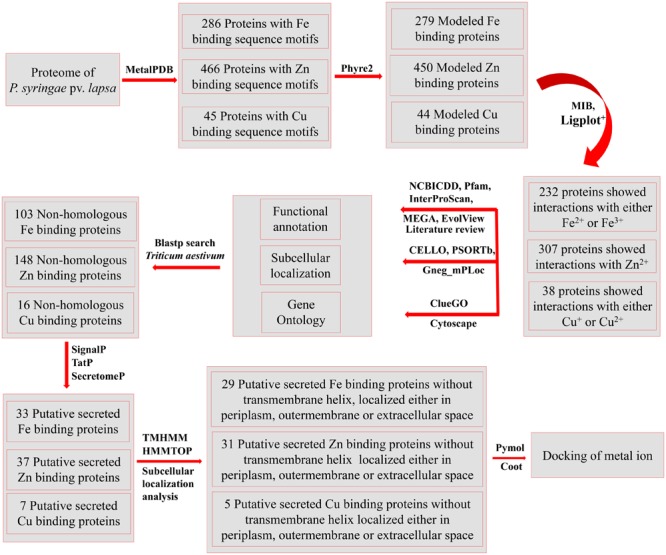
The overall methodology of the present study. The whole proteome of *P. syringae* pv. *lapsa* was analyzed for identification of metal (Fe, Zn, and Cu) binding sequence motifs using dataset of MetalPDB (Andreini et al., 2013) at *e*-value of 0.00001. The high throughout modeling of selected proteins was done by Phyre2 (Kelley et al., 2015) and selected models were analyzed for metal ion binding structural motifs using MIB (Lu et al., 2012) and Ligplot^+^ (Laskowski and Swindells, 2011). The functional annotation was done by InterProScan (Jones et al., 2014), Pfam (Finn et al., 2016), and NCBI-CDD (Marchler-Bauer et al., 2015). Clustering of the proteins were done by MEGA6 (Tamura et al., 2013) and EvolView (Zhang et al., 2012). The consensus of bioinformatics server CELLO (Yu et al., 2004), PSORTb (Yu et al., 2010), and Gneg-mPLoc (Shen and Chou, 2010) was used for subcellular localization of predicted metalloproteins. The Gene Ontology based biological function annotation networks were constructed by ClueGO (Bindea et al., 2009) and visualized by Cytoscape (Shannon et al., 2003). Blastp (Altschul et al., 1990) search was done against host proteome (*Triticum aestivum*) to identify non-homologous Fe, Zn, and Cu-binding proteins. Further, the putative secreted metal binding proteins were predicted by using SignalP (Petersen et al., 2011) TatP (Bendtsen et al., 2005) and SecretomeP (Bendtsen et al., 2004a) databases. The transmembrane analysis was done by TMHMM (Krogh et al., 2001) and HMMTOP (Tusnady and Simon, 2001) web servers. PyMol (DeLano, 2002) and Coot (Emsley and Cowtan, 2004) server were used for manual docking of metal ions and visualization of interactions.

**FIGURE 2:**
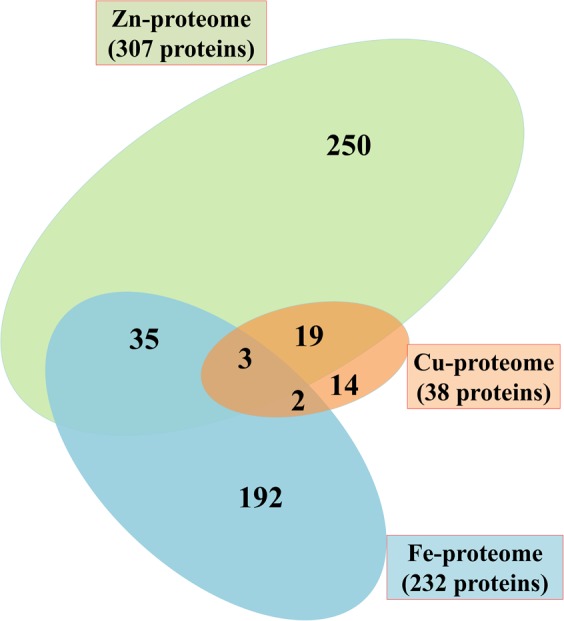
Venn diagram showing Fe, Zn, and Cu-binding proteome of *P. syringae* pv. *lapsa.* A total of 232, 307 and 38 Fe, Zn, and Cu binding proteins have been identified, respectively. Out of these, 35 proteins have Fe/Zn, 19 proteins have Zn/Cu and 2 proteins have Fe/Cu binding sites. Three proteins could bind with Fe, Zn, and Cu ions.

The proteome scale investigation of *P. syringae* pv. *lapsa* for identification of Fe, Zn, and Cu-binding proteins was carried out using the systematic bioinformatics approach (**Figure 1**). It was revealed that most of the proteins were Zn-binding (307) followed by Fe (232) and Cu-binding (38) proteins. Zinc is known to be the most abundant metal ion in the living world and hence, the higher percentage of Zn was expected. Some proteins have interactions with more than two metal ions on the same or different active site. The grouping of Fe/Zn was the most recurrent combination subsequently followed by Zn/Cu and Fe/Cu (**Figure 2**), respectively.

### Iron Binding Proteome of *P. syringae* pv. *lapsa*

**FIGURE 3:**
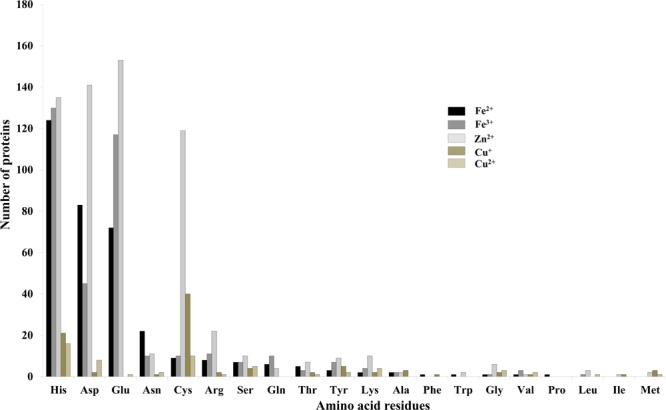
Interactions of amino acid residues and metal ions. Bar diagram showing the interaction of amino acid residues with metal ions (Fe^2+^, Fe^3+^, Zn^2+^, Cu^+^, and Cu^2+^). The Y-axis showing number of proteins and X-axis showing name of amino acid residues. Gln, Asp, His, Cys, Arg, and Asn were the most common interacting residues with different metal ions.

**FIGURE 4:**
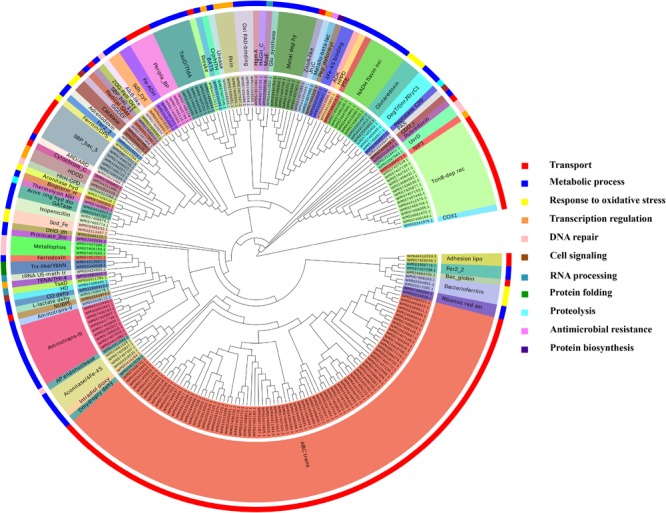
Functional classification of Fe-binding proteins. The clustergram was generated by MEGA6 program (Tamura et al., 2013). The inner circle represents the sequence identifiers. The middle circle shows the functional domains/family of the respective sequence identifier. The color code given here is to represent only the outer circle which denote the broad classification of Fe-binding proteins on the basis of their biological roles. The most common domains were ABC transporters, TBDRs, SBPs, aminotransferase-III and NADH falvin-oxidoreductase. These proteins have diverse roles mainly in transport, metabolic processes and response to oxidative stress.

**FIGURE 5:**
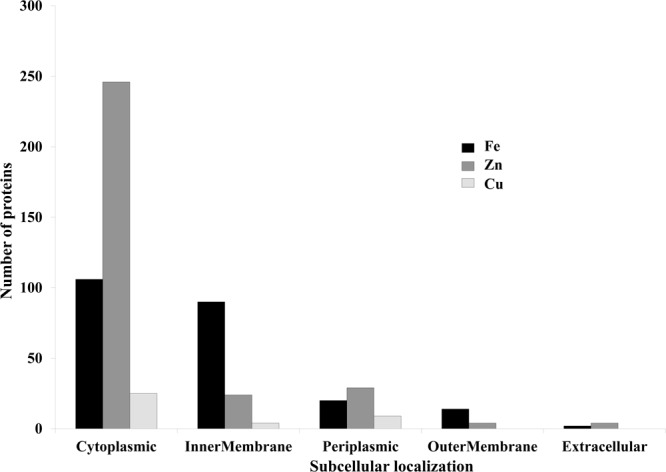
Subcelluar-localization of predicted Fe, Zn, and Cu-binding proteins. This graph is showing subcellular localizations of the predicted metalloproteins. *X*-axis represents localization and *Y*-axis showing number of proteins. Most of the proteins were found to be localized in cytoplasm and inner-membrane.

**FIGURE 6:**
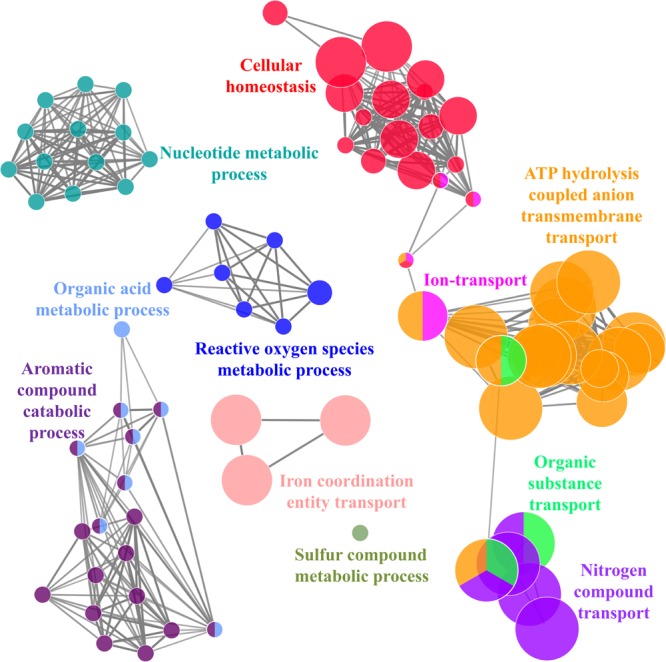
The ClueGO functionally grouped annotation network of Fe-binding proteins constructed at kappa score ≥ 0.4 showing 7 significant groups of GO terms (ATP hydrolysis coupled anion transmembrane transport, organic substance transport, nitrogen compound transport, ion transport, iron coordination entity transport, cellular homeostasis and ROSs metabolic process). Each circle represent particular BP node. Colors of node represent the group they belong. The node belongs to multiple groups shown by mixed coloring.

It was identified that out of 4962 proteins of *P. syringae* pv. *lapsa*, a total of 286 have Fe-binding sequence motifs. Out of these, only 279 proteins were modeled by Phyre2 on the basis of homology. Using MIB and Ligplot^+^ tools, it was identified that 232 proteins have sequence and structural motifs for either Fe^2+^or Fe^3+^ ions. These identified Fe-binding proteins constitute about 4.7% of the total proteome. His, Glu, Asp, Asn, Arg, and Cys were the common interacting residues in the binding pocket of Fe-binding proteins. The order of interactions of protein and Fe ions are shown in **Figure 3**. The domain analysis of Fe-binding proteins showed the abundance of ABC transporter, TonB dependent receptors (TBDRs), aminotransferase, solute binding proteins (SBPs) and NADH flavin oxidoreductase/NADH oxidase (Nox) domains. On the basis of literature review of the identified domains Fe-binding proteins were further categorized in to 11 broad classes namely transport (116), metabolic process (75), response to oxidative stress (12), transcription regulation (9), DNA repair (8), cell signaling (3), RNA processing (3), protein folding (2), proteolysis (2), antimicrobial resistance (1), and protein biosynthesis (1). The detailed functional domain analysis is shown in **Figure 4** and **Supplementary Table S1**. The subcellular localization of Fe-binding proteins was in accordance with their functional categories. The analysis revealed that most of the proteins involved in metabolic processes and response to oxidative stress were localized in the cytoplasm (106) while many of the transporter proteins were confined to inner-membrane (90), periplasmic space (20) and outer-membrane (14). Two proteins were found in extracellular space (**Figure 5** and **Supplementary Table S1**). The enriched GO BP network of the identified Fe-binding proteins was configured on 11 kappa score groups with 83 BP nodes and 278 edges. A total of 7 significant groups of GO terms (ATP hydrolysis coupled anion transmembrane transport, organic substance transport, nitrogen compound transport, ion transport, iron coordination entity transport, cellular-homeostasis and ROSs metabolic process) were identified (**Figure 6**). The analysis of the network was done to determine the importance of Fe-binding protein-encoding genes in the regulation of BPs by the estimation of a number of interactions in which involvement of each single node was noted. The most connected GO terms were allied with transport (GO:0006810) having 66 links and transmembrane transport (GO:0055085) with 43 links (**Supplementary Table S2**). Therefore, we have found that GO BP network of Fe-binding proteins satisfies the results of domain-based functional classification. The homology search of 232 Fe-binding proteins with host proteome (*Triticum aestivum*) revealed that 103 proteins were non-homologous to bread wheat. Out of these 103 non-homologous proteins, 29 (19 Sec-dependent, 1 Tat-dependent and 9 non-classical) proteins were found to be putative secreted in nature without any transmembrane helix (**Supplementary Table S3**). These proteins were found to be localized in periplasm, outer-membrane and extracellular space and were functionally involved in transport, response to oxidative stress, proteolysis and metabolic processes (**Table 1**). The manual docking of the putative secreted Fe-binding proteins based on the template from MetalPDB database search showed that proteins have similar binding pockets as the template. This structural protein-Fe ion interaction analysis helps in understanding the structure based functional behavior of the proteins. The detailed summary has been given in **Supplementary Figure S1** and **Supplementary Table S4**.

**Table 1:** Subcellular localization and functional domain analysis of secreted iron, zinc, and copper binding proteins.

S. No.	Sequence Id	Metal	Subcellular localization	Functional domain/family	Description	Reference
1	WP_024661472.1	Fe	OuterMembrane	TonB-dependent receptor, plug domain	Transport	Taguchi et al., 2010
2	WP_024684592.1	Fe	OuterMembrane	TonB-dependent receptor, plug domain	Transport	Taguchi et al., 2010
3	WP_044312375.1	Fe	OuterMembrane	TonB-dependent receptor, plug domain	Transport	Taguchi et al., 2010
4	WP_057405877.1	Fe	OuterMembrane	TonB-dependent receptor, plug domain	Transport	Taguchi et al., 2010
5	WP_057406095.1	Fe	OuterMembrane	TonB-dependent receptor, plug domain	Transport	Taguchi et al., 2010
6	WP_057406164.1	Fe	OuterMembrane	TonB-dependent receptor, plug domain	Transport	Taguchi et al., 2010
7	WP_057406756.1	Fe	OuterMembrane	TonB-dependent receptor, plug domain	Transport	Taguchi et al., 2010
8	WP_057407139.1	Fe	OuterMembrane	TonB-dependent receptor, plug domain	Transport	Taguchi et al., 2010
9	WP_057406030.1	Fe	OuterMembrane	TonB-dependent receptor, plug domain	Transport	Taguchi et al., 2010
10	WP_057406431.1	Fe	OuterMembrane	TonB-dependent receptor, plug domain	Transport	Taguchi et al., 2010
11	WP_057407153.1	Fe	OuterMembrane	TonB-dependent receptor, plug domain	Transport	Taguchi et al., 2010
12	WP_057407617.1	Fe	OuterMembrane	TonB-dependent receptor, plug domain	Transport	Taguchi et al., 2010
13	WP_057407519.1	Fe	OuterMembrane	TonB-dependent receptor, plug domain	Transport	Taguchi et al., 2010
14	WP_057407445.1	Fe	OuterMembrane	TonB-dependent receptor, plug domain	Transport	Taguchi et al., 2010
15	WP_003317349.1	Zn	Periplasmic	Bacterial extracellular solute-binding proteins, family 3	Transport	Handali et al., 2015
16	WP_003424807.1	Zn	Periplasmic	Bacterial extracellular solute-binding proteins, family 3	Transport	Handali et al., 2015
17	WP_003428827.1	Zn	Periplasmic	Bacterial extracellular solute-binding proteins, family 3	Transport	Handali et al., 2015
18	WP_044313164.1	Zn	Periplasmic	Bacterial extracellular solute-binding proteins, family 3	Transport	Handali et al., 2015
19	WP_057406454.1	Zn	Periplasmic	Bacterial extracellular solute-binding proteins, family 3	Transport	Handali et al., 2015
20	WP_057406536.1	Zn	Periplasmic	Bacterial extracellular solute-binding proteins, family 3	Transport	Handali et al., 2015
21	WP_003318111.1	Zn	Periplasmic	Bacterial extracellular solute-binding proteins, family 5	Transport	Handali et al., 2015
22	WP_057406766.1	Zn	Periplasmic	Bacterial extracellular solute-binding proteins, family 5	Transport	Handali et al., 2015
23	WP_057406898.1	Zn	Periplasmic	Bacterial extracellular solute-binding proteins, family 5	Transport	Handali et al., 2015
24	WP_057406924.1	Zn	Periplasmic	Bacterial extracellular solute-binding proteins, family 5	Transport	Handali et al., 2015
25	WP_057407520.1	Zn	Periplasmic	Bacterial extracellular solute-binding proteins, family 5	Transport	Handali et al., 2015
26	WP_032662671.1	Fe	Periplasmic	Bacterial extracellular solute-binding proteins, family 5	Transport	Tai et al., 2003
27	WP_057406235.1	Fe	Periplasmic	Bacterial extracellular solute-binding proteins, family 5	Transport	Tai et al., 2003
28	WP_057405715.1	Zn, Fe	Periplasmic	Bacterial extracellular solute-binding proteins, family 5	Transport	Tai et al., 2003; Handali et al., 2015
29	WP_057406194.1	Zn, Fe	Periplasmic	Bacterial extracellular solute-binding proteins, family 5	Transport	Tai et al., 2003; Handali et al., 2015
30	WP_057406522.1	Zn, Fe	Periplasmic	Bacterial extracellular solute-binding proteins, family 5	Transport	Tai et al., 2003; Handali et al., 2015
31	WP_058877211.1	Zn, Fe	Periplasmic	Bacterial extracellular solute-binding proteins, family 5	Transport	Tai et al., 2003; Handali et al., 2015
32	WP_057406514.1	Fe, Cu	Periplasmic	Bacterial extracellular solute-binding protein	Transport	Tam and Saier, 1993; Tai et al., 2003
33	WP_057405766.1	Fe	Periplasmic	Periplasmic binding protein	Transport	Shouldice et al., 2004; Cangelosi et al., 1990
34	WP_057405965.1	Fe	Periplasmic	Periplasmic binding protein	Transport	Shouldice et al., 2004; Cangelosi et al., 1990
35	WP_057406118.1	Fe	Periplasmic	Periplasmic binding protein	Transport	Shouldice et al., 2004; Cangelosi et al., 1990
36	WP_003346032.1	Zn	Periplasmic	Periplasmic binding protein	Transport	Chandra et al., 2007
37	WP_003425596.1	Zn	Periplasmic	Periplasmic binding protein	Transport	Chandra et al., 2007
38	WP_044312913.1	Fe	Periplasmic	Adhesion lipoprotein	Transport	Berne et al., 2015; Ghazaei, 2016
39	WP_057406036.1	Fe	Periplasmic	Adhesion lipoprotein	Transport	Berne et al., 2015; Ghazaei, 2016
40	WP_057406538.1	Fe	Extracellular	Aconitase family	Response to oxidative stress	Kirchberg et al., 2012
41	WP_057407465.1	Zn	Periplasmic	DJ-1/PfpI family	Response to oxidative stress	Bankapalli et al., 2015
42	WP_057406401.1	Zn, Cu	Periplasmic	Copper/zinc superoxide dismutase (SODC)	Response to oxidative stress	Fones and Preston, 2012
43	WP_057407566.1	Zn, Cu	Periplasmic	Azurin family	Response to oxidative stress	Swingle et al., 2008
44	WP_057406961.1	Fe, Zn	Extracellular	Thermolysin metallopeptidase, alpha-helical domain/Peptidase M4	Proteolysis	Kyostio et al., 1991
45	WP_003411605.1	Zn	Extracellular	Peptidase M10 serralysin	Proteolysis	Zhang et al., 1999
46	WP_057406970.1	Zn	Extracellular	Peptidase M10 serralysin	Proteolysis	Zhang et al., 1999
47	WP_044312611.1	Zn, Cu	Periplasmic	Trypsin	Proteolysis	Tripathi and Sowdhamini, 2008
48	WP_057407431.1	Zn	Periplasmic	Beta-lactamase	Antimicrobial resistance	Bebrone, 2007
49	WP_003316096.1	Zn	OuterMembrane	Peptidase family M23	Antimicrobial resistance	Grabowska et al., 2015
50	WP_003422785.1	Zn	OuterMembrane	Peptidase family M23	Antimicrobial resistance	Grabowska et al., 2015
51	WP_057405551.1	Zn	OuterMembrane	Peptidase family M23	Antimicrobial resistance	Grabowska et al., 2015
52	WP_057406372.1	Zn	OuterMembrane	Peptidase family M23	Antimicrobial resistance	Grabowska et al., 2015
53	WP_003416184.1	Fe	Periplasmic	Intradiol ring-cleavage dioxygenase	Metabolic process	Bianchetti et al., 2013
54	WP_016567277.1	Zn	Periplasmic	Isochorismatase, RutB	Metabolic process	Liu et al., 2014
55	WP_003317093.1	Zn	Periplasmic	Single-strand binding protein family	DNA repair	Citovsky et al., 1988
56	WP_003424002.1	Cu	Periplasmic	Copper chaperone SCO1/SenC	Protein folding	Seib et al., 2003; Lohmeyer et al., 2012

### Zinc Binding Proteome of *P. syringae* pv. *lapsa*

**FIGURE 7:**
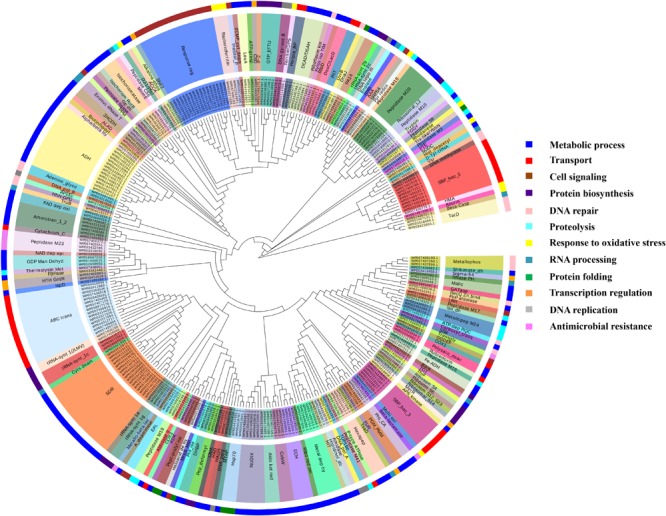
Functional classification of identified Zn-binding proteins. The clustergram of Zn-binding proteins clustered by Mega (Tamura et al., 2013). The inner circle denotes the sequence identifiers. The middle circle represents the functional domains/family of the respective sequence identifier. The outer circle shows the broad classification of Zn-binding proteins on the basis of their biological roles. The color code given here represents the outer circle only. The Zn-binding proteins predominantly showed the presence of SDR, ADH/GroES, ABC transpoters, response regulators and SBPs domains. Most of the Zn-binding proteins were play significant roles in metabolic processes, transport, cell signaling, protein biosynthesis and protein folding.

**FIGURE 8:**
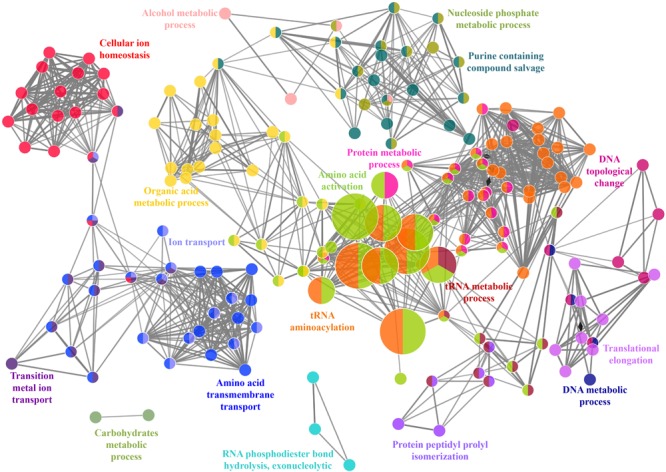
Functionally annotated biological process network for Zn-binding proteins constructed by ClueGO at kappa score ≥ 0.4 represents 4 significant groups of GO terms (amino acid activation, tRNA aminoacylation, protein metabolic process and tRNA metabolic process). Each circle represent particular BP node. The node color represents the group they belong and mixed coloring of the node represents the node belongs to multiple groups.

A total of 466 proteins were predicted to have Zn-binding sequence motifs from the whole proteome of *P. syringae* pv. *lapsa*. Out of them, 450 proteins were modeled by Phyre2 and 307 showed the presence of Zn-binding sequence and structural patterns. The common interacting residues with Zn^2+^ ion was Glu, Asp, His and Cys (**Figure 3**). Short-chain dehydrogenase/reductase (SDR), response regulator receiver, alcohol dehydrogenase/GroES (ADH/GroES), ABC transporter and SBPs were major domains among Zn-binding proteins. Based on the literature survey we have functionally classified Zn-binding proteins into 12 different classes viz. metabolic process (135), transport (39), cell signaling (24), protein biosynthesis (23), DNA repair (17), proteolysis (17), response to oxidative stress (11), RNA processing (11), protein folding (8), transcription regulation (8), DNA replication (7) and antimicrobial resistance (7) (**Figure 7** and **Supplementary Table S5**). The subcellular localization of Zn-binding proteins showed that 246 proteins were confined to the cytoplasm. Most of these cytoplasmic proteins belong to the category of metabolic process, protein biosynthesis, transcription regulation, DNA replication, DNA repair, protein folding and RNA processing. Twenty-nine proteins localized to the periplasm and 24 were confined to inner-membrane, many of them related to transport. Four outer-membrane and 4 extracellular proteins have also been identified which belong to the category of antimicrobial resistance and proteolysis. (**Figure 5** and **Supplementary Table S5**). The GO BP functionally annotated network of Zn-binding proteins was constructed on 18 kappa score groups by ClueGO having 174 nodes and 679 edges. The network analysis showed 4 significant groups of GO terms (amino acid activation, tRNA aminoacylation, protein metabolic process and tRNA metabolic process) (**Figure 8**). The BP nodes macromolecule metabolic process (GO:0043170) and cellular nitrogen compound metabolic process (GO:0034641) were most connected GO terms with 91 and 90 links respectively (**Supplementary Table S6**). Additionally, some BP nodes showed alliance with more than two functional groups which signify possible involvement of their associated genes in the regulation of multiple BPs (**Supplementary Table S6**). A total of 148 non-homologous Zn-binding proteins were further selected after homology search with host proteome. Out of these 148 proteins, 31 (19 Sec-dependent, 1 Tat-dependent and 11 non-classical) were identified as putative Zn-binding secreted proteins having no transmembrane helix (**Supplementary Table S7**). Subcellularly these proteins confined to periplasm, outer-membrane and extracellular space (**Table 1**). The putative Zn-binding secreted proteins were involved in transport, antimicrobial resistance, proteolysis, response to oxidative stress, metabolic process and DNA repair (**Table 1**). The Zn ion was manually docked in these identified secreted proteins on the basis of template obtained from MetalPDB which showed similar binding amino acid residues. The representative tertiary structure of each domain of putative secreted Zn-binding proteins was shown in **Supplementary Figure S2** and **Supplementary Table S8**.

### Copper Binding Proteome of *P. syringae* pv. *lapsa*

**FIGURE 9:**
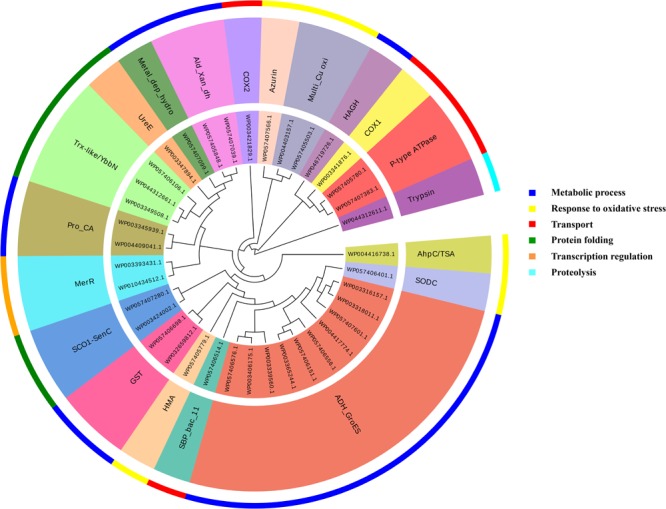
Functional classification of identified Cu-binding proteins. Cu-binding proteins enlisted in the form of clustergram where the inner circle denotes the sequence identifiers. The middle circle represents the functional domains/family of the respective sequence identifier. The outer circle denotes the broad classification of Cu-binding proteins on the basis of their biological roles and color code here represents the outer circle only. Most of the Cu-binding proteins found to carry out the function of metabolism having ADH/GroES domain and protein which function in transport have P-type ATPase, COX and SBP domains.

**FIGURE 10:**
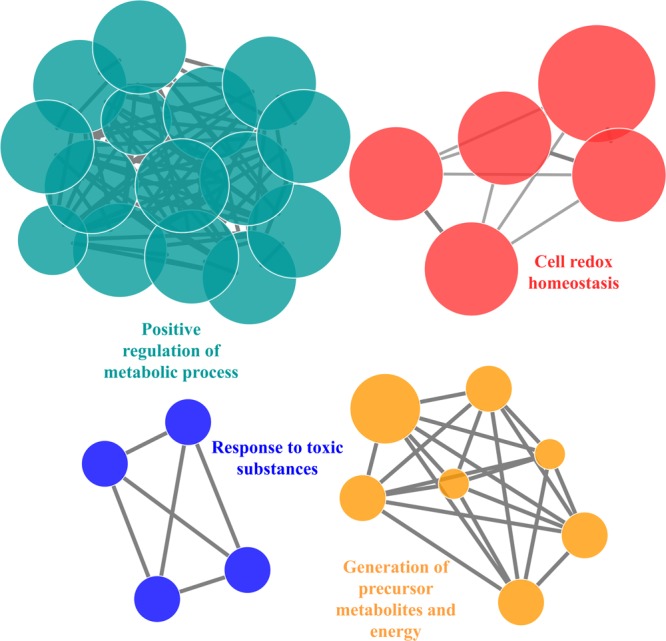
The functionally biological process annotated network for Cu-binding proteins constructed by ClueGO at kappa score ≥ 0.4 represents 4 significant groups of GO terms (positive regulation of metabolic process, cell redox homeostasis, generation of metabolites and energy, response to oxidative stress). Each circle represent particular BP node and the node color represent the group they belong.

We have found 45 proteins with Cu-binding sequence motifs from the whole proteome of *P. syringae* pv. *lapsa*. Out of these, 44 proteins were modeled by Phyre2. Thirty-eight Cu-binding proteins showed sequence and structural motifs either for Cu^+^ or for Cu^2+^. Cys, His, Tyr, Ser and Met were common interacting residues with Cu^+^ ion and His, Cys and Asp were common interacting residues with Cu^2+^ ion (**Figure 3**). The functional domain analysis of identified Cu-binding proteins indicates the major presence of ADH/GroES, Trx-like/YbbN, multiple Cu-oxidase and P-type ATPase domains. The literature studies of the domains present in these Cu-binding proteins help us to categorize them into 6 broad classes which include metabolic process (18), response to oxidative stress (6), protein folding (6), transport (5), transcription regulation (2) and proteolysis (1) (**Figure 9** and **Supplementary Table S9**). Twenty-five Cu-binding proteins found to be localized in the cytoplasm, which were generally involved in process of metabolism. Nine proteins were found in periplasm, most of them related to the category of response to oxidative stress. Four proteins localized to inner-membrane belong to transport category. (**Figure 5** and **Supplementary Table S9**). The ClueGO functionally grouped annotation BP network of Cu-binding proteins showed 31 nodes and 87 edges at 4 kappa score groups (**Figure 10**). Four significant groups have BP nodes for cell redox homeostasis, positive regulation of metabolic process, generation of precursor metabolites and energy and response to toxic substances. The nodes representing positive regulation of metabolic process (GO:0009893) was the most connected GO terms with 21 links (**Supplementary Table S10**). Further, 16 Cu-binding proteins found to be non-homologous to host proteome and out of them 5 (4 Sec-dependent and 1 Tat-dependent) were found to be putative secreted in nature and none of them has transmembrane helix (**Supplementary Table S11**). All the 5-putative secreted Cu-binding proteins were localized to the periplasm and functions in response to oxidative stress, transport, protein folding and proteolysis (**Table 1**). After manual docking on the basis of the template obtained from MetalPDB, we have found similar binding patterns for the putative secreted Cu-binding proteins as that of the template (**Supplementary Figure S3** and **Supplementary Table S12**).

## Discussion

The proportion of metalloproteins in the entire proteome varies from prokaryotes to eukaryotes (Dupont et al., 2010). The presented study based on the bioinformatics analysis of Fe, Zn, and Cu-binding proteins of *P. syringae* pv. *lapsa* for their probable role in bacterial physiology and virulence. This study signifies the prediction of both sequence and structural metalloproteins motifs on the basis of datasets of MetalPDB (Andreini et al., 2013). A total of 577 metallproteins has been identified from *P. syringae* pv. *lapsa* which includes 232 Fe, 307 Zn and 38 Cu-binding proteins. The presence of metalloproteins obtained from *P. syringae* pv. *lapsa* proteome has conformity with earlier reports for Fe, Zn, and Cu proteomes of bacteria (Andreini et al., 2006, 2007, 2008). It was earlier reported that some proteins are specific to particular ligand while some proteins are highly promiscuous and may interact with one or more metal ions (Schreiber and Keating, 2011). This characteristic of protein is dependent on its structural properties such as conformational plasticity and amino acid residues at the catalytic site. Thirty-five Fe-binding proteins and 19 Cu-binding proteins share Zn-binding motifs. Two Cu-binding proteins share motifs with Fe-binding proteins and 3 proteins have motifs for 3 of the metal ions, i.e., Fe, Zn and Cu. All these are transition metals (Fe, Zn, and Cu) and therefore have a tendency to interact with amino acid residues of similar chemical nature. The most common interacting residues reported in the binding site of Fe, Zn, and Cu-binding proteins were His, Asp, Glu, and Cys which were in accordance with previous reports that polar or charged amino acid involved in coordination of metal ion (Auld, 2001; Babor et al., 2008).

The functional domain analysis of Fe-binding proteins showed their major role in transport, metabolism and response to oxidative stress. The transporter Fe-binding proteins were enriched in the inner-membrane ABC transporter, outer-membrane TBDRs and periplasmic SBPs domains which may act as receptors for the transport of Fe ions and Fe bound complexes for maintenance of Fe-homeostasis (Taguchi et al., 2010; Tai et al., 2003; Tanaka et al., 2017). The Fe-binding proteins involved in different metabolic processes were mostly localized in the cytoplasm and were predominantly contain aminotransferase-III, Nox, metal dependent hydrolase and TauD/TfdA domains. The aminotransferase III have transaminase and pyridoxal phosphate binding activity which helps in catalyzing various biosynthetic processes (Marienhagen et al., 2005). The FMN binding and oxidoreductase activity of Nox domain also help to regulate various metabolic processes (Yamamoto et al., 2006). The metal-dependent hydrolases involved in the hydrolysis of the wide spectrum of substrates (Hernick and Fierke, 2013) and TauD/TfdA domain-containing proteins have oxidation-reduction activity to catalyze the discharge of sulfite from taurine during sulfur starvation (Elkins et al., 2002). The Fe-binding proteins belonging to the category of response to oxidative stress mostly contain bacterioferritin, catalase and iron/manganese superoxide dismutase (Fe/Mn SOD) domains. Bacterioferritin (iron storage proteins) have ferroxidase activity which assists detoxification and protection from free radicals (Andrews, 1998). The catalase and Fe/Mn SOD catalyzes the conversion of free radicals and super-radicals to molecular oxygen which helps in regulating cellular redox homeostasis (Fones and Preston, 2012). The GO BP network analysis of Fe-binding proteins supports their functional domain classification, as the network also indicate their major role in transport, metabolism, cellular response to homeostasis and ROS detoxification. This analysis is supported by the earlier study that Fe-binding proteins have profound effect on growth, metabolism and survival of *P*. *syringae* (Kim et al., 2009).

The functional classification of Zn-binding proteins showed that metabolic process, transport, cell signaling and protein biosynthesis were most enriched functional categories. The cytoplasmic proteins with domains SDR and ADH/GroES were commonly found in metabolic process. SDR have oxidoreductase activity to catalyze amino acid, carbohydrate, lipid and xenobiotic metabolism (Kavanagh et al., 2008). ADH/GroES also catalyzes oxidation-reduction reactions during alcohol fermentation and its Gro-ES like fold helps in maintaining its structural integrity (Zheng et al., 2017). The transport category of Zn-binding proteins also showed the main presence of integral membrane ABC transporter and periplasmic SBPs domain like Fe-binding proteins. As discussed above both of these domains help in transport of metal ions and other substrates (Tanaka et al., 2017). The proteins in the cell signaling category mainly contain response regulator receiver domain which influences a cellular response through phosphorylation-activated switches (Gao et al., 2007). Elongation factor Tu GTP binding domain which causes GTP dependent elongation of polypeptide chain during translation and tRNA synthase which catalyzes the amino acid addition to its cognate tRNA were majorly present in the category of protein biosynthesis (Andersen et al., 2003; Banerjee et al., 2004). The GO enriched BP network analysis of Zn-binding proteins showed the conformity with functional domain-based classification which revealed that most of the proteins involved in metabolic processes (protein, purine, organic acid, DNA and RNA), transport (ions, amino acid and transition metals), tRNA aminoacylation, amino acid activation and translation elongation. The analysis was also in accordance with the previous findings that Zn-binding proteins play a wide role in various biological processes and contribute in bacterial growth and proliferation (Murakami and Hirano, 2008; Maret, 2013).

The functional classification of Cu-binding proteins was enriched in the categories of metabolic process, response to oxidative stress and transport. Several metalloproteins were reported to be highly promiscuous in nature and could bind with one or more metal ions (Pordea, 2015). The proteins found in metabolic process mainly contain ADH/GroES and Glutathione S-transferase (GST) and Carbonic anhydrase (CA) domains. Both ADH/GroES and CA protein were reported as Zn-binding metalloenzymes (Christianson and Fierke, 1996; Bergman et al., 2008). The role of ADH/GroES in alcohol fermentation has already discussed above. CA involved in reversible hydration of carbon dioxide and causes interference in pH regulation other physiological processes of bacteria (Lionetto et al., 2016). However, no *in vivo* study validates the binding of Cu ion to ADH and CA. Earlier *in vitro* studies showed that Cu ion can substitute Zn ion in ADH (Vanni et al., 2002) and CA (Nettles et al., 2015). Since, Cu is able to substitute Zn from its binding site, therefore, it can be inferred that the proteins may have affinity for Cu ions as well (Nettles et al., 2015). GST catalyzes the nucleophilic addition of tripeptide glutathione to the substrate having reactive electrophilic functional groups which aid in biodegradative metabolism of xenobiotic compounds and their detoxification (Armstrong, 1991). Although, there are no direct evidences that Cu ion bind to GST, but biochemical studies showed that GST proteins have binding affinity for Cu ion (Tang et al., 1996; Chen et al., 2015). The activity of GST is inhibited by Cu-binding which leads to inactivation of enzyme. This enzyme inactivation is dose (Cu concentration) and time-dependent (Tang et al., 1996; Shi et al., 2014). It is still not clear whether direct binding of Cu to GST have a significant role in Cu-homeostasis or not which can be future thirst area. In the category of response to oxidative stress, most of the Cu-binding proteins belong to periplasmic multicopper oxidase (MCO), Copper/zinc superoxide dismutase (Cu/Zn SODs) domain azurin and alkyl hydroperoxide reductase/thiol-specific antioxidant (AhpC/TSA) family. MCO catalyzes oxidation-reduction reactions to maintain oxidative stress response (Sitthisak et al., 2005). Cu/Zn SODs have dismutase activity which catalyzes the conversion of super-radicals to H_2_O_2_ and molecular oxygen (Fones and Preston, 2012). Azurin also acts as redox sensor and protect the cell from radical burst (Swingle et al., 2008). Earlier a protein of AhpC/TSA family has been reported in *Pseudomonas putida* which have specificity for Cu ion and have antioxidant activity to encounter oxidative stress (Miller et al., 2009). The inner-membrane P-type ATPases, and cytochrome c oxidase (COX) domains were mainly found in transport category of the Cu-binding proteins. The inner-membrane P-type ATPases of bacteria involved in the ATP dependent export of Cu ion during Cu toxicity in their cytoplasm which aid in maintaining Cu- homeostasis (Arguello et al., 2011). The COX enzyme is the last enzyme in electron transport chain, it receives the electron from cytochrome c molecules and transport them to oxygen coupled to proton translocation, which results in the reduction of oxygen to water (Ludwig, 1987). The GO enriched BP analysis of Cu-binding proteins confirms their involvement in the regulation of metabolic processes, redox homeostasis and response to reactive species which is also in accordance with their functional domain analysis.

The importance of metalloproteins in overall life processes makes their availability essential to all the organisms. But the excess of these metal ions is toxic to cell and therefore, metal ions concentration must be controlled tightly (Chandrangsu et al., 2017). Previously, it was documented that secreted proteins are major virulence factor of the bacteria which disrupt the normal functions of the host after interactions with the receptors of the defense system of the host (Ichinose et al., 2013). The metal ions uptake, storage and utilization also contribute to the bacterial virulence. Earlier, it was reported that the metal ions co-regulate the expression of secreted proteins and endorse their functions of virulence (Sharma et al., 2017). In the present study, we have identified putative Fe, Zn, and Cu-binding secreted proteins which were exclusively present in *P. syringae* pv. *lapsa* but not in host (*Triticum aestivum*). So, these candidate metalloproteins may act as suitable targets to design metal-based antimicrobial agents.

The whole proteome of *P. syringae* pv. *lapsa* comprised of 29, 31 and 5 putative secreted Fe, Zn, and Cu-binding proteins respectively that varies in function from transport, response to oxidative stress, proteolysis, antimicrobial resistance, metabolic process, protein folding and DNA repair. We have found 39 transporter proteins, out of these 14 proteins were predicted as outer-membrane TBDRs proteins. As discussed above TBDRs may involve in the transport of Fe-siderophore complexes and help in maintaining Fe-homeostasis in bacteria. Earlier, a link has already been confirmed between Fe-siderophore uptake, biofilm formation, quorum sensing and virulence during plant-pathogen interactions (Taguchi et al., 2010). Among 39 transporter proteins, 18 were SBPs. Out of these 18 SBPs, 11 proteins have an affinity for Zn, 2 have affinity for Fe while 4 proteins have capability to bind with both Fe and Zn and 1 protein have affinity for Fe and Cu ion. It was also reported that SBPs act as receptors for import of metal ions that contribute in metal-homeostasis and virulence of pathogenic bacteria (Tam and Saier, 1993; Tai et al., 2003; Handali et al., 2015). In agro-bacterial species, the periplasmic binding proteins (PBPs) are needed for metal-homeostasis, virulence and extracellular signaling between bacterial parasite and the host plant (Cangelosi et al., 1990; Shouldice et al., 2004; Chandra et al., 2007). We have identified 5 PBPs which may play important role in the transport of ions. Out of 5 PBPs, 3 have the capability to bind Fe and 2 proteins have an affinity for Zn ion. Two periplasmic adhesion lipoproteins have also been found in the class of transport. Earlier studies provide evidences that adhesion lipoproteins help in transport of Fe ions, attachment and adherence of the bacteria into the host and contribute in virulence (Berne et al., 2015; Ghazaei, 2016). The structural analysis of putatively secreted transporter metalloproteins showed that the most common interacting residues were Tyr, Phe, Trp, Asp, Lys and Ala in the binding site of TBDRs, SBPs and PBPs, which were similar to their template structures. These results support the previous finding that aromatic residues contribute in membrane protein interactions (Cobessi et al., 2005).

We have found 4 putative secreted metalloproteins in the category of response to oxidative stress. One of which is a Fe-binding aconitase hydratase extracellular protein. The aconitase enzyme contains iron-sulfur cluster involved in TCA cycle and their role in the protection from ROSs has also been reported earlier in *Xanthomonas campestris* pv. *vesicatoria* (Kirchberg et al., 2012). A Zn-binding protein belongs to DJ/Pfpl family were also found to respond to oxidative stress. Earlier reports indicate that the DJ/Pfpl family containing proteins have a crucial role in redox homeostasis regulation (Bankapalli et al., 2015). The other two proteins involved in response to oxidative stress have binding affinity for both Cu and Zn ions. One of which has Cu/Zn SODs and another one has azurin domain. As discussed above the Cu/Zn SODs are well known for their high tolerance to oxidative stress and defense activities (Fones and Preston, 2012). It was also previously published that periplasmic azurin protein co-regulate the function of the gene needed for siderophore synthesis, response to oxidative stress and therefore, it may contribute to the virulence (Swingle et al., 2008).

In the category of proteolysis, we have found 3 extracellular and 1 periplasmic putatively secreted metalloproteins. Out of 3 extracellular proteins, one was thermolysin metallopeptidase (Peptidase M4) and other two were peptidase M10 serrlysin proteins. The thermolysin metallopeptidase protein has binding site for both Fe and Zn metal ions (Holland et al., 1995). Earlier findings showed that thermolysin proteases play important role in the degradation of peptic cellwall component of host plant and aid in virulence of the phytopathogen (Kyostio et al., 1991). The peptidase M10 serrlysin is already known for its Zn-binding affinity and ability to degrade and colonize the host cell (Zhang et al., 1999). A periplasmic putative secreted protein belongs to trypsin family was also found in the class of proteolysis which has an affinity for both Zn as well as Cu. It was previously reported that trypsin requires metal ion for its structural stability, induces host cell lysis and further help to raise the virulence of bacteria (Kyostio et al., 1991; Tripathi and Sowdhamini, 2008; Li et al., 2018).

We have identified 5 putative secreted Zn-binding proteins in the class of antimicrobial resistance. One of them was periplasmic β-lactamase protein, and other 4 were outer-membranic peptidase M23 proteins. Earlier studies suggested that β-lactamase and peptidase M23 domain containing proteins play important role in antimicrobial resistance by interfering with the synthesis of peptidoglycan of the cell wall (Bebrone, 2007; Grabowska et al., 2015).

We have found two periplasmic proteins involved in metabolic processes. Among them, one was a periplasmic Fe-binding intradiol ring-cleavage dioxygenase which may be involved in metabolic degradation of aromatic compounds. It was previously documented that intradiol deoxygenase destroys the precursors required for lignin biosynthesis by the plant (Bianchetti et al., 2013) and aid in the invasion of the phytopathogen. The other protein was periplasmic Zn-binding isochorismatase which may be involved in the regulation of metabolic pathways. It is well-known fact that the isochroismatase proteins disrupt the plant salicylate metabolism by restraining the activity of its precursor and play indispensable role in the plant-pathogen interactions (Liu et al., 2014). We have identified 1 periplasmic single-strand Zn-binding proteins which may be involved in DNA repair and transformation. Our findings are supported by the earlier study that single-strand binding proteins are essential for T-strand production, DNA repair and virulence (Citovsky et al., 1988). We have found a periplasmic copper chaperone SCO1/SenC in the class of protein folding. Copper chaperone SCO1/SenC is primarily required for the proper assembly of COX (Lohmeyer et al., 2012). Additionally, the thiol:disulfide oxidoreductase or peroxiredoxin activity of SCO1/SenC helps in protection of bacteria against oxidative stress (Seib et al., 2003). This comprehensive *in silico* report on Fe, Zn and Cu-binding proteins of *P. syringae* pv. *lapsa* provide us evidence that metalloproteins, belonging to various protein families perform diverse range of biological functions and play crucial role in its normal growth, survival and pathogenesis. The obtained results related to secreted metalloproteins could be further investigated in more details by experimental approaches. This will further help to understand their mechanistic insight in plant-pathogen interactions, to control phytopathogenic infections in the crop plants and to develop sustainable agriculture.

## Conclusion

In the present study a systemic bioinformatics approach has been used to predict the Fe, Zn, and Cu-binding proteome of *P. syringae* pv. *lapsa*, at the sequence and structural levels. It was observed that the identified metalloproteins perform a diverse range of biochemical and metabolic functions. This signifies the fact that metalloproteins are vital for growth and proliferation of *P. syringae* pv. *lapsa*. The study also provides the repository of putative secreted Fe, Zn, and Cu-binding proteins which may serve as the primer to perform the experimental validation. This will help to understand the role of putative secreted metal binding proteins in plant–pathogen interactions and to develop new strategies to control plant disease which may further accelerate the healthy agricultural yield.

## Author Contributions

SV and AS conceived the idea. AS has done identification of metalloproteins, curation and analysis of the data. AS and DS performed the structural analysis. AS wrote the manuscript. SV edited the manuscript.

## Conflict of Interest Statement

The authors declare that the research was conducted in the absence of any commercial or financial relationships that could be construed as a potential conflict of interest.

## Footnotes

1 ftp://ftp.ncbi.nlm.nih.gov/genomes/all/GCF/001/482/725/GCF_001482725.1_ASM148272v1
