# Supplementary material for: In silico Study of Iron, Zinc and Copper Binding Proteins of Pseudomonas syringae pv. lapsa: Emphasis on Secreted Metalloproteins
Source: Front Microbiol. 2018 Aug 21;9:1838. doi: 10.3389/fmicb.2018.01838 (PMC6110883; doi:10.3389/fmicb.2018.01838)
Supplement: Supplementary file 1 [file Data_Sheet_1.docx]

Supplementary Material

***In silico* study of iron, zinc and copper binding proteins of *Pseudomonas syringae* pv. *lapsa*: emphasis on secreted metalloproteins**

Ankita Sharma^1^, Dixit Sharma^1^, Shailender Kumar Verma^1#^*

^1^School of Life Sciences, Central University of Himachal Pradesh, Shahpur, Kangra, Himachal Pradesh, 176206, India

**# Present Address**

Department of Biological Chemistry, John Innes Centre, Norwich, NR4 7UH, United Kingdom

*** Correspondence:**

Dr. Shailender Kumar Verma

[skverma@cuhimachal.ac.in](mailto:skverma@cuhimachal.ac.in), [skverma.cuhl@nic.in](mailto:skverma.cuhl@nic.in)

# Supplementary Data

Supplementary data is in form of figures and tables.

# Supplementary Figures and Tables

## Supplementary Figures


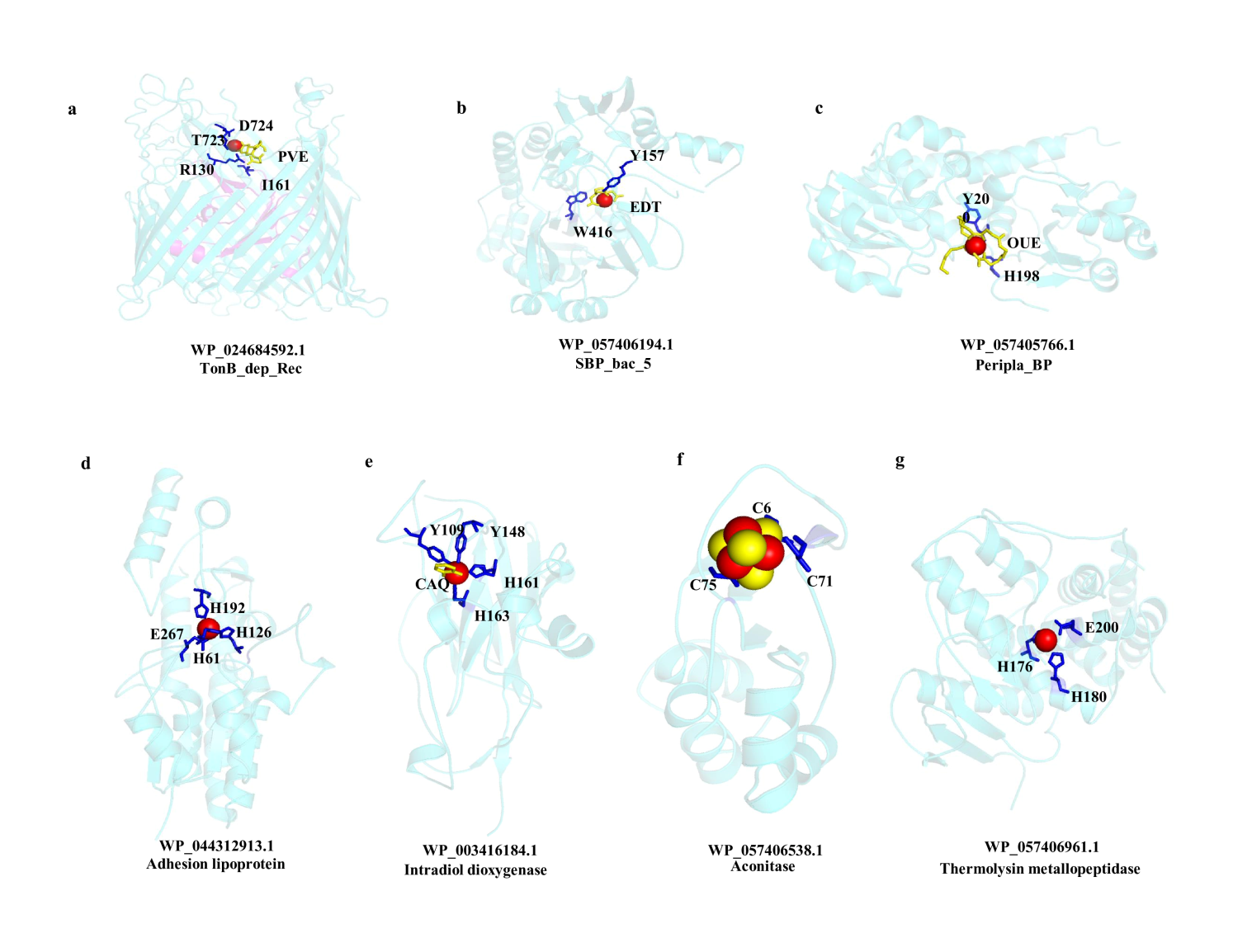


**Supplementary Figure 1 Representative tertiary structure of each family of putative secretory iron binding proteins:**

All the proteins are shown with cartoon model (cyan) and the iron metal is shown as sphere (red). The ligands are shown with stick and colored yellow. The binding residues with iron metal ion and metal-ligand complexes are shown as blue sticks. The sequence Id and representative family of all the proteins are mentioned below their structure. **Transport: (a)** WP_024684592.1 protein of TonB dep Rec family with Fe-Pyoverdine (PVE) complex. **(b)** WP_057406194.1 of SBP bac 5 family with Fe-({[-BIS-CARBOXYMETHYL-AMINO)-ETHYL]-CARBOXYMETHYL-AMINO}-ACETIC ACID (EDT) complex. **(c)** WP_057405766.1 of Peripla BP family with Fe-Ferrioxamine B (OUE) complex. **(d)** WP_044312913.1 of adhesion lipoprotein family**.** **Metabolic Process: (e)** WP_003416184.1 of intradiol dioxygenase family with Fe-Catechol (CAQ. **Response to oxidative stress : (f)** WP_057406538.1 of Aconitase hydratase family with iron-sulphur cluster (red-yellow) sphere. **Proteolysis:** **(g)** WP_057406961.1 of thermolysin metallopeptidase family.


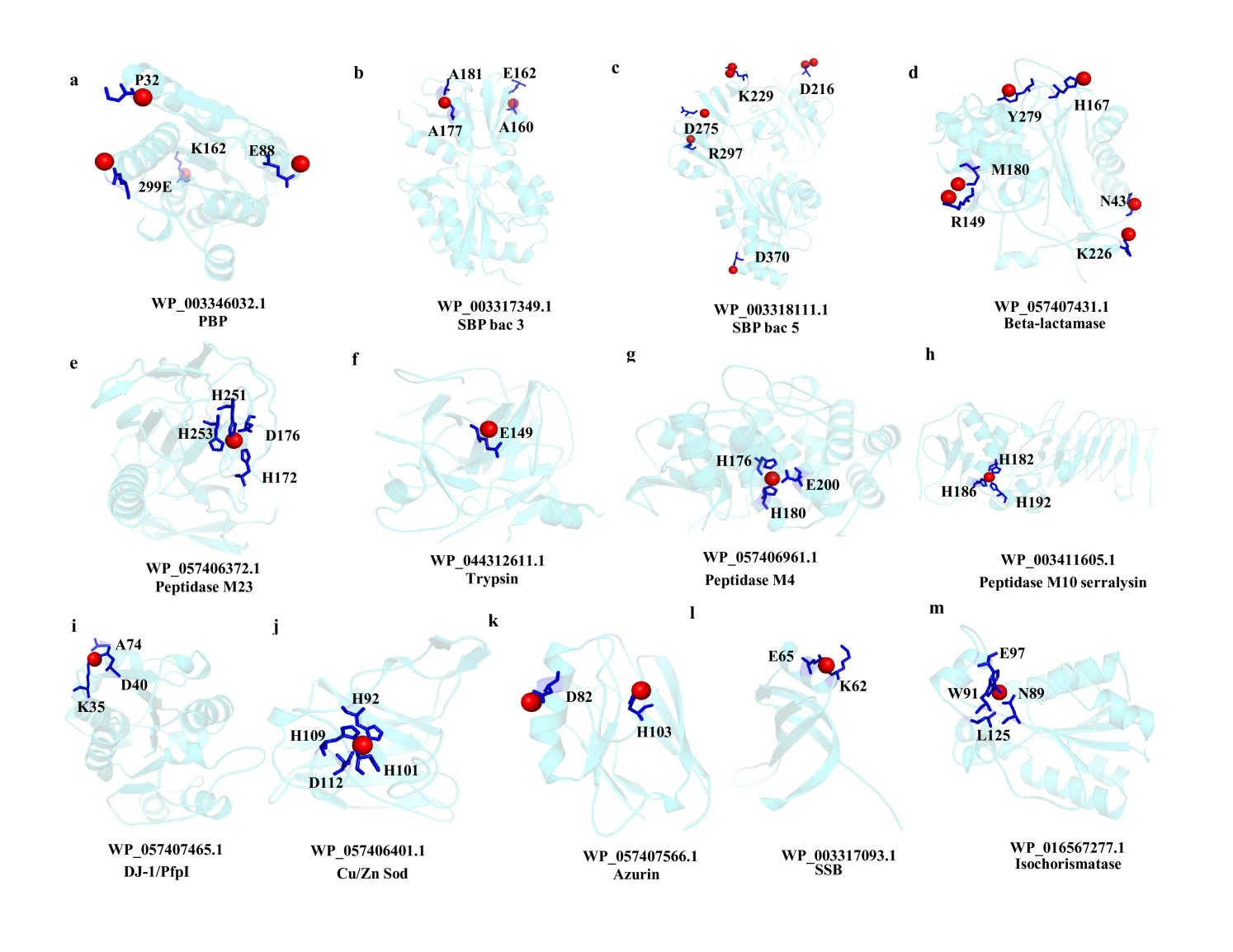


**Supplementary Figure 2 The representative tertiary structure of each family of putative secretory zinc binding proteins.** All the proteins are shown with cartoon model (cyan).The zinc metal is shown as sphere (red) and the interacting residues with zinc metal ion are shown with blue sticks. The sequence id and representative family of all the proteins are mentioned below their structure. **Transport: (a)** WP_003346032.1 protein of Peripla BP family, **(b)** WP_003317349.1 protein of SBP bac 3 family, **(c)** WP_003318111.1 protein of SBP bac 5 family. **Antimicrobial resistance: (d)** WP_057407431.1 protein of Beta-lactamase family, **(e)** WP_057406372.1 protein of Peptidase M23 family. **Proteolysis: (f)** WP_044312611.1 protein of Trypsin family**, (g)** WP_057406961.1 protein of Thermolysin metallopeptidase family, **(h)** WP_003411605.1 protein of Peptidase M10 serralysin family. **Response to oxidative stress: (i)** WP_057407465.1 protein of DJ-1/PfpI family, **(j)** WP_057406401.1 protein of Cu/Zn Sod family, **(k)** WP_057407566.1 protein of Azurin family. **DNA repair** (l) WP_003317093.1 protein of SSB family. **Metabolic pathway (m)** WP_016567277.1 protein of Isochorismatase family.

**
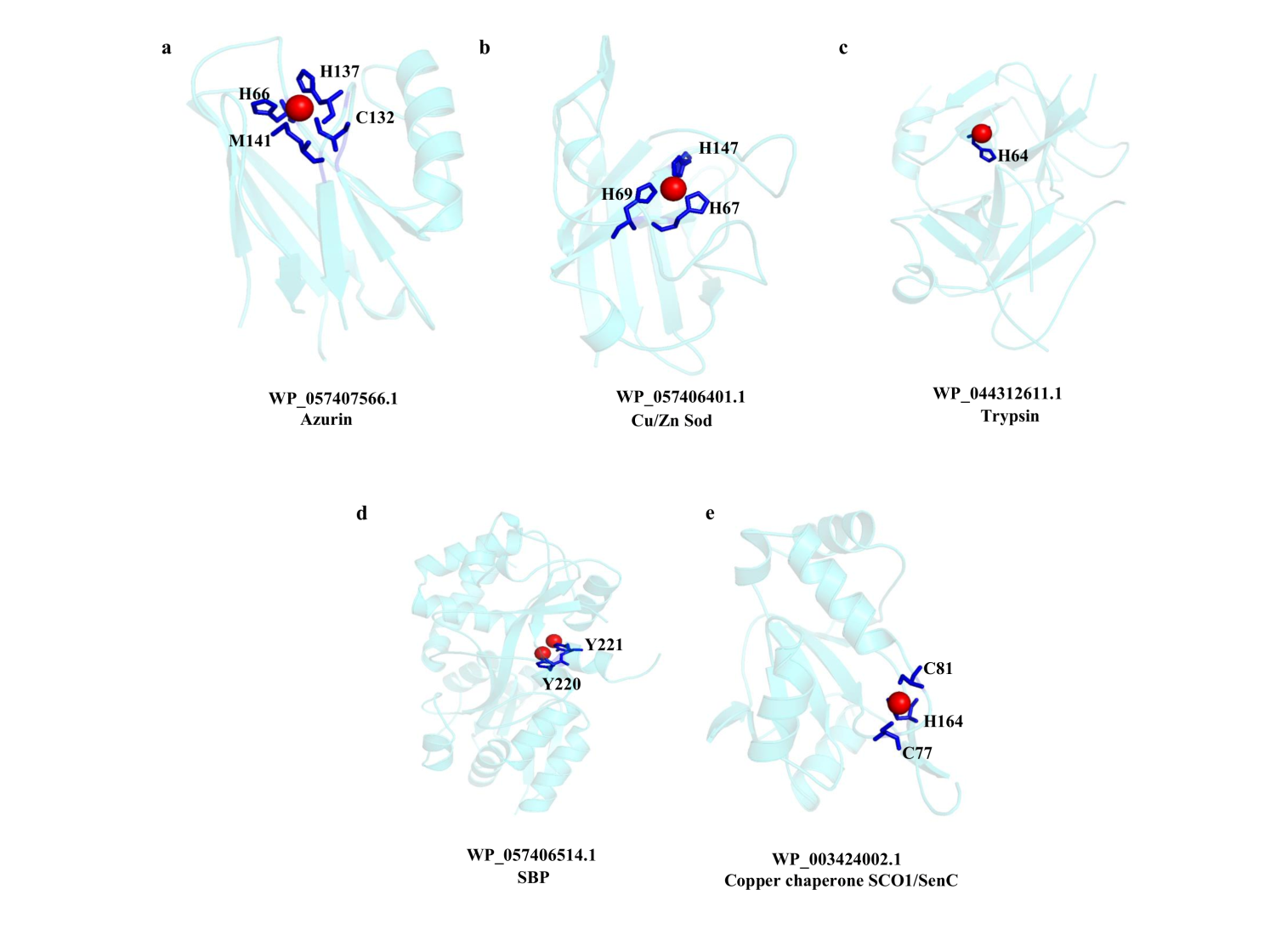
**

**Supplementary Figure 3 Representative tertiary structure of secretory copper binding proteins.**

All the proteins are shown with cartoon model (cyan).The copper metal is shown as sphere (red) and the interacting residues with copper metal ion are shown with blue sticks. The sequence id and representative family of all the proteins are mentioned below their structure. **Response to oxidative stress** **(a)** WP_057407566.1 protein of Azurin family, **(b)** WP_057406401.1 protein of Cu/Zn Sod family. **Proteolysis: (c)** WP_044312611.1 of trypsin family. **Transport (d)** WP_057406514.1 protein of SBP bac family. **Protein folding (e)** WP_003424002.1 protein of copper chaperone SCO1/SenC family.

**2.2 Supplementary Tables**

**Supplementary Table S1 : Subcellular localization and Functional domain analysis of iron binding proteins**

| **S. No.** | **Sequence ID** | **Subcellular localization** | **Functional domain/family** | **Description** | **Broad class** |
| --- | --- | --- | --- | --- | --- |
| 1 | WP_057407492.1 | Cytoplasmic | Metallo-beta-lactamase | Hydrolyze the amide bond in beta-lactam compounds | Antimicrobial resistance |
| 2 | WP_057407454.1 | InnerMembrane | GGDEF domain | Catalyze synthesis or hydrolysis of cyclic diguanylate | Cell signaling |
| 3 | WP_057406495.1 | Cytoplasmic | HD domain | Have phosphohydrolase activity and are involved in signal transduction | Cell signaling |
| 4 | WP_003344947.1 | InnerMembrane | PAS domain | Signal sensor for light, oxygen and ligands | Cell signaling |
| 5 | WP_057406934.1 | Periplasmic | Alpha-ketoglutarate-dependent dioxygenase AlkB-like | Removal of alkylation lesions from purine and pyrimidine at endocyclic positions | DNA repair |
| 6 | WP_044311542.1 | Cytoplasmic | AP endonuclease | Create a nick in the phosphodiester backbone of the AP site | DNA repair |
| 7 | WP_044312066.1 | Cytoplasmic | HhH-GPD | Found in variety of enzyme involved in DNA repair mechanism | DNA repair |
| 8 | WP_057406193.1 | Cytoplasmic | Metallo-dependent phosphatase-like | Hydrolyse phosphomonoesters, phosphodiesters and phophotriesters in a metal dependent manner | DNA repair |
| 9 | WP_057407268.1 | Cytoplasmic | Metallo-dependent phosphatase-like | Hydrolyse phosphomonoesters, phosphodiesters and phophotriesters in a metal dependent manner | DNA repair |
| 10 | WP_057407599.1 | Cytoplasmic | Metallo-dependent phosphatase-like | Hydrolyse phosphomonoesters, phosphodiesters and phophotriesters in a metal dependent manner | DNA repair |
| 11 | WP_003318412.1 | Cytoplasmic | UvrD-like helicase | ATP dependent enzyme helps in unwinding of duplexes DNA in 3'-5' polarity | DNA repair |
| 12 | WP_003367943.1 | Cytoplasmic | UvrD-like helicase | ATP dependent enzyme helps in unwinding of duplexes DNA in 3'-5' polarity | DNA repair |
| 13 | WP_003368718.1 | Cytoplasmic | [2Fe-2S] binding domain | Found in variety of enzymes like oxidoreductases, dehydrogenases and oxidases which are involved in many metabolic processes | Metabolic process |
| 14 | WP_057407038.1 | Cytoplasmic | [2Fe-2S] binding domain | Found in variety of enzymes like oxidoreductases, dehydrogenases and oxidases which are involved in many metabolic processes | Metabolic process |
| 15 | WP_003316554.1 | InnerMembrane | 2Fe-2S iron-sulfur cluster binding domain | Mediate electron transfer in numerous metabolic processes | Metabolic process |
| 16 | WP_057406593.1 | Cytoplasmic | 2OG-Fe(II) oxygenase superfamily | Found in many enzymes to catalyses oxidation-reduction reactions | Metabolic process |
| 17 | WP_003316325.1 | Cytoplasmic | 4Fe-4S ferredoxin-type iron-sulfur binding domain | Mediate electron transfer in numerous metabolic processes | Metabolic process |
| 18 | WP_003339547.1 | Cytoplasmic | 4Fe-4S ferredoxin-type iron-sulfur binding domain | Mediate electron transfer in numerous metabolic processes | Metabolic process |
| 19 | WP_003316525.1 | Cytoplasmic | Aconitase family, 4Fe-4S cluster binding site | Reversible isomerization of citrate and isocitrate in TCA cycle | Metabolic process |
| 20 | WP_024661529.1 | Cytoplasmic | Aconitase family, 4Fe-4S cluster binding site | Reversible isomerization of citrate and isocitrate in TCA cycle | Metabolic process |
| 21 | WP_057405827.1 | Cytoplasmic | Aconitase family, 4Fe-4S cluster binding site | Reversible isomerization of citrate and isocitrate in TCA cycle | Metabolic process |
| 22 | WP_057407397.1 | Cytoplasmic | Aconitase family, 4Fe-4S cluster binding site | Reversible isomerization of citrate and isocitrate in TCA cycle | Metabolic process |
| 23 | WP_003314266.1 | Cytoplasmic | Adenylate kinase | Nucleotide phosphoryl exchange reaction | Metabolic process |
| 24 | WP_044311859.1 | Cytoplasmic | Adrenodoxin | Have electron transfer activity and play role biosynthesis of setroid | Metabolic process |
| 25 | WP_003392017.1 | Cytoplasmic | Aminotransferase class-III | Have pyridoxal phosphate binding and transaminase activity which are involve in various biosynthetic processes | Metabolic process |
| 26 | WP_003402943.1 | Cytoplasmic | Aminotransferase class-III | Have pyridoxal phosphate binding and transaminase activity which are involve in various biosynthetic processes | Metabolic process |
| 27 | WP_004411082.1 | Cytoplasmic | Aminotransferase class-III | Have pyridoxal phosphate binding and transaminase activity which are involve in various biosynthetic processes | Metabolic process |
| 28 | WP_004417709.1 | Cytoplasmic | Aminotransferase class-III | Have pyridoxal phosphate binding and transaminase activity which are involve in various biosynthetic processes | Metabolic process |
| 29 | WP_044312440.1 | Cytoplasmic | Aminotransferase class-III | Have pyridoxal phosphate binding and transaminase activity which are involve in various biosynthetic processes | Metabolic process |
| 30 | WP_057405800.1 | Cytoplasmic | Aminotransferase class-III | Have pyridoxal phosphate binding and transaminase activity which are involve in various biosynthetic processes | Metabolic process |
| 31 | WP_057405887.1 | Cytoplasmic | Aminotransferase class-III | Have pyridoxal phosphate binding and transaminase activity which are involve in various biosynthetic processes | Metabolic process |
| 32 | WP_057406018.1 | Cytoplasmic | Aminotransferase class-III | Have pyridoxal phosphate binding and transaminase activity which are involve in various biosynthetic processes | Metabolic process |
| 33 | WP_057406163.1 | Cytoplasmic | Aminotransferase class-III | Have pyridoxal phosphate binding and transaminase activity which are involve in various biosynthetic processes | Metabolic process |
| 34 | WP_057407034.1 | Cytoplasmic | Aminotransferase class-III | Have pyridoxal phosphate binding and transaminase activity which are involve in various biosynthetic processes | Metabolic process |
| 35 | WP_003409112.1 | Cytoplasmic | Aminotransferase class-V | Have pyridoxal phosphate binding and transaminase activity which are involve in various biosynthetic processes | Metabolic process |
| 36 | WP_003423469.1 | Cytoplasmic | ARD/ARD' family | Catalyses oxidation-reduction process in methionine salvage pathway | Metabolic process |
| 37 | WP_004415612.1 | Cytoplasmic | Aromatic-ring-hydroxylating dioxygenase, alpha subunit | Conversion of closed ring aromatic complexes to non-aromatic cis-diols | Metabolic process |
| 38 | WP_057405905.1 | Cytoplasmic | Biopterin-dependent aromatic amino acid hydroxylase | Utilizes tetrahyrobiopterin as substrate for ring hydroxylation of aromatic amino acids | Metabolic process |
| 39 | WP_003316190.1 | Cytoplasmic | Biotin and Thiamin Synthesis associated domain | Biosynthesis of thiamine diphosphate | Metabolic process |
| 40 | WP_032660413.1 | Cytoplasmic | CO dehydrogenase flavoprotein | Oxidation of CO with water to yield CO_2_ and electron for electron transport chain | Metabolic process |
| 41 | WP_057405639.1 | Cytoplasmic | Cysteine dioxygenase type I | Catalyses oxidation-reduction reactions to maintain cysteine homeostasis and to regulate sulphate production | Metabolic process |
| 42 | WP_057405902.1 | Cytoplasmic | DegT/DnrJ/EryC1/StrS aminotransferase family | Have pyridoxal phosphate binding and transaminase activity which are involve in various biosynthetic processes | Metabolic process |
| 43 | WP_057405945.1 | Cytoplasmic | DegT/DnrJ/EryC1/StrS aminotransferase family | Have pyridoxal phosphate binding and transaminase activity which are involve in various biosynthetic processes | Metabolic process |
| 44 | WP_057406817.1 | Cytoplasmic | DegT/DnrJ/EryC1/StrS aminotransferase family | Have pyridoxal phosphate binding and transaminase activity which are involve in various biosynthetic processes | Metabolic process |
| 45 | WP_003346110.1 | Cytoplasmic | Dihydroorotate dehydrogenase | Oxidation of dihyroorolate to orolate in fourth step of de novo pyrimidine biosynthesis | Metabolic process |
| 46 | WP_003422526.1 | Cytoplasmic | Dihydroprymidine dehydrogenase domain II, 4Fe-4S cluster | Involve in first i.e rate limiting step of pyrimidine degradation | Metabolic process |
| 47 | WP_057407202.1 | Cytoplasmic | Ferredoxin reductase-type FAD-binding domain | Catalyses dehydrogenation of metabolites, oxidation-reduction reactions and electron transfer | Metabolic process |
| 48 | WP_004410832.1 | Cytoplasmic | Glutamate synthase domain | Reductive biosynthesis of L-glutamate from 2-oxoglutarate and L-glutamine | Metabolic process |
| 49 | WP_003347797.1 | Cytoplasmic | Glutamine amidotransferase domain | Formation of new carbon nitrogen group by removing and transferring ammonia group from glutamine to other substrate | Metabolic process |
| 50 | WP_057407460.1 | Cytoplasmic | Homogentisate 1,2-dioxygenase | Catalyzes oxidation and reduction reaction to convert homogentisate to maleylacetoacetate in phenyalanine and tyrosine catabolism | Metabolic process |
| 51 | WP_046719726.1 | Cytoplasmic | Hydroxyacylglutathione hydrolase C-terminus | Catalyses last step in methylgyoxal conversion to lactic acid and reduced glutathione | Metabolic process |
| 52 | WP_003315852.1 | Cytoplasmic | Hydroxyphenylpyruvate dioxygenase, HPPD | Conversion of hydroxyphenylpyruvate to homogentisate | Metabolic process |
| 53 | WP_003416184.1 | Periplasmic | Intradiol ring-cleavage dioxygenase | Catalyses oxidation reduction reactions to cleave aromatic ring during aromatic compounds degradation | Metabolic process |
| 54 | WP_010424090.1 | Cytoplasmic | Iron-containing alcohol dehydrogenase | Reversible oxidation of ethanol to acetaldehyde and reduction of NAD | Metabolic process |
| 55 | WP_057407211.1 | Cytoplasmic | Iron-containing alcohol dehydrogenase | Reversible oxidation of ethanol to acetaldehyde and reduction of NAD | Metabolic process |
| 56 | WP_057405774.1 | Cytoplasmic | Isopenicillin N synthase-like | Formation of Isopenicillin N which is a precursor for biosynthesis of beta-lactam antibiotics | Metabolic process |
| 57 | WP_057406879.1 | Cytoplasmic | Isopenicillin N synthase-like | Formation of Isopenicillin N which is a precursor for biosynthesis of beta-lactam antibiotics | Metabolic process |
| 58 | WP_057406478.1 | Cytoplasmic | L-lactate dehydrogenase, bacterial | Oxidative conversion of L-lactate to pyruvate | Metabolic process |
| 59 | WP_003424754.1 | Cytoplasmic | Metal-dependent hydrolase | Hydrolytic cleavage of wide spectrum of substrates | Metabolic process |
| 60 | WP_057405765.1 | Cytoplasmic | Metal-dependent hydrolase | Hydrolytic cleavage of wide spectrum of substrates | Metabolic process |
| 61 | WP_057405846.1 | Cytoplasmic | Metal-dependent hydrolase | Hydrolytic cleavage of wide spectrum of substrates | Metabolic process |
| 62 | WP_057406366.1 | Cytoplasmic | Metal-dependent hydrolase | Hydrolytic cleavage of wide spectrum of substrates | Metabolic process |
| 63 | WP_057406751.1 | Cytoplasmic | Metal-dependent hydrolase | Hydrolytic cleavage of wide spectrum of substrates | Metabolic process |
| 64 | WP_057407607.1 | Cytoplasmic | Metal-dependent hydrolase | Hydrolytic cleavage of wide spectrum of substrates | Metabolic process |
| 65 | WP_003393669.1 | Cytoplasmic | Oxidoreductase FAD/NAD(P)-binding | Present in various enzymes to catalyse oxidation-reduction reactions | Metabolic process |
| 66 | WP_057406498.1 | Cytoplasmic | Oxidoreductase FAD/NAD(P)-binding | Present in various enzymes to catalyse oxidation-reduction reactions | Metabolic process |
| 67 | WP_057406554.1 | Cytoplasmic | Oxidoreductase FAD/NAD(P)-binding | Present in various enzymes to catalyse oxidation-reduction reactions | Metabolic process |
| 68 | WP_057406216.1 | Cytoplasmic | Phosphotriesterase family | Catabolic activity on synthetic organophosphate triesters and phosphofluoridates | Metabolic process |
| 69 | WP_057405836.1 | Cytoplasmic | Protocatechuate 3,4-dioxygenase, alpha subunit | Aromatic amino acid cleavage to degrade plant biopolymers | Metabolic process |
| 70 | WP_003317890.1 | Cytoplasmic | Radical_SAM | Reductive cleavage of SAM to produce free radicals and to catalyse variety of metabolic activities | Metabolic process |
| 71 | WP_002554639.1 | Cytoplasmic | Ribonucleotide reductase, small chain | Reductive synthesis of deoxyribonucleotides from ribonucleotides which are used as precursors for DNA synthesis | Metabolic process |
| 72 | WP_057406304.1 | Cytoplasmic | Ribonucleotide reductase, small chain | Reductive synthesis of deoxyribonucleotides from ribonucleotides which are used as precursors for DNA synthesis | Metabolic process |
| 73 | WP_003316551.1 | InnerMembrane | Succinate dehydrogenase/Fumarate reductase transmembrane subunit | Catalyses oxidation-reduction reactions | Metabolic process |
| 74 | WP_003423585.1 | InnerMembrane | Succinate dehydrogenase/Fumarate reductase transmembrane subunit | Catalyses oxidation-reduction reactions | Metabolic process |
| 75 | WP_024665695.1 | Cytoplasmic | Taurine catabolism dioxygenase TauD, TfdA family | Catalyses oxygenolytic discharge of sulphite from taurine during sulphate starvation | Metabolic process |
| 76 | WP_024959042.1 | Cytoplasmic | Taurine catabolism dioxygenase TauD, TfdA family | Catalyses oxygenolytic discharge of sulphite from taurine during sulphate starvation | Metabolic process |
| 77 | WP_057406034.1 | Cytoplasmic | Taurine catabolism dioxygenase TauD, TfdA family | Catalyses oxygenolytic discharge of sulphite from taurine during sulphate starvation | Metabolic process |
| 78 | WP_057406049.1 | Cytoplasmic | Taurine catabolism dioxygenase TauD, TfdA family | Catalyses oxygenolytic discharge of sulphite from taurine during sulphate starvation | Metabolic process |
| 79 | WP_057406566.1 | Cytoplasmic | Taurine catabolism dioxygenase TauD, TfdA family | Catalyses oxygenolytic discharge of sulphite from taurine during sulphate starvation | Metabolic process |
| 80 | WP_057407032.1 | Cytoplasmic | Taurine catabolism dioxygenase TauD, TfdA family | Catalyses oxygenolytic discharge of sulphite from taurine during sulphate starvation | Metabolic process |
| 81 | WP_003368889.1 | Cytoplasmic | Urease, alpha subunit | Hydrolysis of urea to ammonia and carbamate | Metabolic process |
| 82 | WP_003313490.1 | Cytoplasmic | NADH:flavin oxidoreductase / NADH oxidase family | Have FMN binding and oxidoreductase activity to regulate various metabolic processes | Metabolic process |
| 83 | WP_004415601.1 | Cytoplasmic | NADH:flavin oxidoreductase / NADH oxidase family | Have FMN binding and oxidoreductase activity to regulate various metabolic processes | Metabolic process |
| 84 | WP_024647946.1 | Cytoplasmic | NADH:flavin oxidoreductase / NADH oxidase family | Have FMN binding and oxidoreductase activity to regulate various metabolic processes | Metabolic process |
| 85 | WP_057405588.1 | Cytoplasmic | NADH:flavin oxidoreductase / NADH oxidase family | Have FMN binding and oxidoreductase activity to regulate various metabolic processes | Metabolic process |
| 86 | WP_057406128.1 | Cytoplasmic | NADH:flavin oxidoreductase / NADH oxidase family | Have FMN binding and oxidoreductase activity to regulate various metabolic processes | Metabolic process |
| 87 | WP_057406920.1 | Cytoplasmic | NADH:flavin oxidoreductase / NADH oxidase family | Have FMN binding and oxidoreductase activity to regulate various metabolic processes | Metabolic process |
| 88 | WP_057406328.1 | Cytoplasmic | Polypeptide deformylase | Formyl group removal from N-terminal of polypeptide chain | Protein biosynthesis |
| 89 | WP_003349508.1 | Cytoplasmic | Trx-like domain/YbbN | Promote assembly of proteins involved in and stress response and DNA replication | Protein folding |
| 90 | WP_044312661.1 | Cytoplasmic | Trx-like domain/YbbN | Promote assembly of proteins involved in and stress response and DNA replication | Protein folding |
| 91 | WP_057406977.1 | InnerMembrane | Peptidase C39 | Use thiol group as nucleophile from cysteine residues to hydrolyze peptide bond | Proteolysis |
| 92 | WP_057406961.1 | Extracellular | Thermolysin metallopeptidase, alpha-helical domain/Peptidase M4 | Secreted thermostable metalloprotease used to hydrolyze peptide | Proteolysis |
| 93 | WP_057406538.1 | Extracellular | Aconitase family | Reversible isomerization of citrate and isocitrate in TCA cycle, act as superoxide sensor and are involved in protection from oxidative stress | Response to oxidative stress |
| 94 | WP_002554851.1 | Cytoplasmic | Bacterioferritin/Ferritin-like diiron domain | Have ferroxidase activity which assist detoxification and protection from radical species | Response to oxidative stress |
| 95 | WP_003317095.1 | Cytoplasmic | Bacterioferritin/Ferritin-like diiron domain | Have ferroxidase activity which assist detoxification and protection from radical species | Response to oxidative stress |
| 96 | WP_057406103.1 | Cytoplasmic | Bacterioferritin/Ferritin-like diiron domain | Have ferroxidase activity which assist detoxification and protection from radical species | Response to oxidative stress |
| 97 | WP_003344123.1 | Periplasmic | Catalase | Reduce the free radical toxicity by converting hydrogen peroxide into water and oxygen | Response to oxidative stress |
| 98 | WP_057407284.1 | Cytoplasmic | Catalase | Reduce the free radical toxicity by converting hydrogen peroxide into water and oxygen | Response to oxidative stress |
| 99 | WP_003314356.1 | Cytoplasmic | Ferritin/DPS protein domain | Have ferroxidase activity which assist detoxification and protection from radical species | Response to oxidative stress |
| 100 | WP_002551467.1 | Cytoplasmic | Glutaredoxin | Alteration in redox state of the protein to maintain cell redox homeostasis | Response to oxidative stress |
| 101 | WP_002554852.1 | Cytoplasmic | Glutaredoxin | Alteration in redox state of the protein to maintain cell redox homeostasis | Response to oxidative stress |
| 102 | WP_044311912.1 | Cytoplasmic | Glutaredoxin | Alteration in redox state of the protein to maintain cell redox homeostasis | Response to oxidative stress |
| 103 | WP_003313497.1 | Periplasmic | Iron/manganese superoxide dismutases | Redox active enzyme to catalyse superoxide radicals conversion to molecular oxygen | Response to oxidative stress |
| 104 | WP_003395233.1 | Periplasmic | Iron/manganese superoxide dismutases | Redox active enzyme to catalyse superoxide radicals conversion to molecular oxygen | Response to oxidative stress |
| 105 | WP_003411784.1 | Cytoplasmic | tRNA N6-adenosine threonylcarbamoyltransferase, TsaD | Have DNA binding and endopeptidase activity and are involve in formation of threonylcarbamoyl group on adenosine in tRNA | RNA processing |
| 106 | WP_003424955.1 | Cytoplasmic | tRNA (Uracil-5-)-methyltransferase | Catalyses the formation of 5-methyl-uridine in tRNA for its maturation and stabilization | RNA processing |
| 107 | WP_003368536.1 | Cytoplasmic | tRNA-hydroxylase MiaE | Catalyses the posttranscriptional allylic hydroxylation of 2-methylthio-N-6-isopentenyl adenosine in tRNA | RNA processing |
| 108 | WP_057407245.1 | Cytoplasmic | Crp-type HTH domain | DNA binding protein involved in transcription regulation | Transcription regulation |
| 109 | WP_003316328.1 | Cytoplasmic | HDOD domain | Found in association with response regulator, GGDEF and EAL domain probably play role in regulation and signaling | Transcription regulation |
| 110 | WP_044311996.1 | Cytoplasmic | HDOD domain | Found in association with response regulator, GGDEF and EAL domain probably play role in regulation and signaling | Transcription regulation |
| 111 | WP_003395693.1 | Cytoplasmic | JmjC domain | Have demethylase and hydroxylases activity for transcription regulation | Transcription regulation |
| 112 | WP_003364071.1 | Cytoplasmic | NIF3 (NGG1p interacting factor 3) | NIF3 interacts with transcriptional coactivator NGG1P | Transcription regulation |
| 113 | WP_057405651.1 | Cytoplasmic | Pirin | Have quercetinase activity which require metal ion to regulate transcription | Transcription regulation |
| 114 | WP_057406965.1 | Cytoplasmic | Pirin | Have quercetinase activity which require metal ion to regulate transcription | Transcription regulation |
| 115 | WP_057407547.1 | Cytoplasmic | Pirin | Have quercetinase activity which require metal ion to regulate transcription | Transcription regulation |
| 116 | WP_024682945.1 | Cytoplasmic | TENA/THI-4/PQQC family | Enhancement of expression of some extracellular enzymes | Transcription regulation |
| 117 | WP_003316106.1 | Cytoplasmic | Rubredoxin | Redox active electron carrier protein mainly in anaerobes | Transport |
| 118 | WP_002553918.1 | InnerMembrane | ABC transporter | Integral membrane ATP-binding cassette transporters which uses ATP for import and export of wide range of substrates including metal ions | Transport |
| 119 | WP_002555631.1 | InnerMembrane | ABC transporter | Integral membrane ATP-binding cassette transporters which uses ATP for import and export of wide range of substrates including metal ions | Transport |
| 120 | WP_003313589.1 | InnerMembrane | ABC transporter | Integral membrane ATP-binding cassette transporters which uses ATP for import and export of wide range of substrates including metal ions | Transport |
| 121 | WP_003313770.1 | InnerMembrane | ABC transporter | Integral membrane ATP-binding cassette transporters which uses ATP for import and export of wide range of substrates including metal ions | Transport |
| 122 | WP_003316092.1 | InnerMembrane | ABC transporter | Integral membrane ATP-binding cassette transporters which uses ATP for import and export of wide range of substrates including metal ions | Transport |
| 123 | WP_003317225.1 | InnerMembrane | ABC transporter | Integral membrane ATP-binding cassette transporters which uses ATP for import and export of wide range of substrates including metal ions | Transport |
| 124 | WP_003317226.1 | InnerMembrane | ABC transporter | Integral membrane ATP-binding cassette transporters which uses ATP for import and export of wide range of substrates including metal ions | Transport |
| 125 | WP_003317352.1 | InnerMembrane | ABC transporter | Integral membrane ATP-binding cassette transporters which uses ATP for import and export of wide range of substrates including metal ions | Transport |
| 126 | WP_003317464.1 | InnerMembrane | ABC transporter | Integral membrane ATP-binding cassette transporters which uses ATP for import and export of wide range of substrates including metal ions | Transport |
| 127 | WP_003318317.1 | InnerMembrane | ABC transporter | Integral membrane ATP-binding cassette transporters which uses ATP for import and export of wide range of substrates including metal ions | Transport |
| 128 | WP_003318733.1 | InnerMembrane | ABC transporter | Integral membrane ATP-binding cassette transporters which uses ATP for import and export of wide range of substrates including metal ions | Transport |
| 129 | WP_003344757.1 | InnerMembrane | ABC transporter | Integral membrane ATP-binding cassette transporters which uses ATP for import and export of wide range of substrates including metal ions | Transport |
| 130 | WP_003349498.1 | InnerMembrane | ABC transporter | Integral membrane ATP-binding cassette transporters which uses ATP for import and export of wide range of substrates including metal ions | Transport |
| 131 | WP_003363626.1 | InnerMembrane | ABC transporter | Integral membrane ATP-binding cassette transporters which uses ATP for import and export of wide range of substrates including metal ions | Transport |
| 132 | WP_003371222.1 | InnerMembrane | ABC transporter | Integral membrane ATP-binding cassette transporters which uses ATP for import and export of wide range of substrates including metal ions | Transport |
| 133 | WP_003391874.1 | InnerMembrane | ABC transporter | Integral membrane ATP-binding cassette transporters which uses ATP for import and export of wide range of substrates including metal ions | Transport |
| 134 | WP_003394418.1 | InnerMembrane | ABC transporter | Integral membrane ATP-binding cassette transporters which uses ATP for import and export of wide range of substrates including metal ions | Transport |
| 135 | WP_003395611.1 | InnerMembrane | ABC transporter | Integral membrane ATP-binding cassette transporters which uses ATP for import and export of wide range of substrates including metal ions | Transport |
| 136 | WP_003396475.1 | InnerMembrane | ABC transporter | Integral membrane ATP-binding cassette transporters which uses ATP for import and export of wide range of substrates including metal ions | Transport |
| 137 | WP_003396818.1 | InnerMembrane | ABC transporter | Integral membrane ATP-binding cassette transporters which uses ATP for import and export of wide range of substrates including metal ions | Transport |
| 138 | WP_003399986.1 | InnerMembrane | ABC transporter | Integral membrane ATP-binding cassette transporters which uses ATP for import and export of wide range of substrates including metal ions | Transport |
| 139 | WP_003405691.1 | InnerMembrane | ABC transporter | Integral membrane ATP-binding cassette transporters which uses ATP for import and export of wide range of substrates including metal ions | Transport |
| 140 | WP_003406778.1 | InnerMembrane | ABC transporter | Integral membrane ATP-binding cassette transporters which uses ATP for import and export of wide range of substrates including metal ions | Transport |
| 141 | WP_003409725.1 | InnerMembrane | ABC transporter | Integral membrane ATP-binding cassette transporters which uses ATP for import and export of wide range of substrates including metal ions | Transport |
| 142 | WP_003410638.1 | InnerMembrane | ABC transporter | Integral membrane ATP-binding cassette transporters which uses ATP for import and export of wide range of substrates including metal ions | Transport |
| 143 | WP_003413043.1 | InnerMembrane | ABC transporter | Integral membrane ATP-binding cassette transporters which uses ATP for import and export of wide range of substrates including metal ions | Transport |
| 144 | WP_003420782.1 | InnerMembrane | ABC transporter | Integral membrane ATP-binding cassette transporters which uses ATP for import and export of wide range of substrates including metal ions | Transport |
| 145 | WP_003423922.1 | InnerMembrane | ABC transporter | Integral membrane ATP-binding cassette transporters which uses ATP for import and export of wide range of substrates including metal ions | Transport |
| 146 | WP_003424625.1 | InnerMembrane | ABC transporter | Integral membrane ATP-binding cassette transporters which uses ATP for import and export of wide range of substrates including metal ions | Transport |
| 147 | WP_003425787.1 | InnerMembrane | ABC transporter | Integral membrane ATP-binding cassette transporters which uses ATP for import and export of wide range of substrates including metal ions | Transport |
| 148 | WP_003427335.1 | InnerMembrane | ABC transporter | Integral membrane ATP-binding cassette transporters which uses ATP for import and export of wide range of substrates including metal ions | Transport |
| 149 | WP_003427838.1 | InnerMembrane | ABC transporter | Integral membrane ATP-binding cassette transporters which uses ATP for import and export of wide range of substrates including metal ions | Transport |
| 150 | WP_004394289.1 | InnerMembrane | ABC transporter | Integral membrane ATP-binding cassette transporters which uses ATP for import and export of wide range of substrates including metal ions | Transport |
| 151 | WP_004403512.1 | InnerMembrane | ABC transporter | Integral membrane ATP-binding cassette transporters which uses ATP for import and export of wide range of substrates including metal ions | Transport |
| 152 | WP_004415692.1 | InnerMembrane | ABC transporter | Integral membrane ATP-binding cassette transporters which uses ATP for import and export of wide range of substrates including metal ions | Transport |
| 153 | WP_004416899.1 | InnerMembrane | ABC transporter | Integral membrane ATP-binding cassette transporters which uses ATP for import and export of wide range of substrates including metal ions | Transport |
| 154 | WP_010413485.1 | InnerMembrane | ABC transporter | Integral membrane ATP-binding cassette transporters which uses ATP for import and export of wide range of substrates including metal ions | Transport |
| 155 | WP_010419706.1 | InnerMembrane | ABC transporter | Integral membrane ATP-binding cassette transporters which uses ATP for import and export of wide range of substrates including metal ions | Transport |
| 156 | WP_010424837.1 | InnerMembrane | ABC transporter | Integral membrane ATP-binding cassette transporters which uses ATP for import and export of wide range of substrates including metal ions | Transport |
| 157 | WP_010429566.1 | InnerMembrane | ABC transporter | Integral membrane ATP-binding cassette transporters which uses ATP for import and export of wide range of substrates including metal ions | Transport |
| 158 | WP_016566951.1 | InnerMembrane | ABC transporter | Integral membrane ATP-binding cassette transporters which uses ATP for import and export of wide range of substrates including metal ions | Transport |
| 159 | WP_016567612.1 | InnerMembrane | ABC transporter | Integral membrane ATP-binding cassette transporters which uses ATP for import and export of wide range of substrates including metal ions | Transport |
| 160 | WP_016568024.1 | InnerMembrane | ABC transporter | Integral membrane ATP-binding cassette transporters which uses ATP for import and export of wide range of substrates including metal ions | Transport |
| 161 | WP_016568489.1 | InnerMembrane | ABC transporter | Integral membrane ATP-binding cassette transporters which uses ATP for import and export of wide range of substrates including metal ions | Transport |
| 162 | WP_024661619.1 | InnerMembrane | ABC transporter | Integral membrane ATP-binding cassette transporters which uses ATP for import and export of wide range of substrates including metal ions | Transport |
| 163 | WP_024683414.1 | InnerMembrane | ABC transporter | Integral membrane ATP-binding cassette transporters which uses ATP for import and export of wide range of substrates including metal ions | Transport |
| 164 | WP_024959301.1 | InnerMembrane | ABC transporter | Integral membrane ATP-binding cassette transporters which uses ATP for import and export of wide range of substrates including metal ions | Transport |
| 165 | WP_024960521.1 | InnerMembrane | ABC transporter | Integral membrane ATP-binding cassette transporters which uses ATP for import and export of wide range of substrates including metal ions | Transport |
| 166 | WP_044311491.1 | InnerMembrane | ABC transporter | Integral membrane ATP-binding cassette transporters which uses ATP for import and export of wide range of substrates including metal ions | Transport |
| 167 | WP_044312272.1 | InnerMembrane | ABC transporter | Integral membrane ATP-binding cassette transporters which uses ATP for import and export of wide range of substrates including metal ions | Transport |
| 168 | WP_044312846.1 | InnerMembrane | ABC transporter | Integral membrane ATP-binding cassette transporters which uses ATP for import and export of wide range of substrates including metal ions | Transport |
| 169 | WP_044313343.1 | InnerMembrane | ABC transporter | Integral membrane ATP-binding cassette transporters which uses ATP for import and export of wide range of substrates including metal ions | Transport |
| 170 | WP_057405627.1 | InnerMembrane | ABC transporter | Integral membrane ATP-binding cassette transporters which uses ATP for import and export of wide range of substrates including metal ions | Transport |
| 171 | WP_057405633.1 | InnerMembrane | ABC transporter | Integral membrane ATP-binding cassette transporters which uses ATP for import and export of wide range of substrates including metal ions | Transport |
| 172 | WP_057405718.1 | InnerMembrane | ABC transporter | Integral membrane ATP-binding cassette transporters which uses ATP for import and export of wide range of substrates including metal ions | Transport |
| 173 | WP_057405772.1 | InnerMembrane | ABC transporter | Integral membrane ATP-binding cassette transporters which uses ATP for import and export of wide range of substrates including metal ions | Transport |
| 174 | WP_057405815.1 | InnerMembrane | ABC transporter | Integral membrane ATP-binding cassette transporters which uses ATP for import and export of wide range of substrates including metal ions | Transport |
| 175 | WP_057406029.1 | InnerMembrane | ABC transporter | Integral membrane ATP-binding cassette transporters which uses ATP for import and export of wide range of substrates including metal ions | Transport |
| 176 | WP_057406098.1 | InnerMembrane | ABC transporter | Integral membrane ATP-binding cassette transporters which uses ATP for import and export of wide range of substrates including metal ions | Transport |
| 177 | WP_057406145.1 | InnerMembrane | ABC transporter | Integral membrane ATP-binding cassette transporters which uses ATP for import and export of wide range of substrates including metal ions | Transport |
| 178 | WP_057406195.1 | InnerMembrane | ABC transporter | Integral membrane ATP-binding cassette transporters which uses ATP for import and export of wide range of substrates including metal ions | Transport |
| 179 | WP_057406196.1 | InnerMembrane | ABC transporter | Integral membrane ATP-binding cassette transporters which uses ATP for import and export of wide range of substrates including metal ions | Transport |
| 180 | WP_057406223.1 | InnerMembrane | ABC transporter | Integral membrane ATP-binding cassette transporters which uses ATP for import and export of wide range of substrates including metal ions | Transport |
| 181 | WP_057406345.1 | InnerMembrane | ABC transporter | Integral membrane ATP-binding cassette transporters which uses ATP for import and export of wide range of substrates including metal ions | Transport |
| 182 | WP_057406444.1 | InnerMembrane | ABC transporter | Integral membrane ATP-binding cassette transporters which uses ATP for import and export of wide range of substrates including metal ions | Transport |
| 183 | WP_057406537.1 | InnerMembrane | ABC transporter | Integral membrane ATP-binding cassette transporters which uses ATP for import and export of wide range of substrates including metal ions | Transport |
| 184 | WP_057406731.1 | InnerMembrane | ABC transporter | Integral membrane ATP-binding cassette transporters which uses ATP for import and export of wide range of substrates including metal ions | Transport |
| 185 | WP_057406813.1 | InnerMembrane | ABC transporter | Integral membrane ATP-binding cassette transporters which uses ATP for import and export of wide range of substrates including metal ions | Transport |
| 186 | WP_057406890.1 | InnerMembrane | ABC transporter | Integral membrane ATP-binding cassette transporters which uses ATP for import and export of wide range of substrates including metal ions | Transport |
| 187 | WP_057406900.1 | InnerMembrane | ABC transporter | Integral membrane ATP-binding cassette transporters which uses ATP for import and export of wide range of substrates including metal ions | Transport |
| 188 | WP_057406916.1 | InnerMembrane | ABC transporter | Integral membrane ATP-binding cassette transporters which uses ATP for import and export of wide range of substrates including metal ions | Transport |
| 189 | WP_057406926.1 | InnerMembrane | ABC transporter | Integral membrane ATP-binding cassette transporters which uses ATP for import and export of wide range of substrates including metal ions | Transport |
| 190 | WP_057406928.1 | InnerMembrane | ABC transporter | Integral membrane ATP-binding cassette transporters which uses ATP for import and export of wide range of substrates including metal ions | Transport |
| 191 | WP_057407014.1 | InnerMembrane | ABC transporter | Integral membrane ATP-binding cassette transporters which uses ATP for import and export of wide range of substrates including metal ions | Transport |
| 192 | WP_057407128.1 | InnerMembrane | ABC transporter | Integral membrane ATP-binding cassette transporters which uses ATP for import and export of wide range of substrates including metal ions | Transport |
| 193 | WP_057407220.1 | InnerMembrane | ABC transporter | Integral membrane ATP-binding cassette transporters which uses ATP for import and export of wide range of substrates including metal ions | Transport |
| 194 | WP_057407226.1 | InnerMembrane | ABC transporter | Integral membrane ATP-binding cassette transporters which uses ATP for import and export of wide range of substrates including metal ions | Transport |
| 195 | WP_057407405.1 | InnerMembrane | ABC transporter | Integral membrane ATP-binding cassette transporters which uses ATP for import and export of wide range of substrates including metal ions | Transport |
| 196 | WP_057407521.1 | InnerMembrane | ABC transporter | Integral membrane ATP-binding cassette transporters which uses ATP for import and export of wide range of substrates including metal ions | Transport |
| 197 | WP_057407551.1 | InnerMembrane | ABC transporter | Integral membrane ATP-binding cassette transporters which uses ATP for import and export of wide range of substrates including metal ions | Transport |
| 198 | WP_058877178.1 | InnerMembrane | ABC transporter | Integral membrane ATP-binding cassette transporters which uses ATP for import and export of wide range of substrates including metal ions | Transport |
| 199 | WP_058877187.1 | InnerMembrane | ABC transporter | Integral membrane ATP-binding cassette transporters which uses ATP for import and export of wide range of substrates including metal ions | Transport |
| 200 | WP_044312913.1 | Periplasmic | Adhesion lipoprotein | Act as receptors for transport, host cell attachment, colonisation and adhesion | Transport |
| 201 | WP_057406036.1 | Periplasmic | Adhesion lipoprotein | Act as receptors for transport, host cell attachment, colonisation and adhesion | Transport |
| 202 | WP_057406514.1 | Periplasmic | Bacterial extracellular solute-binding protein | Act as receptors for transport and signal transduction system | Transport |
| 203 | WP_032662671.1 | Periplasmic | Bacterial extracellular solute-binding proteins, family 5 | Act as receptors for transport and signal transduction system | Transport |
| 204 | WP_057405715.1 | Periplasmic | Bacterial extracellular solute-binding proteins, family 5 | Act as receptors for transport and signal transduction system | Transport |
| 205 | WP_057406194.1 | Periplasmic | Bacterial extracellular solute-binding proteins, family 5 | Act as receptors for transport and signal transduction system | Transport |
| 206 | WP_057406235.1 | Periplasmic | Bacterial extracellular solute-binding proteins, family 5 | Act as receptors for transport and signal transduction system | Transport |
| 207 | WP_057406522.1 | Periplasmic | Bacterial extracellular solute-binding proteins, family 5 | Act as receptors for transport and signal transduction system | Transport |
| 208 | WP_058877211.1 | Periplasmic | Bacterial extracellular solute-binding proteins, family 5 | Act as receptors for transport and signal transduction system | Transport |
| 209 | WP_024664231.1 | Cytoplasmic | Bacterial-like globin | Heme-containing protein function in oxygen binding and oxygen transport | Transport |
| 210 | WP_003341876.1 | InnerMembrane | Cytochrome C and Quinol oxidase polypeptide I | Catalyses the last step in electron transport chain i.e reduction of oxygen to water after obtaining the electrons from cytochrome c and transfer them to oxygen | Transport |
| 211 | WP_057405863.1 | Periplasmic | Cytochrome c, class IA/ IB | Catalyses the last step in electron transport chain i.e reduction of oxygen to water after obtaining the electrons from cytochrome c and transfer them to oxygen | Transport |
| 212 | WP_003343974.1 | InnerMembrane | NUBPL iron-transfer P-loop NTPase | ATPase contains iron-sulphur cluster assembling factor needed for export and biosynthesis of ribosomal subunits | Transport |
| 213 | WP_057405766.1 | Periplasmic | Periplasmic binding protein | Act as receptors for transport and signal transduction system | Transport |
| 214 | WP_057405965.1 | Periplasmic | Periplasmic binding protein | Act as receptors for transport and signal transduction system | Transport |
| 215 | WP_057406118.1 | Periplasmic | Periplasmic binding protein | Act as receptors for transport and signal transduction system | Transport |
| 216 | WP_057406158.1 | Periplasmic | Periplasmic binding protein | Act as receptors for transport and signal transduction system | Transport |
| 217 | WP_057407289.1 | Periplasmic | Periplasmic solute binding protein, ZnuA-like | Act as receptors for transport and signal transduction system | Transport |
| 218 | WP_044312813.1 | Cytoplasmic | Rieske [2Fe-2S] domain | Catalyses oxidation reduction reaction in electron transport chain | Transport |
| 219 | WP_024661472.1 | OuterMembrane | TonB-dependent receptor, plug domain | Act as receptors for the active transport of Fe-siderophore complexes and other substrates | Transport |
| 220 | WP_024684592.1 | OuterMembrane | TonB-dependent receptor, plug domain | Act as receptors for the active transport of Fe-siderophore complexes and other substrates | Transport |
| 221 | WP_044312375.1 | OuterMembrane | TonB-dependent receptor, plug domain | Act as receptors for the active transport of Fe-siderophore complexes and other substrates | Transport |
| 222 | WP_057405877.1 | OuterMembrane | TonB-dependent receptor, plug domain | Act as receptors for the active transport of Fe-siderophore complexes and other substrates | Transport |
| 223 | WP_057406030.1 | OuterMembrane | TonB-dependent receptor, plug domain | Act as receptors for the active transport of Fe-siderophore complexes and other substrates | Transport |
| 224 | WP_057406095.1 | OuterMembrane | TonB-dependent receptor, plug domain | Act as receptors for the active transport of Fe-siderophore complexes and other substrates | Transport |
| 225 | WP_057406164.1 | OuterMembrane | TonB-dependent receptor, plug domain | Act as receptors for the active transport of Fe-siderophore complexes and other substrates | Transport |
| 226 | WP_057406431.1 | OuterMembrane | TonB-dependent receptor, plug domain | Act as receptors for the active transport of Fe-siderophore complexes and other substrates | Transport |
| 227 | WP_057406756.1 | OuterMembrane | TonB-dependent receptor, plug domain | Act as receptors for the active transport of Fe-siderophore complexes and other substrates | Transport |
| 228 | WP_057407139.1 | OuterMembrane | TonB-dependent receptor, plug domain | Act as receptors for the active transport of Fe-siderophore complexes and other substrates | Transport |
| 229 | WP_057407153.1 | OuterMembrane | TonB-dependent receptor, plug domain | Act as receptors for the active transport of Fe-siderophore complexes and other substrates | Transport |
| 230 | WP_057407445.1 | OuterMembrane | TonB-dependent receptor, plug domain | Act as receptors for the active transport of Fe-siderophore complexes and other substrates | Transport |
| 231 | WP_057407519.1 | OuterMembrane | TonB-dependent receptor, plug domain | Act as receptors for the active transport of Fe-siderophore complexes and other substrates | Transport |
| 232 | WP_057407617.1 | OuterMembrane | TonB-dependent receptor, plug domain | Act as receptors for the active transport of Fe-siderophore complexes and other substrates | Transport |

**Supplementary Table S2 : The details of functionally enriched GO network of iron binding proteins**

| **S. No.** | **GOID** | **GOTerm** | **GOGroups** | **Nr. Genes** | **Degree** |
| --- | --- | --- | --- | --- | --- |
| 1 | GO:0006810 | transport | Group01, Group04, Group10 | 59 | 66 |
| 2 | GO:0055085 | transmembrane transport | Group01, Group10 | 29 | 43 |
| 3 | GO:0006811 | ion transport | Group03, Group10 | 19 | 40 |
| 4 | GO:0071702 | organic substance transport | Group01, Group04 | 30 | 38 |
| 5 | GO:0071705 | nitrogen compound transport | Group04 | 28 | 34 |
| 6 | GO:0006820 | anion transport | Group10 | 13 | 34 |
| 7 | GO:0034220 | ion transmembrane transport | Group10 | 13 | 34 |
| 8 | GO:0098656 | anion transmembrane transport | Group10 | 12 | 33 |
| 9 | GO:0098660 | inorganic ion transmembrane transport | Group10 | 13 | 33 |
| 10 | GO:0098661 | inorganic anion transmembrane transport | Group10 | 12 | 33 |
| 11 | GO:0099131 | ATP hydrolysis coupled ion transmembrane transport | Group10 | 12 | 32 |
| 12 | GO:0015698 | inorganic anion transport | Group10 | 12 | 31 |
| 13 | GO:0090662 | ATP hydrolysis coupled transmembrane transport | Group10 | 12 | 31 |
| 14 | GO:0099133 | ATP hydrolysis coupled anion transmembrane transport | Group10 | 12 | 31 |
| 15 | GO:0046942 | carboxylic acid transport | Group10 | 9 | 29 |
| 16 | GO:0006520 | cellular amino acid metabolic process | Group06, Group08 | 10 | 28 |
| 17 | GO:0006865 | amino acid transport | Group10 | 9 | 28 |
| 18 | GO:0015711 | organic anion transport | Group10 | 9 | 28 |
| 19 | GO:0015849 | organic acid transport | Group10 | 9 | 28 |
| 20 | GO:1905039 | carboxylic acid transmembrane transport | Group10 | 8 | 28 |
| 21 | GO:1901605 | alpha-amino acid metabolic process | Group06, Group08 | 9 | 27 |
| 22 | GO:1903825 | organic acid transmembrane transport | Group10 | 8 | 27 |
| 23 | GO:0003333 | amino acid transmembrane transport | Group10 | 8 | 26 |
| 24 | GO:0019752 | carboxylic acid metabolic process | Group06, Group08 | 14 | 25 |
| 25 | GO:0019725 | cellular homeostasis | Group09 | 8 | 25 |
| 26 | GO:0042592 | homeostatic process | Group09 | 8 | 24 |
| 27 | GO:0006082 | organic acid metabolic process | Group06, Group08 | 14 | 23 |
| 28 | GO:0043436 | oxoacid metabolic process | Group06, Group08 | 14 | 23 |
| 29 | GO:0050801 | ion homeostasis | Group09 | 3 | 22 |
| 30 | GO:0055065 | metal ion homeostasis | Group09 | 3 | 22 |
| 31 | GO:0015833 | peptide transport | Group04 | 16 | 21 |
| 32 | GO:0042886 | amide transport | Group04 | 16 | 21 |
| 33 | GO:0006873 | cellular ion homeostasis | Group09 | 3 | 21 |
| 34 | GO:0006875 | cellular metal ion homeostasis | Group09 | 3 | 21 |
| 35 | GO:0030003 | cellular cation homeostasis | Group09 | 3 | 21 |
| 36 | GO:0046916 | cellular transition metal ion homeostasis | Group09 | 3 | 21 |
| 37 | GO:0048878 | chemical homeostasis | Group09 | 3 | 21 |
| 38 | GO:0055080 | cation homeostasis | Group09 | 3 | 21 |
| 39 | GO:0055082 | cellular chemical homeostasis | Group09 | 3 | 21 |
| 40 | GO:0030001 | metal ion transport | Group03, Group09 | 4 | 20 |
| 41 | GO:0055076 | transition metal ion homeostasis | Group09 | 3 | 20 |
| 42 | GO:0098771 | inorganic ion homeostasis | Group09 | 3 | 20 |
| 43 | GO:0090407 | organophosphate biosynthetic process | Group07 | 5 | 19 |
| 44 | GO:0006879 | cellular iron ion homeostasis | Group09 | 3 | 19 |
| 45 | GO:0000041 | transition metal ion transport | Group03, Group09 | 3 | 18 |
| 46 | GO:0009124 | nucleoside monophosphate biosynthetic process | Group07 | 3 | 18 |
| 47 | GO:0009260 | ribonucleotide biosynthetic process | Group07 | 3 | 18 |
| 48 | GO:1901293 | nucleoside phosphate biosynthetic process | Group07 | 3 | 18 |
| 49 | GO:0009063 | cellular amino acid catabolic process | Group08 | 3 | 18 |
| 50 | GO:0016054 | organic acid catabolic process | Group08 | 3 | 18 |
| 51 | GO:0009123 | nucleoside monophosphate metabolic process | Group07 | 3 | 17 |
| 52 | GO:0009156 | ribonucleoside monophosphate biosynthetic process | Group07 | 3 | 17 |
| 53 | GO:0009161 | ribonucleoside monophosphate metabolic process | Group07 | 3 | 17 |
| 54 | GO:0009165 | nucleotide biosynthetic process | Group07 | 3 | 17 |
| 55 | GO:0046390 | ribose phosphate biosynthetic process | Group07 | 3 | 17 |
| 56 | GO:0044248 | cellular catabolic process | Group08 | 6 | 17 |
| 57 | GO:0046395 | carboxylic acid catabolic process | Group08 | 3 | 17 |
| 58 | GO:0009116 | nucleoside metabolic process | Group07 | 3 | 16 |
| 59 | GO:0009259 | ribonucleotide metabolic process | Group07 | 3 | 16 |
| 60 | GO:1901657 | glycosyl compound metabolic process | Group07 | 3 | 16 |
| 61 | GO:0019439 | aromatic compound catabolic process | Group08 | 5 | 16 |
| 62 | GO:1901606 | alpha-amino acid catabolic process | Group08 | 3 | 16 |
| 63 | GO:0072521 | purine-containing compound metabolic process | Group07 | 3 | 15 |
| 64 | GO:0044282 | small molecule catabolic process | Group08 | 3 | 15 |
| 65 | GO:1901565 | organonitrogen compound catabolic process | Group08 | 3 | 15 |
| 66 | GO:0009072 | aromatic amino acid family metabolic process | Group08 | 3 | 14 |
| 67 | GO:1901361 | organic cyclic compound catabolic process | Group08 | 3 | 14 |
| 68 | GO:0006812 | cation transport | Group03, Group09, Group10 | 8 | 13 |
| 69 | GO:0009064 | glutamine family amino acid metabolic process | Group06, Group08 | 5 | 12 |
| 70 | GO:1901607 | alpha-amino acid biosynthetic process | Group06, Group08 | 5 | 12 |
| 71 | GO:1901678 | iron coordination entity transport | Group02 | 7 | 11 |
| 72 | GO:0098754 | detoxification | Group05 | 3 | 11 |
| 73 | GO:1990748 | cellular detoxification | Group05 | 3 | 11 |
| 74 | GO:0015688 | iron chelate transport | Group02 | 6 | 10 |
| 75 | GO:0009636 | response to toxic substance | Group05 | 3 | 10 |
| 76 | GO:0033554 | cellular response to stress | Group05 | 4 | 10 |
| 77 | GO:0098869 | cellular oxidant detoxification | Group05 | 3 | 10 |
| 78 | GO:0015891 | siderophore transport | Group02 | 6 | 9 |
| 79 | GO:0006979 | response to oxidative stress | Group05 | 3 | 9 |
| 80 | GO:0072593 | reactive oxygen species metabolic process | Group05 | 3 | 9 |
| 81 | GO:0032787 | monocarboxylic acid metabolic process | Group06 | 4 | 8 |
| 82 | GO:0045454 | cell redox homeostasis | Group09 | 5 | 8 |
| 83 | GO:0006790 | sulfur compound metabolic process | Group00 | 3 | 3 |

**Supplementary Table S3: Secreted iron binding proteins**

| **S. No.** | **Sequence Id** | **Signal peptide** | **Tat motif** | **Position** | **Sec-score** | **Transmembrane helix** |
| --- | --- | --- | --- | --- | --- | --- |
| 1 | WP_003416184.1 | - | - | - | 0.51855 | Nil |
| 2 | WP_024661472.1 | Y | - | 26 | - | Nil |
| 3 | WP_024684592.1 | Y | - | 28 | - | Nil |
| 4 | WP_032662671.1 | Y | - | 24 | - | Nil |
| 5 | WP_044312375.1 | Y | - | 29 | - | Nil |
| 6 | WP_044312913.1 | Y | - | 25 | - | Nil |
| 7 | WP_057405715.1 | Y | - | 24 | - | Nil |
| 8 | WP_057405766.1 | Y | - | 25 | - | Nil |
| 9 | WP_057405877.1 | Y | - | 24 | - | Nil |
| 10 | WP_057405965.1 | Y | - | 22 | - | Nil |
| 11 | WP_057406030.1 | - | - | - | 0.89934 | Nil |
| 12 | WP_057406036.1 | Y | Y | 30 (Tat motif starting at position 7) | - | Nil |
| 13 | WP_057406095.1 | Y | - | 27 | - | Nil |
| 14 | WP_057406118.1 | Y | - | 22 | - | Nil |
| 15 | WP_057406164.1 | Y | - | 34 | - | Nil |
| 16 | WP_057406194.1 | Y | - | 23 | - | Nil |
| 17 | WP_057406235.1 | Y | - | 25 | - | Nil |
| 18 | WP_057406431.1 | - | - | - | 0.90697 | Nil |
| 19 | WP_057406514.1 | Y | - | 23 | - | Nil |
| 20 | WP_057406522.1 | Y | - | 22 | - | Nil |
| 21 | WP_057406538.1 | - | - | - | 0.9366 | Nil |
| 22 | WP_057406756.1 | Y | - | 32 | - | Nil |
| 23 | WP_057406961.1 | - | - | - | 0.90378 | Nil |
| 24 | WP_057407139.1 | Y | - | 21 | - | Nil |
| 25 | WP_057407153.1 | - | - | - | 0.94139 | Nil |
| 26 | WP_057407445.1 | - | - | - | 0.95128 | Nil |
| 27 | WP_057407519.1 | - | - | - | 0.94719 | Nil |
| 28 | WP_057407617.1 | - | - | - | 0.94485 | Nil |
| 29 | WP_058877211.1 | Y | - | 25 | - | Nil |

**Supplementary Table S4: List of interacting residues in secreted iron binding proteins**

| **S. No.** | **Sequence Id** | **Template** | **Interacting residues** |
| --- | --- | --- | --- |
| 1 | WP_024661472.1 | 3QLB | EFE, ILE122, PHE402 |
| 2 | WP_024684592.1 | 2IAH | Pve1, ARG130, ILE161, THR723, ASP724 |
| 3 | WP_044312375.1 | 3QLB | EFE, TYR108 |
| 4 | WP_057405877.1 | 2W78 | PVE, 182PHE,186ARG, 770ALA |
| 5 | WP_057406095.1 | 3QLB | EFE, HIS112 |
| 6 | WP_057406164.1 | 2IAH | PVE, GLN203 |
| 7 | WP_057406756.1 | 1QFF | FCI, ALA198, TYR418 |
| 8 | WP_057407139.1 | 3QLB | EFE, GLY111 |
| 9 | WP_057406030.1 | 2IAH | Pve1, ARG192 |
| 10 | WP_057406431.1 | 1PO3 | FLC1, FLC2, ARG407, ARG461, ARG422 |
| 11 | WP_057407153.1 | 2IAH | PVE, SER120, TRP698, GLY699 |
| 12 | WP_057407617.1 | 2W78 | PVE, GLY111, ASP116, ASN733, TYR734 |
| 13 | WP_057407519.1 | 1PO3 | FLC1, FLC2, THR484, SER519, PHE572 |
| 14 | WP_057407445.1 | 3QLB | EFE, 411TYR |
| 15 | WP_057405766.1 | 4FIL | OUE, HIS198, TYR200 |
| 16 | WP_057405965.1 | 2R7A | GLY84 |
| 17 | WP_057406118.1 | 2R7A | 92ASP |
| 18 | WP_057406514.1 | 3F11 | ILE33, ASP34, TYR164, TYR220, TYR221 |
| 19 | WP_032662671.1 | 4OES | EDT, ALA430 |
| 20 | WP_057405715.1 | 4OES | EDT, VAL162, TRP410 |
| 21 | WP_057406194.1 | 4OES | EDT, TYR157, TRP416 |
| 22 | WP_057406235.1 | 4I9D | L4D, TYR44, THR137 |
| 23 | WP_057406522.1 | 4I9D | L4D, TRP426, ARG519 |
| 24 | WP_058877211.1 | 4I9D | L4D, PHE179 |
| 25 | WP_044312913.1 | 3HH8 | HIS61, HIS126, HIS192, GLU267 |
| 26 | WP_057406036.1 | 3HH8 | HIS70, HIS140, HIS206, HIS279 |
| 27 | WP_003416184.1 | 3T67 | Caq1, HIS163 TYR109, TYR148, HIS161 |
| 28 | WP_057406538.1 | 1NIT | CYS6, CYS71, CYS75 |
| 29 | WP_057406961.1 | 1LNB | HIS176, HIS180, GLU200 |

**Supplementary Table S5 : Subcellular localization and Functional domain analysis of zinc binding proteins**

| **S. No.** | **Sequence ID** | **Subcellular localization** | **Functional domain/family** | **Description** | **Broad class** |
| --- | --- | --- | --- | --- | --- |
| 1 | WP_057407431.1 | Periplasmic | Beta-lactamase | Hydrolyze the amide bond in beta-lactam compounds | Antimicrobial resistance |
| 2 | WP_057407492.1 | Cytoplasmic | Metallo-beta-lactamase | Hydrolyze the amide bond in beta-lactam compounds | Antimicrobial resistance |
| 3 | WP_003316096.1 | OuterMembrane | Peptidase family M23 | Interfering in biosynthesis and metabolism of cell wall peptidoglycans | Antimicrobial resistance |
| 4 | WP_003422785.1 | OuterMembrane | Peptidase family M23 | Interfering in biosynthesis and metabolism of cell wall peptidoglycans | Antimicrobial resistance |
| 5 | WP_057405551.1 | OuterMembrane | Peptidase family M23 | Interfering in biosynthesis and metabolism of cell wall peptidoglycans | Antimicrobial resistance |
| 6 | WP_057406372.1 | OuterMembrane | Peptidase family M23 | Interfering in biosynthesis and metabolism of cell wall peptidoglycans | Antimicrobial resistance |
| 7 | WP_057406661.1 | InnerMembrane | Transglycosylase | Cell-wall metabolism and maintaining the integrity of the peptidoglycan layers | Antimicrobial resistance |
| 8 | WP_057406063.1 | Cytoplasmic | Diguanylate cyclase, GGDEF domain | Catalyze synthesis or hydrolysis of cyclic diguanylate | Cell signaling |
| 9 | WP_016567028.1 | Cytoplasmic | EAL domain | Stimulate degradation of cyclic di-GMP (second messenger) | Cell signaling |
| 10 | WP_057406413.1 | Cytoplasmic | EAL domain | Stimulate degradation of cyclic di-GMP (second messenger) | Cell signaling |
| 11 | WP_003314166.1 | Cytoplasmic | Inositol monophosphatase family | Biosynthesis of inositol phosphates and inositol phospholipids which are involved in signal transduction | Cell signaling |
| 12 | WP_057406953.1 | Extracellular | Leucine rich repeat | Integrators of pattern recongination and signaling | Cell signaling |
| 13 | WP_002555108.1 | Cytoplasmic | MreB/Mbl protein | Act as scaffolds, guiding the localization and activity of key peptidoglycan synthesizing proteins during cell elongation | Cell signaling |
| 14 | WP_002551738.1 | Cytoplasmic | Response regulator receiver domain | Act as phosphorylation-activated switches to affect a cellular response | Cell signaling |
| 15 | WP_002555882.1 | Cytoplasmic | Response regulator receiver domain | Act as phosphorylation-activated switches to affect a cellular response | Cell signaling |
| 16 | WP_003314580.1 | Cytoplasmic | Response regulator receiver domain | Act as phosphorylation-activated switches to affect a cellular response | Cell signaling |
| 17 | WP_003315433.1 | Cytoplasmic | Response regulator receiver domain | Act as phosphorylation-activated switches to affect a cellular response | Cell signaling |
| 18 | WP_003316095.1 | Cytoplasmic | Response regulator receiver domain | Act as phosphorylation-activated switches to affect a cellular response | Cell signaling |
| 19 | WP_003316827.1 | Cytoplasmic | Response regulator receiver domain | Act as phosphorylation-activated switches to affect a cellular response | Cell signaling |
| 20 | WP_003341820.1 | Cytoplasmic | Response regulator receiver domain | Act as phosphorylation-activated switches to affect a cellular response | Cell signaling |
| 21 | WP_003363740.1 | Cytoplasmic | Response regulator receiver domain | Act as phosphorylation-activated switches to affect a cellular response | Cell signaling |
| 22 | WP_003393295.1 | Cytoplasmic | Response regulator receiver domain | Act as phosphorylation-activated switches to affect a cellular response | Cell signaling |
| 23 | WP_003393712.1 | Cytoplasmic | Response regulator receiver domain | Act as phosphorylation-activated switches to affect a cellular response | Cell signaling |
| 24 | WP_003394668.1 | Cytoplasmic | Response regulator receiver domain | Act as phosphorylation-activated switches to affect a cellular response | Cell signaling |
| 25 | WP_003406975.1 | Cytoplasmic | Response regulator receiver domain | Act as phosphorylation-activated switches to affect a cellular response | Cell signaling |
| 26 | WP_003412541.1 | Cytoplasmic | Response regulator receiver domain | Act as phosphorylation-activated switches to affect a cellular response | Cell signaling |
| 27 | WP_003421813.1 | Cytoplasmic | Response regulator receiver domain | Act as phosphorylation-activated switches to affect a cellular response | Cell signaling |
| 28 | WP_010426439.1 | Cytoplasmic | Response regulator receiver domain | Act as phosphorylation-activated switches to affect a cellular response | Cell signaling |
| 29 | WP_044312298.1 | Cytoplasmic | Response regulator receiver domain | Act as phosphorylation-activated switches to affect a cellular response | Cell signaling |
| 30 | WP_004406399.1 | InnerMembrane | SRP54-type protein, GTPase domain | SRP-dependent co-translational protein targeting to plasma membrane | Cell signaling |
| 31 | WP_003315068.1 | Cytoplasmic | PPM-type phosphatase domain | Catalyzes the dephosphorylation of phosphoserine and phosphothreonine containing protein | Cell signaling |
| 32 | WP_057406894.1 | Cytoplasmic | AP2Ec superfamily | Create a nick in the phosphodiester backbone of the AP site | DNA repair |
| 33 | WP_057406308.1 | Cytoplasmic | DNA methylase, C-5 cytosine-specific, active site | Methylate the C-5 carbon of cytosines in DNA | DNA repair |
| 34 | WP_057406307.1 | Cytoplasmic | DNA mismatch endonuclease Vsr | VSR recognises a TG mismatched base pair which is generated after spontaneous deamination of methylated cytosines and cleaves the phosphate backbone on the 5' side of the thymine. | DNA repair |
| 35 | WP_057406603.1 | Cytoplasmic | HhH-GPD domain | Helix-hairpin-helix anfd Gly/Pro rich loop followed by a conserved aspartate associated with base excision repair | DNA repair |
| 36 | WP_057406193.1 | Cytoplasmic | Metallo-dependent phosphatase-like | Hydrolyse phosphomonoesters, phosphodiesters and phophotriesters in a metal dependent manner | DNA repair |
| 37 | WP_057407268.1 | Cytoplasmic | Metallo-dependent phosphatase-like | Hydrolyse phosphomonoesters, phosphodiesters and phophotriesters in a metal dependent manner | DNA repair |
| 38 | WP_057407599.1 | Cytoplasmic | Metallo-dependent phosphatase-like | Hydrolyse phosphomonoesters, phosphodiesters and phophotriesters in a metal dependent manner | DNA repair |
| 39 | WP_003314590.1 | Cytoplasmic | Methyladenine glycosylase | Catalyses the release of 3-methylated adenine and to some extent, guanosine bases from DNA by hydrolysis of deoxyribose N-glycosidic bond | DNA repair |
| 40 | WP_003401445.1 | Cytoplasmic | Methyladenine glycosylase | Catalyses the release of 3-methylated adenine and to some extent, guanosine bases from DNA by hydrolysis of deoxyribose N-glycosidic bond | DNA repair |
| 41 | WP_057406616.1 | Cytoplasmic | RecQ_Zn_bind | ATP-dependent molecular motors that unwind double-stranded DNA and catalyses critical genome maintenance reactions | DNA repair |
| 42 | WP_057406317.1 | Cytoplasmic | ATP-dependent DNA helicase RecQ, zinc-binding domain | ATP-dependent molecular motors that unwind double-stranded DNA and catalyses critical genome maintenance reactions | DNA repair |
| 43 | WP_003317093.1 | Periplasmic | Single-strand binding protein family | SSB proteins binds to ssDNA and to an array of partner proteins to recruit them to their sites of action during DNA metabolism | DNA repair |
| 44 | WP_003318499.1 | Cytoplasmic | TatD related DNase | 3'-5' exonuclease that prefers to digest single-stranded DNA during DNA repair | DNA repair |
| 45 | WP_003423655.1 | Cytoplasmic | TatD related DNase | 3'-5' exonuclease that prefers to digest single-stranded DNA during DNA repair | DNA repair |
| 46 | WP_057407141.1 | Cytoplasmic | TatD related DNase | 3'-5' exonuclease that prefers to digest single-stranded DNA during DNA repair | DNA repair |
| 47 | WP_024649959.1 | Cytoplasmic | Ultra-violet resistance protein B | Conveys mutational resistance against UV light | DNA repair |
| 48 | WP_044311542.1 | Cytoplasmic | AP endonuclease | Create a nick in the phosphodiester backbone of the AP site | DNA repair |
| 49 | WP_003340175.1 | Cytoplasmic | DNA gyrase B | Modulate DNA topology by an ATP-dependent manner | DNA replication |
| 50 | WP_003342973.1 | Cytoplasmic | DNA gyrase B | Modulate DNA topology by an ATP-dependent manner | DNA replication |
| 51 | WP_057406508.1 | Cytoplasmic | DNA polymerase family A | Addition of nucleotide triphosphate residues to of growing DNA chain | DNA replication |
| 52 | WP_057407035.1 | Cytoplasmic | DNA polymerase family B | Addition of nucleotide triphosphate residues to growing DNA chain | DNA replication |
| 53 | WP_003368796.1 | Cytoplasmic | DNA polymerase III, delta subunit | Addition of nucleotide triphosphate residues to growing DNA chain | DNA replication |
| 54 | WP_016568498.1 | Cytoplasmic | DNA topoisomerase I, type IA | DNA topological change | DNA replication |
| 55 | WP_044311497.1 | Cytoplasmic | DNA topoisomerase III | DNA topological change | DNA replication |
| 56 | WP_057405558.1 | Cytoplasmic | 2-C-methyl-D-erythritol 4-phosphate cytidylyltransferase | Isoprenoid precursors biosynthesis | Metabolic process |
| 57 | WP_003421165.1 | Cytoplasmic | 3,4-dihydroxy-2-butanone 4-phosphate synthase | Biosynthesis of precursors for riboflavin | Metabolic process |
| 58 | WP_057407043.1 | Cytoplasmic | 3,4-dihydroxy-2-butanone 4-phosphate synthase | Biosynthesis of precursors for riboflavin | Metabolic process |
| 59 | WP_044313221.1 | Cytoplasmic | 3-hydroxyacyl-CoA dehydrogenase, NAD binding domain | Catalyses reduction of 3-hydroxyacyl-CoA to 3-oxoacyl-CoA and fatty acid metabolism | Metabolic process |
| 60 | WP_057406909.1 | Cytoplasmic | 3-hydroxyacyl-CoA dehydrogenase, NAD binding domain | Catalyses reduction of 3-hydroxyacyl-CoA to 3-oxoacyl-CoA and fatty acid metabolism | Metabolic process |
| 61 | WP_003364825.1 | Cytoplasmic | Acetyl co-enzyme A carboxylase carboxyltransferase alpha subunit | Carboxylation of acetyl-CoA to malonyl-CoA which is first step in biosynthesis of long chain fatty acids | Metabolic process |
| 62 | WP_003318645.1 | Cytoplasmic | Adenosine/AMP deaminase | Hydrolytic deamination of adenosine to inosine during purine metabolism | Metabolic process |
| 63 | WP_003314266.1 | Cytoplasmic | Adenylate kinase | Nucleotide phosphoryl exchange reaction | Metabolic process |
| 64 | WP_003418808.1 | Cytoplasmic | Adenylylsulphate kinase | Phosphorylation of adenylsulphate to 3-phosphoadenylsulphate | Metabolic process |
| 65 | WP_044312465.1 | Cytoplasmic | Aldo/keto reductase family | Reduction of carbonyl substrates | Metabolic process |
| 66 | WP_057407114.1 | Cytoplasmic | Aldo/keto reductase family | Reduction of carbonyl substrates | Metabolic process |
| 67 | WP_057407156.1 | Cytoplasmic | Aldo/keto reductase family | Reduction of carbonyl substrates | Metabolic process |
| 68 | WP_044313346.1 | Cytoplasmic | Aldolase_II | Condensation and cleavage reactions during gluconeogenesis and glycolysis | Metabolic process |
| 69 | WP_057406386.1 | Cytoplasmic | Alpha/beta hydrolase fold | Hydrolytic enzyme with diverse catalytic functions | Metabolic process |
| 70 | WP_003314686.1 | Cytoplasmic | Aminotransferase class I and II | Have pyridoxal phosphate binding and transaminase activity which are involve in various biosynthetic processes | Metabolic process |
| 71 | WP_003392640.1 | Cytoplasmic | Aminotransferase class I and II | Have pyridoxal phosphate binding and transaminase activity which are involve in various biosynthetic processes | Metabolic process |
| 72 | WP_044313105.1 | Cytoplasmic | Aminotransferase class I and II | Have pyridoxal phosphate binding and transaminase activity which are involve in various biosynthetic processes | Metabolic process |
| 73 | WP_057405566.1 | Cytoplasmic | Aminotransferase class I and II | Have pyridoxal phosphate binding and transaminase activity which are involve in various biosynthetic processes | Metabolic process |
| 74 | WP_057406601.1 | Cytoplasmic | Aminotransferase class I and II | Have pyridoxal phosphate binding and transaminase activity which are involve in various biosynthetic processes | Metabolic process |
| 75 | WP_003316561.1 | Cytoplasmic | ATP-grasp fold, succinyl-CoA synthetase-type | ATP dependent ligation of carboxylate containing molecule to amino or thiol containing molecule | Metabolic process |
| 76 | WP_003314213.1 | Cytoplasmic | Bacterial transferase hexapeptide (six repeats) | Tandem repeats of a [LIV]-G-X(4) hexapeptide with transferase activity | Metabolic process |
| 77 | WP_003317836.1 | Cytoplasmic | Bacterial transferase hexapeptide (six repeats) | Tandem repeats of a [LIV]-G-X(4) hexapeptide with transferase activity | Metabolic process |
| 78 | WP_044313466.1 | Cytoplasmic | Bacterial transferase hexapeptide (six repeats) | Tandem repeats of a [LIV]-G-X(4) hexapeptide with transferase activity | Metabolic process |
| 79 | WP_003432826.1 | Cytoplasmic | Biotin/lipoyl attachment domain | Have conserved lysine residue that either bind biotin or lipoic acid and have catalytic role in carboxyl transfer reactions | Metabolic process |
| 80 | WP_003345939.1 | Cytoplasmic | Carbonic anhydrase | Reversible hydration of carbon dioxide | Metabolic process |
| 81 | WP_004409041.1 | Cytoplasmic | Carbonic anhydrase | Reversible hydration of carbon dioxide | Metabolic process |
| 82 | WP_003318491.1 | Cytoplasmic | Carboxyl transferase domain | Catalytic activity for transcarboxylation reactions | Metabolic process |
| 83 | WP_003425332.1 | Cytoplasmic | CobW/HypB/UreG, nucleotide-binding domain | Cobalamin biosynthesis, hydrogenase expression, activation of urease proteins | Metabolic process |
| 84 | WP_044312185.1 | Cytoplasmic | CobW/HypB/UreG, nucleotide-binding domain | Cobalamin biosynthesis, hydrogenase expression, activation of urease proteins | Metabolic process |
| 85 | WP_057405676.1 | Cytoplasmic | CobW/HypB/UreG, nucleotide-binding domain | Cobalamin biosynthesis, hydrogenase expression, activation of urease proteins | Metabolic process |
| 86 | WP_003393272.1 | Cytoplasmic | Cytidine and deoxycytidylate deaminase zinc-binding region | Hydrolysis of cytidine into uridine and ammonia by cytidine deaminase and dCMP hydrolysis to dUMP by deoxycytidylate deaminase | Metabolic process |
| 87 | WP_003316710.1 | Cytoplasmic | DEAD/DEAH box helicase | Unwinding of nucleic acids and RNA metabolism | Metabolic process |
| 88 | WP_003340788.1 | Cytoplasmic | DEAD/DEAH box helicase | Unwinding of nucleic acids and RNA metabolism | Metabolic process |
| 89 | WP_003365984.1 | Cytoplasmic | DEAD/DEAH box helicase | Unwinding of nucleic acids and RNA metabolism | Metabolic process |
| 90 | WP_003402477.1 | Cytoplasmic | DEAD/DEAH box helicase | Unwinding of nucleic acids and RNA metabolism | Metabolic process |
| 91 | WP_003317357.1 | Cytoplasmic | Delta-aminolevulinic acid dehydratase | Functions in tetrapyrrole biosynthesis pathways | Metabolic process |
| 92 | WP_004410237.1 | Cytoplasmic | DeoC/LacD family aldolase | Nucleotide metabolism and tragatose-6-phosphate pathway | Metabolic process |
| 93 | WP_057406660.1 | Cytoplasmic | DeoC/LacD family aldolase | Nucleotide metabolism and tragatose-6-phosphate pathway | Metabolic process |
| 94 | WP_003339565.1 | Cytoplasmic | Enolase, C-terminal TIM barrel domain | Involve in glycolytic and gluconeogenesis pathways, catalyses reversible dehydration of 2-phospho-D-glycerate to phosphoenolpyruvate. | Metabolic process |
| 95 | WP_003315368.1 | Cytoplasmic | Enoyl-CoA hydratase/isomerase | Hydration of 2-trans-enoyl-CoA to 3-hyroxyacyl-CoA | Metabolic process |
| 96 | WP_003401024.1 | Cytoplasmic | Enoyl-CoA hydratase/isomerase | Hydration of 2-trans-enoyl-CoA to 3-hyroxyacyl-CoA | Metabolic process |
| 97 | WP_004418510.1 | Cytoplasmic | Enoyl-CoA hydratase/isomerase | Hydration of 2-trans-enoyl-CoA to 3-hyroxyacyl-CoA | Metabolic process |
| 98 | WP_057405861.1 | Cytoplasmic | Enoyl-CoA hydratase/isomerase | Hydration of 2-trans-enoyl-CoA to 3-hyroxyacyl-CoA | Metabolic process |
| 99 | WP_024649315.1 | Cytoplasmic | FAD dependent oxidoreductase | Catalyses oxidation-reduction processes in various metabolic pathways | Metabolic process |
| 100 | WP_044313154.1 | Cytoplasmic | FAD dependent oxidoreductase | Catalyses oxidation-reduction processes in various metabolic pathways | Metabolic process |
| 101 | WP_003371105.1 | Cytoplasmic | Riboflavin kinase domain | Biosynthesis of riboflavin | Metabolic process |
| 102 | WP_003405430.1 | Cytoplasmic | Carbohydrate kinase, FGGY | ATP dependent phosphorylation of different sugar substrates in carbohydrate metabolic process | Metabolic process |
| 103 | WP_003341445.1 | Cytoplasmic | Fructose-1-6-bisphosphatase | Conversion of fructose 1,6-bis-phosphate to fructose 6-phosphate by removal of 1 phosphate | Metabolic process |
| 104 | WP_003316351.1 | Cytoplasmic | Fructose-bisphosphate aldolase class-II | Reversible aldol condensation and cleavage of fructose-1,6-bisphosphate into glyceraldehyde 3-phosphate and dihyroxyacetone phosphate | Metabolic process |
| 105 | WP_016567230.1 | Cytoplasmic | GDP-mannose 4,6 dehydratase | Catalyses the conversion of GDP mannose to GDP-4-dehydro-6-deoxy-D-mannose | Metabolic process |
| 106 | WP_032658950.1 | Cytoplasmic | GDP-mannose 4,6 dehydratase | Catalyses the conversion of GDP mannose to GDP-4-dehydro-6-deoxy-D-mannose | Metabolic process |
| 107 | WP_057406615.1 | Cytoplasmic | GDP-mannose 4,6 dehydratase | Catalyses the conversion of GDP mannose to GDP-4-dehydro-6-deoxy-D-mannose | Metabolic process |
| 108 | WP_003341087.1 | Cytoplasmic | Glutamine amidotransferase class-I | Formation of new carbon nitrogen group by removing and transferring ammonia group from glutamine to other substrate | Metabolic process |
| 109 | WP_003315085.1 | Cytoplasmic | Glyceraldehyde 3-phosphate dehydrogenase | Interconversion of glyceraldehyde 3-phosphate and 1,3-diphosphoglycerate during gluconeogenesis and glycolysis | Metabolic process |
| 110 | WP_024649853.1 | Cytoplasmic | HAD-hyrolase-like | Involve in cleavage of carbon-halogen bonds by nucleophilic substitution reactions and have hydolase activity also | Metabolic process |
| 111 | WP_044313003.1 | Cytoplasmic | Haloacid dehalogenase-like hydrolase | Involve in cleavage of carbon-halogen bonds by nucleophilic substitution reactions and have hydolase activity also | Metabolic process |
| 112 | WP_024683957.1 | Cytoplasmic | Histidinol dehydrogenase | Catalyses terminal step in histidine biosynthesis | Metabolic process |
| 113 | WP_003316306.1 | Cytoplasmic | HIT domain | Present in nucleotide hydrolase and transferase which have catalytic activity on alpha-phosphate of ribonucleotides | Metabolic process |
| 114 | WP_003421933.1 | Cytoplasmic | 2-isopropylmalate synthase LeuA, allosteric (dimerisation) domain | Catalyses first step in leucine biosynthetic pathway | Metabolic process |
| 115 | WP_044312960.1 | Cytoplasmic | 2-isopropylmalate synthase LeuA, allosteric (dimerisation) domain | Catalyses first step in leucine biosynthetic pathway | Metabolic process |
| 116 | WP_046719726.1 | Cytoplasmic | Hydroxyacylglutathione hydrolase C-terminus | Catalyses last step in methylgyoxal conversion to lactic acid and reduced glutathione | Metabolic process |
| 117 | WP_010424090.1 | Cytoplasmic | Iron-containing alcohol dehydrogenase | Reversible oxidation of ethanol to acetaldehyde and reduction of NAD | Metabolic process |
| 118 | WP_057407211.1 | Cytoplasmic | Iron-containing alcohol dehydrogenase | Reversible oxidation of ethanol to acetaldehyde and reduction of NAD | Metabolic process |
| 119 | WP_016568419.1 | Cytoplasmic | Isochorismatase | Have hydrolytic activity to carry out various metabolic processes | Metabolic process |
| 120 | WP_024649723.1 | Cytoplasmic | Isochorismatase | Have hydrolytic activity to carry out various metabolic processes | Metabolic process |
| 121 | WP_046266756.1 | Cytoplasmic | Isochorismatase | Have hydrolytic activity to carry out various metabolic processes | Metabolic process |
| 122 | WP_016567277.1 | Periplasmic | Isochorismatase, RutB | Have hydrolytic activity to carry out various metabolic processes | Metabolic process |
| 123 | WP_004418320.1 | Cytoplasmic | Isocitrate/isopropylmalate dehydrogenase | Decarboxylation of isocitrate into alpha-ketoglutarate and Leucine biosynthesis | Metabolic process |
| 124 | WP_057405512.1 | Cytoplasmic | Malic enzyme, NAD binding | Oxidative decarboxylation of malate to pyruvate | Metabolic process |
| 125 | WP_003316648.1 | Cytoplasmic | Malic enzyme, NAD-binding | Oxidative decarboxylation of malate to pyruvate | Metabolic process |
| 126 | WP_003424754.1 | Cytoplasmic | Metal-dependent hydrolase | Hydrolytic cleavage of wide spectrum of substrates | Metabolic process |
| 127 | WP_024648389.1 | Cytoplasmic | Metal-dependent hydrolase | Hydrolytic cleavage of wide spectrum of substrates | Metabolic process |
| 128 | WP_057405846.1 | Cytoplasmic | Metal-dependent hydrolase | Hydrolytic cleavage of wide spectrum of substrates | Metabolic process |
| 129 | WP_057406751.1 | Cytoplasmic | Metal-dependent hydrolase | Hydrolytic cleavage of wide spectrum of substrates | Metabolic process |
| 130 | WP_057407099.1 | Cytoplasmic | Metal-dependent hydrolase | Hydrolytic cleavage of wide spectrum of substrates | Metabolic process |
| 131 | WP_044313570.1 | Cytoplasmic | N-acetylmuramoyl-L-alanine amidase | Cleavage of amide bond between N-acetymuramoyl and L-amino acids in peptidoglycan cellwall of bacteria | Metabolic process |
| 132 | WP_003396908.1 | Cytoplasmic | NAD dependent epimerase/dehydratase family | Catalytic activity on nucleotide-sugar substrates using NAD as cofactor | Metabolic process |
| 133 | WP_003428798.1 | InnerMembrane | CbiA superfamily | Cobalamin biosynthesis from uroporphyrinogen | Metabolic process |
| 134 | WP_003317959.1 | Cytoplasmic | NUDIX domain | Hydrolytic cleavage of broad range of pyrophosphates and play roles cellular metabolism and homeostasis | Metabolic process |
| 135 | WP_003372295.1 | Cytoplasmic | NUDIX domain | Hydrolytic cleavage of broad range of pyrophosphates and play roles cellular metabolism and homeostasis | Metabolic process |
| 136 | WP_004403846.1 | Cytoplasmic | NUDIX domain | Hydrolytic cleavage of broad range of pyrophosphates and play roles cellular metabolism and homeostasis | Metabolic process |
| 137 | WP_010411436.1 | Cytoplasmic | NUDIX domain | Hydrolytic cleavage of broad range of pyrophosphates and play roles cellular metabolism and homeostasis | Metabolic process |
| 138 | WP_057406359.1 | Cytoplasmic | NUDIX domain | Hydrolytic cleavage of broad range of pyrophosphates and play roles cellular metabolism and homeostasis | Metabolic process |
| 139 | WP_057406587.1 | Cytoplasmic | NUDIX domain | Hydrolytic cleavage of broad range of pyrophosphates and play roles cellular metabolism and homeostasis | Metabolic process |
| 140 | WP_057406959.1 | Periplasmic | PA14 domain | Have binding function rather than catalytic and are more compatible with carbohydrate binding | Metabolic process |
| 141 | WP_057407151.1 | Periplasmic | PA14 domain | Have binding function rather than catalytic and are more compatible with carbohydrate binding | Metabolic process |
| 142 | WP_057407145.1 | Cytoplasmic | Pantoate-beta-alanine ligase | Formation of pantothenate from pantoate and alanine during biosnthesis pathway of pantothenate | Metabolic process |
| 143 | WP_003425778.1 | Cytoplasmic | Peptidase family M20/M25/M40 | Release of amino acid (neutral or hydrophobic) from N-terminal of polypeptide | Metabolic process |
| 144 | WP_016568387.1 | Cytoplasmic | Peptidase family M20/M25/M40 | Release of amino acid (neutral or hydrophobic) from N-terminal of polypeptide | Metabolic process |
| 145 | WP_029573202.1 | Cytoplasmic | Peptidase family M20/M25/M40 | Release of amino acid (neutral or hydrophobic) from N-terminal of polypeptide | Metabolic process |
| 146 | WP_057405722.1 | Cytoplasmic | Peptidase family M20/M25/M40 | Release of amino acid (neutral or hydrophobic) from N-terminal of polypeptide | Metabolic process |
| 147 | WP_057405878.1 | Cytoplasmic | Peptidase family M20/M25/M40 | Release of amino acid (neutral or hydrophobic) from N-terminal of polypeptide | Metabolic process |
| 148 | WP_057406533.1 | Cytoplasmic | Peptidase family M20/M25/M40 | Release of amino acid (neutral or hydrophobic) from N-terminal of polypeptide | Metabolic process |
| 149 | WP_057406523.1 | Cytoplasmic | Phosphoglucomutase/phosphomannomutase, alpha/beta/alpha domain I | Catalyses intramolecular transferase and phosphotransferase activity on their sugar substrates | Metabolic process |
| 150 | WP_058877209.1 | Cytoplasmic | Phosphoglucomutase/phosphomannomutase, alpha/beta/alpha domain I | Catalyses intramolecular transferase and phosphotransferase activity on their sugar substrates | Metabolic process |
| 151 | WP_003391630.1 | Cytoplasmic | Methylthioadenosine phosphorylase | Reversible phosphorylation of S-methyl-5'-thioadenosine to 5-methythioribose-1 phosphate and adenine | Metabolic process |
| 152 | WP_057406216.1 | Cytoplasmic | Phosphotriesterase family | Catabolic activity on synthetic organophosphate triesters and phosphofluoridates | Metabolic process |
| 153 | WP_002554892.1 | Cytoplasmic | Polysaccharide deacetylase | Hydrolysis of O-linked acetyl groups or N-linked acetyl groups from O-acetylxylose or GlcNAc respectively | Metabolic process |
| 154 | WP_003343034.1 | Cytoplasmic | Polysaccharide deacetylase | Hydrolysis of O-linked acetyl groups or N-linked acetyl groups from O-acetylxylose or GlcNAc respectively | Metabolic process |
| 155 | WP_003421167.1 | Cytoplasmic | Riboflavin biosynthesis protein RibD | Bifunctional protein catalyses second and third of riboflavin biosynthesis by oxidation-reduction process | Metabolic process |
| 156 | WP_002554639.1 | Cytoplasmic | Ribonucleotide reductase, small chain | Reductive synthesis of deoxyribonucleotides from ribonucleotides which are used as precursors for DNA synthesis | Metabolic process |
| 157 | WP_057405982.1 | Cytoplasmic | Shikimate dehydrogenase substrate binding domain | Biosynthesis of precursors for aromatic amino acids | Metabolic process |
| 158 | WP_003314121.1 | Cytoplasmic | Short-chain dehydrogenase/reductase SDR | Oxidoreductases which are critical for amino acid, carbohydrate, lipid and xenobiotic metabolism | Metabolic process |
| 159 | WP_003370854.1 | Cytoplasmic | Short-chain dehydrogenase/reductase SDR | Oxidoreductases which are critical for amino acid, carbohydrate, lipid and xenobiotic metabolism | Metabolic process |
| 160 | WP_003408650.1 | Cytoplasmic | Short-chain dehydrogenase/reductase SDR | Oxidoreductases which are critical for amino acid, carbohydrate, lipid and xenobiotic metabolism | Metabolic process |
| 161 | WP_003409893.1 | Cytoplasmic | Short-chain dehydrogenase/reductase SDR | Oxidoreductases which are critical for amino acid, carbohydrate, lipid and xenobiotic metabolism | Metabolic process |
| 162 | WP_003412971.1 | Cytoplasmic | Short-chain dehydrogenase/reductase SDR | Oxidoreductases which are critical for amino acid, carbohydrate, lipid and xenobiotic metabolism | Metabolic process |
| 163 | WP_003418561.1 | Cytoplasmic | Short-chain dehydrogenase/reductase SDR | Oxidoreductases which are critical for amino acid, carbohydrate, lipid and xenobiotic metabolism | Metabolic process |
| 164 | WP_024692722.1 | Cytoplasmic | Short-chain dehydrogenase/reductase SDR | Oxidoreductases which are critical for amino acid, carbohydrate, lipid and xenobiotic metabolism | Metabolic process |
| 165 | WP_024693568.1 | Cytoplasmic | Short-chain dehydrogenase/reductase SDR | Oxidoreductases which are critical for amino acid, carbohydrate, lipid and xenobiotic metabolism | Metabolic process |
| 166 | WP_032661249.1 | Cytoplasmic | Short-chain dehydrogenase/reductase SDR | Oxidoreductases which are critical for amino acid, carbohydrate, lipid and xenobiotic metabolism | Metabolic process |
| 167 | WP_044311717.1 | Cytoplasmic | Short-chain dehydrogenase/reductase SDR | Oxidoreductases which are critical for amino acid, carbohydrate, lipid and xenobiotic metabolism | Metabolic process |
| 168 | WP_044312262.1 | Cytoplasmic | Short-chain dehydrogenase/reductase SDR | Oxidoreductases which are critical for amino acid, carbohydrate, lipid and xenobiotic metabolism | Metabolic process |
| 169 | WP_044312332.1 | Cytoplasmic | Short-chain dehydrogenase/reductase SDR | Oxidoreductases which are critical for amino acid, carbohydrate, lipid and xenobiotic metabolism | Metabolic process |
| 170 | WP_044313635.1 | Cytoplasmic | Short-chain dehydrogenase/reductase SDR | Oxidoreductases which are critical for amino acid, carbohydrate, lipid and xenobiotic metabolism | Metabolic process |
| 171 | WP_057406481.1 | Cytoplasmic | Short-chain dehydrogenase/reductase SDR | Oxidoreductases which are critical for amino acid, carbohydrate, lipid and xenobiotic metabolism | Metabolic process |
| 172 | WP_057407207.1 | Cytoplasmic | Short-chain dehydrogenase/reductase SDR | Oxidoreductases which are critical for amino acid, carbohydrate, lipid and xenobiotic metabolism | Metabolic process |
| 173 | WP_057407565.1 | Cytoplasmic | Short-chain dehydrogenase/reductase SDR | Oxidoreductases which are critical for amino acid, carbohydrate, lipid and xenobiotic metabolism | Metabolic process |
| 174 | WP_057407614.1 | Cytoplasmic | Short-chain dehydrogenase/reductase SDR | Oxidoreductases which are critical for amino acid, carbohydrate, lipid and xenobiotic metabolism | Metabolic process |
| 175 | WP_024663968.1 | Periplasmic | Alkaline-phosphatase-like, core domain | Hydrolysis of phosphoesters bonds at high pH | Metabolic process |
| 176 | WP_057406460.1 | Cytoplasmic | ThiF family | Biosynthesis of cysteine and methionine | Metabolic process |
| 177 | WP_003316157.1 | Cytoplasmic | Alcohol dehydrogenase GroES-like domain | Catalyses oxidation reduction reactions during fermentation its GroES fold help in maintaining structural integrity which promote productive folding | Metabolic process |
| 178 | WP_003318011.1 | Cytoplasmic | Alcohol dehydrogenase GroES-like domain | Catalyses oxidation reduction reactions during fermentation its GroES fold help in maintaining structural integrity which promote productive folding | Metabolic process |
| 179 | WP_003339560.1 | Cytoplasmic | Alcohol dehydrogenase GroES-like domain | Catalyses oxidation reduction reactions during fermentation its GroES fold help in maintaining structural integrity which promote productive folding | Metabolic process |
| 180 | WP_003365244.1 | Cytoplasmic | Alcohol dehydrogenase GroES-like domain | Catalyses oxidation reduction reactions during fermentation its GroES fold help in maintaining structural integrity which promote productive folding | Metabolic process |
| 181 | WP_003406175.1 | Cytoplasmic | Alcohol dehydrogenase GroES-like domain | Catalyses oxidation reduction reactions during fermentation its GroES fold help in maintaining structural integrity which promote productive folding | Metabolic process |
| 182 | WP_004417774.1 | Cytoplasmic | Alcohol dehydrogenase GroES-like domain | Catalyses oxidation reduction reactions during fermentation its GroES fold help in maintaining structural integrity which promote productive folding | Metabolic process |
| 183 | WP_044309986.1 | Cytoplasmic | Alcohol dehydrogenase GroES-like domain | Catalyses oxidation reduction reactions during fermentation its GroES fold help in maintaining structural integrity which promote productive folding | Metabolic process |
| 184 | WP_044313084.1 | Cytoplasmic | Alcohol dehydrogenase GroES-like domain | Catalyses oxidation reduction reactions during fermentation its GroES fold help in maintaining structural integrity which promote productive folding | Metabolic process |
| 185 | WP_057405775.1 | Cytoplasmic | Alcohol dehydrogenase GroES-like domain | Catalyses oxidation reduction reactions during fermentation its GroES fold help in maintaining structural integrity which promote productive folding | Metabolic process |
| 186 | WP_057405923.1 | Cytoplasmic | Alcohol dehydrogenase GroES-like domain | Catalyses oxidation reduction reactions during fermentation its GroES fold help in maintaining structural integrity which promote productive folding | Metabolic process |
| 187 | WP_057405981.1 | Cytoplasmic | Alcohol dehydrogenase GroES-like domain | Catalyses oxidation reduction reactions during fermentation its GroES fold help in maintaining structural integrity which promote productive folding | Metabolic process |
| 188 | WP_057406151.1 | Cytoplasmic | Alcohol dehydrogenase GroES-like domain | Catalyses oxidation reduction reactions during fermentation its GroES fold help in maintaining structural integrity which promote productive folding | Metabolic process |
| 189 | WP_057406576.1 | Cytoplasmic | Alcohol dehydrogenase GroES-like domain | Catalyses oxidation reduction reactions during fermentation its GroES fold help in maintaining structural integrity which promote productive folding | Metabolic process |
| 190 | WP_057407601.1 | Cytoplasmic | Alcohol dehydrogenase GroES-like domain | Catalyses oxidation reduction reactions during fermentation its GroES fold help in maintaining structural integrity which promote productive folding | Metabolic process |
| 191 | WP_044311531.1 | Cytoplasmic | B3/4 domain | Catalyses tRNA editing reactions | Protein biosynthesis |
| 192 | WP_003316380.1 | Cytoplasmic | Elongation factor Tu GTP binding domain | GTP dependent elongation factor in translation | Protein biosynthesis |
| 193 | WP_003317109.1 | Cytoplasmic | Elongation factor Tu GTP binding domain | GTP dependent elongation factor in translation | Protein biosynthesis |
| 194 | WP_003363707.1 | Cytoplasmic | Elongation factor Tu GTP binding domain | GTP dependent elongation factor in translation | Protein biosynthesis |
| 195 | WP_003413009.1 | Cytoplasmic | Elongation factor Tu GTP binding domain | GTP dependent elongation factor in translation | Protein biosynthesis |
| 196 | WP_003318474.1 | Cytoplasmic | Polypeptide deformylase | Formyl group removal from N-terminal of polypeptide chain | Protein biosynthesis |
| 197 | WP_057406328.1 | Cytoplasmic | Polypeptide deformylase | Formyl group removal from N-terminal of polypeptide chain | Protein biosynthesis |
| 198 | WP_002555494.1 | Cytoplasmic | Ribosomal protein S12/S23 | Structural constituents of ribosome catalyses mRNA directed protein synthesis | Protein biosynthesis |
| 199 | WP_002555476.1 | Cytoplasmic | Ribosomal protein S14p/S29e | Structural constituents of ribosome catalyses mRNA directed protein synthesis | Protein biosynthesis |
| 200 | WP_002555465.1 | Cytoplasmic | Ribosomal protein S4/S9 N-terminal domain | Structural constituents of ribosome catalyses mRNA directed protein synthesis | Protein biosynthesis |
| 201 | WP_003317099.1 | Cytoplasmic | Ribosomal protein S5, C-terminal domain | Structural constituents of ribosome catalyses mRNA directed protein synthesis | Protein biosynthesis |
| 202 | WP_003317104.1 | Cytoplasmic | Ribosomal Proteins L2, C-terminal domain | Structural constituents of ribosome catalyses mRNA directed protein synthesis | Protein biosynthesis |
| 203 | WP_057406270.1 | Cytoplasmic | Translation initiation factor aIF-2, bacterial-like | GTP dependent binding of tRNA with ribosomal small subunit during initiation of translation | Protein biosynthesis |
| 204 | WP_003315564.1 | Cytoplasmic | tRNA synthetase class II core domain (G, H, P, S and T) | Catalyses addition of amino acid to its cognate tRNA at 3' hydroxyl site | Protein biosynthesis |
| 205 | WP_003315286.1 | Cytoplasmic | tRNA synthetases class I (C) catalytic domain | Catalyses addition of amino acid to its cognate tRNA at 2' hydroxyl site | Protein biosynthesis |
| 206 | WP_024664904.1 | Cytoplasmic | tRNA synthetases class I (E and Q), catalytic domain | Catalyses addition of amino acid to its cognate tRNA at 2' hydroxyl site | Protein biosynthesis |
| 207 | WP_029572663.1 | Cytoplasmic | tRNA synthetases class I (E and Q), catalytic domain | Catalyses addition of amino acid to its cognate tRNA at 2' hydroxyl site | Protein biosynthesis |
| 208 | WP_044313577.1 | Cytoplasmic | tRNA synthetases class I (E and Q), catalytic domain | Catalyses addition of amino acid to its cognate tRNA at 2' hydroxyl site | Protein biosynthesis |
| 209 | WP_057406060.1 | Cytoplasmic | tRNA synthetases class I (I, L, M and V) | Catalyses addition of amino acid to its cognate tRNA at 2' hydroxyl site | Protein biosynthesis |
| 210 | WP_057406288.1 | Cytoplasmic | tRNA synthetases class I (I, L, M and V) | Catalyses addition of amino acid to its cognate tRNA at 2' hydroxyl site | Protein biosynthesis |
| 211 | WP_057405821.1 | Cytoplasmic | tRNA synthetases class I (M) | Catalyses addition of amino acid to its cognate tRNA at 2' hydroxyl site | Protein biosynthesis |
| 212 | WP_057407217.1 | Cytoplasmic | tRNA synthetases class I (M) | Catalyses addition of amino acid to its cognate tRNA at 2' hydroxyl site | Protein biosynthesis |
| 213 | WP_057405911.1 | cytoplasmic | tRNA synthetases class II (A) | Catalyses addition of amino acid to its cognate tRNA at 3' hydroxyl site | Protein biosynthesis |
| 214 | WP_003390678.1 | Cytoplasmic | Cyclophilin type peptidyl-prolyl cis-trans isomerase/CLD | Catalyses cis-trans isomerization of proline imidic peptide binds in oligopeptides to speed up the rate of protein folding | Protein folding |
| 215 | WP_003395180.1 | Cytoplasmic | DnaJ C terminal domain | Regulate ATPase activity of Hsp70 and prevent the aggregation of unfolded polypeptide chains | Protein folding |
| 216 | WP_003313641.1 | Cytoplasmic | Hsp70 protein | Bind to partially exposed hydrophobic surfaces cause aggregation and protein folding, prevent other non productive interactions | Protein folding |
| 217 | WP_004416350.1 | Cytoplasmic | Hsp70 protein | Bind to partially exposed hydrophobic surfaces cause aggregation and protein folding, prevent other non productive interactions | Protein folding |
| 218 | WP_004418787.1 | Cytoplasmic | Hsp70 protein | Bind to partially exposed hydrophobic surfaces cause aggregation and protein folding, prevent other non productive interactions | Protein folding |
| 219 | WP_003318786.1 | Cytoplasmic | Peptidyl-prolyl cis-trans isomerase domain | Catalyses cis-trans isomerization of proline imidic peptide binds in oligopeptides to speed up the rate of protein folding | Protein folding |
| 220 | WP_003340445.1 | Cytoplasmic | Peptidyl-prolyl cis-trans isomerase domain | Catalyses cis-trans isomerization of proline imidic peptide binds in oligopeptides to speed up the rate of protein folding | Protein folding |
| 221 | WP_003349508.1 | Cytoplasmic | Trx-like domain/YbbN | Promote assembly of proteins involved in and stress response and DNA replication | Protein folding |
| 222 | WP_003317954.1 | Cytoplasmic | AAA domain | ATPase which are concerned with variety of cellular activities | Proteolysis |
| 223 | WP_057406993.1 | Cytoplasmic | Peptidase M16, zinc-binding site | Metalloendopeptidase activity to degrade small peptides | Proteolysis |
| 224 | WP_003348756.1 | Cytoplasmic | Peptidase C56, PfpI | Have deglycase activity to repair methylgyoxal and glyoxal glycated proteins and nucleic acids | Proteolysis |
| 225 | WP_057406994.1 | Cytoplasmic | Peptidase S9, prolyl oligopeptidase, catalytic domain | Have Serine-type peptidase activity that cleaves peptide bonds at propyl residues of C terminal | Proteolysis |
| 226 | WP_057406961.1 | Extracellular | Thermolysin metallopeptidase, alpha-helical domain/Peptidase M4 | Secreted thermostable metalloprotease used to hydrolyze peptide | Proteolysis |
| 227 | WP_044312611.1 | Periplasmic | Trypsin | Cleavage of polypeptide chain mainly at carboxyl terminal of lysine or arginine | Proteolysis |
| 228 | WP_003391089.1 | Cytoplasmic | Metallopeptidase family M24 | Removal of methionine (initiator amino terminal) from prokaryotic cytoplasmic proteins if penultimate amino acid is uncharged and small | Proteolysis |
| 229 | WP_003416218.1 | Cytoplasmic | Metallopeptidase family M24 | Removal of methionine (initiator amino terminal) from prokaryotic cytoplasmic proteins if penultimate amino acid is uncharged and small | Proteolysis |
| 230 | WP_003426021.1 | Cytoplasmic | Metallopeptidase family M24 | Removal of methionine (initiator amino terminal) from prokaryotic cytoplasmic proteins if penultimate amino acid is uncharged and small | Proteolysis |
| 231 | WP_044313467.1 | Cytoplasmic | Peptidase family M3 | Metalloendopeptidase activity to degrade medium size proteins | Proteolysis |
| 232 | WP_003313633.1 | InnerMembrane | Peptidase family M41 | ATP dependent metalloprotease for the correct assembly of the proteins | Proteolysis |
| 233 | WP_003411605.1 | Extracellular | Peptidase M10 serralysin | Extracellular endopeptidases which have ability to degrade plant-cellwall | Proteolysis |
| 234 | WP_057406970.1 | Extracellular | Peptidase M10 serralysin | Extracellular endopeptidases which have ability to degrade plant-cellwall | Proteolysis |
| 235 | WP_003368366.1 | Cytoplasmic | Peptidase M18 | Aminopeptidase activity to release amino acid of N-terminal | Proteolysis |
| 236 | WP_057406416.1 | Cytoplasmic | Peptidase_M17 | Aminopeptidase activity to cleave leucine residues from N terminal of polypeptide | Proteolysis |
| 237 | WP_003316346.1 | InnerMembrane | Peptidase_M19 | Membrane dipeptidase to hydrolyze dipeptides | Proteolysis |
| 238 | WP_057406027.1 | InnerMembrane | Peptidase_M19 | Membrane dipeptidase to hydrolyze dipeptides | Proteolysis |
| 239 | WP_057407566.1 | Periplasmic | Azurin family | Act as redox stress sensor and protect the cell from radical brust | Response to oxidative stress |
| 240 | WP_002554851.1 | Cytoplasmic | Bacterioferritin/Ferritin-like diiron domain | Have ferroxidase activity which assist detoxification and protection from radical species | Response to oxidative stress |
| 241 | WP_003317095.1 | Cytoplasmic | Bacterioferritin/Ferritin-like diiron domain | Have ferroxidase activity which assist detoxification and protection from radical species | Response to oxidative stress |
| 242 | WP_057406103.1 | Cytoplasmic | Bacterioferritin/Ferritin-like diiron domain | Have ferroxidase activity which assist detoxification and protection from radical species | Response to oxidative stress |
| 243 | WP_057406401.1 | Periplasmic | Copper/zinc superoxide dismutase (SODC) | Catalyses the dismutation of superoxide radicals to hydrogen peroxide or molecular oxygen (O_2_ ) | Response to oxidative stress |
| 244 | WP_057407465.1 | Periplasmic | DJ-1/PfpI family | Have amniopeptidase, endopeptidase and glyoxalase activity which functions in protection of cells from oxidative stress | Response to oxidative stress |
| 245 | WP_003314356.1 | Cytoplasmic | Ferritin/DPS protein domain | Have ferroxidase activity which assist detoxification and protection from radical species | Response to oxidative stress |
| 246 | WP_057405779.1 | Cytoplasmic | Heavy-metal-associated domain | Bacterial resistance to toxic metals | Response to oxidative stress |
| 247 | WP_004403157.1 | Periplasmic | Multicopper oxidase | Catalyses oxidation reduction reactions and are involved copper tolerance | Response to oxidative stress |
| 248 | WP_003315309.1 | Cytoplasmic | Pyridine nucleotide-disulphide oxidoreductase | Formation of dislphide by redox active cysteines | Response to oxidative stress |
| 249 | WP_003341167.1 | Cytoplasmic | Rhodanese-like domain | Sulphotransferase activity for the detoxification of cyanide | Response to oxidative stress |
| 250 | WP_003317268.1 | Cytoplasmic | D-Tyr-tRNA(Tyr) deacylase | tRNA editing and cleavage of D-aminoacyl-tRNA into d-amino acid and free tRNA | RNA processing |
| 251 | WP_032660892.1 | Cytoplasmic | Exonuclease, RNase T | Maturation and degradation of RNA molecules | RNA processing |
| 252 | WP_057407574.1 | Cytoplasmic | Exonuclease, RNase T | Maturation and degradation of RNA molecules | RNA processing |
| 253 | WP_003411784.1 | Cytoplasmic | tRNA N6-adenosine threonylcarbamoyltransferase, TsaD | Have metalloendopeptidase activity to catalyse tRNA modification and cell-wall peptidoglycan modification | RNA processing |
| 254 | WP_003421956.1 | Cytoplasmic | Cytidine and deoxycytidylate deaminases, zinc-binding | Hydrolysis of cytidine to uridine and ammonia by cytidine deaminase and hydrolysis of dCMP to dUMP by deoxycytidylate deaminase | RNA processing |
| 255 | WP_016569379.1 | Cytoplasmic | MnmE helical domain | Multidomain GTPase involved in tRNA modification by forming a complex with MnmG | RNA processing |
| 256 | WP_003340592.1 | Cytoplasmic | PNPase/RNase PH domain | Maturation and degradation of RNA molecules | RNA processing |
| 257 | WP_057406374.1 | Cytoplasmic | Queuine tRNA-ribosyltransferase | Catalyses the base exchange reaction to insert queuine into tRNA instead of guanine | RNA processing |
| 258 | WP_057407569.1 | Cytoplasmic | RNB domain | Maturation and degradation of RNA molecules | RNA processing |
| 259 | WP_004416157.1 | Cytoplasmic | tRNA pseudouridine synthase II, TruB | Involved in RNA modification and pseudouridine synthesis by nucleotide flipping tRNA tertiary structure distruption | RNA processing |
| 260 | WP_057406727.1 | Cytoplasmic | Zn-dependent metallo-hydrolase, RNA specificity domain | Have endonuclease activity for mRNA processing | RNA processing |
| 261 | WP_024663382.1 | Cytoplasmic | Cold-shock' DNA-binding domain | DNA and RNA binding proteins involve in regulation of transcription during sudden decrease in growth temperature | Transcription regulation |
| 262 | WP_003313643.1 | Cytoplasmic | Ferric uptake regulator family | DNA binding metal uptake transcription regulator to maintain metal ion intracellular concentration in bacteria | Transcription regulation |
| 263 | WP_057406041.1 | Cytoplasmic | Histone deacetylase domain | Remove the acetyl group of histones and contribute to regulate transcription repression | Transcription regulation |
| 264 | WP_003393431.1 | Cytoplasmic | MerR-type HTH domain | DNA binding transcription regulators which responds to external stimuli such as metal, antibiotics and oxidative stress | Transcription regulation |
| 265 | WP_003413458.1 | Cytoplasmic | Sigma-54 interaction domain | ATP dependent transcription regulatory proteins to activate the promoters gene expression for translation initiation | Transcription regulation |
| 266 | WP_003415893.1 | Cytoplasmic | SNF2 family N-terminal domain | ATP binding protein used to disrupt histone-DNA interactions for raising accessibility of DNA to transcription factors | Transcription regulation |
| 267 | WP_003414024.1 | Cytoplasmic | Transcription regulator HTH, GntR | DNA binding transcription regulatory protein | Transcription regulation |
| 268 | WP_057406983.1 | Cytoplasmic | Transcription regulator HTH, GntR | DNA binding transcription regulatory protein | Transcription regulation |
| 269 | WP_003316106.1 | Cytoplasmic | Rubredoxin | Redox active electron carrier protein mainly in anaerobes | Transport |
| 270 | WP_003314434.1 | InnerMembrane | ABC transporter | Integral membrane ATP-binding cassette transporters which uses ATP for import and export of wide range of substrates including metal ions | Transport |
| 271 | WP_003317094.1 | InnerMembrane | ABC transporter | Integral membrane ATP-binding cassette transporters which uses ATP for import and export of wide range of substrates including metal ions | Transport |
| 272 | WP_003317226.1 | InnerMembrane | ABC transporter | Integral membrane ATP-binding cassette transporters which uses ATP for import and export of wide range of substrates including metal ions | Transport |
| 273 | WP_003363626.1 | InnerMembrane | ABC transporter | Integral membrane ATP-binding cassette transporters which uses ATP for import and export of wide range of substrates including metal ions | Transport |
| 274 | WP_003405691.1 | InnerMembrane | ABC transporter | Integral membrane ATP-binding cassette transporters which uses ATP for import and export of wide range of substrates including metal ions | Transport |
| 275 | WP_003412889.1 | InnerMembrane | ABC transporter | Integral membrane ATP-binding cassette transporters which uses ATP for import and export of wide range of substrates including metal ions | Transport |
| 276 | WP_004416899.1 | InnerMembrane | ABC transporter | Integral membrane ATP-binding cassette transporters which uses ATP for import and export of wide range of substrates including metal ions | Transport |
| 277 | WP_044312717.1 | InnerMembrane | ABC transporter | Integral membrane ATP-binding cassette transporters which uses ATP for import and export of wide range of substrates including metal ions | Transport |
| 278 | WP_057405772.1 | InnerMembrane | ABC transporter | Integral membrane ATP-binding cassette transporters which uses ATP for import and export of wide range of substrates including metal ions | Transport |
| 279 | WP_057405964.1 | InnerMembrane | ABC transporter | Integral membrane ATP-binding cassette transporters which uses ATP for import and export of wide range of substrates including metal ions | Transport |
| 280 | WP_057406409.1 | InnerMembrane | ABC transporter | Integral membrane ATP-binding cassette transporters which uses ATP for import and export of wide range of substrates including metal ions | Transport |
| 281 | WP_057406916.1 | InnerMembrane | ABC transporter | Integral membrane ATP-binding cassette transporters which uses ATP for import and export of wide range of substrates including metal ions | Transport |
| 282 | WP_057406928.1 | InnerMembrane | ABC transporter | Integral membrane ATP-binding cassette transporters which uses ATP for import and export of wide range of substrates including metal ions | Transport |
| 283 | WP_057407128.1 | InnerMembrane | ABC transporter | Integral membrane ATP-binding cassette transporters which uses ATP for import and export of wide range of substrates including metal ions | Transport |
| 284 | WP_058877178.1 | InnerMembrane | ABC transporter | Integral membrane ATP-binding cassette transporters which uses ATP for import and export of wide range of substrates including metal ions | Transport |
| 285 | WP_003317349.1 | Periplasmic | Bacterial extracellular solute-binding proteins, family 3 | Act as receptors for transport and signal transduction system | Transport |
| 286 | WP_003424807.1 | Periplasmic | Bacterial extracellular solute-binding proteins, family 3 | Act as receptors for transport and signal transduction system | Transport |
| 287 | WP_003428827.1 | Periplasmic | Bacterial extracellular solute-binding proteins, family 3 | Act as receptors for transport and signal transduction system | Transport |
| 288 | WP_044313164.1 | Periplasmic | Bacterial extracellular solute-binding proteins, family 3 | Act as receptors for transport and signal transduction system | Transport |
| 289 | WP_057406454.1 | Periplasmic | Bacterial extracellular solute-binding proteins, family 3 | Act as receptors for transport and signal transduction system | Transport |
| 290 | WP_057406536.1 | Periplasmic | Bacterial extracellular solute-binding proteins, family 3 | Act as receptors for transport and signal transduction system | Transport |
| 291 | WP_003318111.1 | Periplasmic | Bacterial extracellular solute-binding proteins, family 5 | Act as receptors for transport and signal transduction system | Transport |
| 292 | WP_057405715.1 | Periplasmic | Bacterial extracellular solute-binding proteins, family 5 | Act as receptors for transport and signal transduction system | Transport |
| 293 | WP_057406194.1 | Periplasmic | Bacterial extracellular solute-binding proteins, family 5 | Act as receptors for transport and signal transduction system | Transport |
| 294 | WP_057406522.1 | Periplasmic | Bacterial extracellular solute-binding proteins, family 5 | Act as receptors for transport and signal transduction system | Transport |
| 295 | WP_057406766.1 | Periplasmic | Bacterial extracellular solute-binding proteins, family 5 | Act as receptors for transport and signal transduction system | Transport |
| 296 | WP_057406898.1 | Periplasmic | Bacterial extracellular solute-binding proteins, family 5 | Act as receptors for transport and signal transduction system | Transport |
| 297 | WP_057406924.1 | Periplasmic | Bacterial extracellular solute-binding proteins, family 5 | Act as receptors for transport and signal transduction system | Transport |
| 298 | WP_057407520.1 | Periplasmic | Bacterial extracellular solute-binding proteins, family 5 | Act as receptors for transport and signal transduction system | Transport |
| 299 | WP_058877211.1 | Periplasmic | Bacterial extracellular solute-binding proteins, family 5 | Act as receptors for transport and signal transduction system | Transport |
| 300 | WP_003341876.1 | InnerMembrane | Cytochrome C and Quinol oxidase polypeptide I | Catalyses the last step in electron transport chain i.e reduction of oxygen to water after obtaining the electrons from cytochrome c and transfer them to oxygen | Transport |
| 301 | WP_003421830.1 | InnerMembrane | Cytochrome c oxidase subunit III | Catalyses the last step in electron transport chain i.e reduction of oxygen to water after obtaining the electrons from cytochrome c and transfer them to oxygen | Transport |
| 302 | WP_057405863.1 | Periplasmic | Cytochrome c, class IA/ IB | Catalyses the last step in electron transport chain i.e reduction of oxygen to water after obtaining the electrons from cytochrome c and transfer them to oxygen | Transport |
| 303 | WP_057405780.1 | InnerMembrane | P-type ATPase | ATP dependent transport of metal ions and other compounds across the membrane | Transport |
| 304 | WP_003346032.1 | Periplasmic | Periplasmic binding protein domain | Act as receptors for transport and signal transduction system | Transport |
| 305 | WP_003425596.1 | Periplasmic | Periplasmic binding protein domain | Act as receptors for transport and signal transduction system | Transport |
| 306 | WP_010408289.1 | Cytoplasmic | Pilus retraction protein PilT | ATPase couped proteins required for surface-associated translocation i.e twitching and gliding motility | Transport |
| 307 | WP_057407594.1 | Cytoplasmic | Pilus retraction protein PilT | ATPase couped proteins required for surface-associated translocation i.e twitching and gliding motility | Transport |

**Supplementary Table S6 : The details of functionally enriched GO network of zinc binding proteins**

| **S. No.** | **GO ID** | **GO Term** | **GO Groups** | **Nr. Genes** | **Degree** |
| --- | --- | --- | --- | --- | --- |
| 1 | GO:0043170 | macromolecule metabolic process | Group11, Group16, Group17 | 75 | 91 |
| 2 | GO:0034641 | cellular nitrogen compound metabolic process | Group11, Group17 | 71 | 90 |
| 3 | GO:0044271 | cellular nitrogen compound biosynthetic process | Group11, Group16, Group17 | 46 | 84 |
| 4 | GO:0044260 | cellular macromolecule metabolic process | Group11, Group16, Group17 | 62 | 82 |
| 5 | GO:0044249 | cellular biosynthetic process | Group11, Group16, Group17 | 59 | 80 |
| 6 | GO:0034645 | cellular macromolecule biosynthetic process | Group11, Group16, Group17 | 41 | 78 |
| 7 | GO:0009059 | macromolecule biosynthetic process | Group11, Group16, Group17 | 41 | 76 |
| 8 | GO:1901576 | organic substance biosynthetic process | Group11, Group16, Group17 | 59 | 76 |
| 9 | GO:1901360 | organic cyclic compound metabolic process | Group11, Group17 | 59 | 76 |
| 10 | GO:0010467 | gene expression | Group11, Group16, Group17 | 39 | 75 |
| 11 | GO:0006139 | nucleobase-containing compound metabolic process | Group11, Group17 | 54 | 74 |
| 12 | GO:0046483 | heterocycle metabolic process | Group11, Group17 | 58 | 74 |
| 13 | GO:0006725 | cellular aromatic compound metabolic process | Group11, Group17 | 57 | 73 |
| 14 | GO:0016070 | RNA metabolic process | Group11, Group16, Group17 | 31 | 68 |
| 15 | GO:1901564 | organonitrogen compound metabolic process | Group16 | 57 | 68 |
| 16 | GO:0090304 | nucleic acid metabolic process | Group11, Group17 | 45 | 61 |
| 17 | GO:1901566 | organonitrogen compound biosynthetic process | Group16, Group17 | 37 | 58 |
| 18 | GO:0019538 | protein metabolic process | Group11, Group16 | 41 | 57 |
| 19 | GO:0018130 | heterocycle biosynthetic process | Group17 | 25 | 54 |
| 20 | GO:1901362 | organic cyclic compound biosynthetic process | Group17 | 26 | 54 |
| 21 | GO:0019438 | aromatic compound biosynthetic process | Group17 | 24 | 53 |
| 22 | GO:0044267 | cellular protein metabolic process | Group11, Group16, Group17 | 28 | 52 |
| 23 | GO:0034654 | nucleobase-containing compound biosynthetic process | Group11, Group17 | 21 | 52 |
| 24 | GO:0006412 | translation | Group16, Group17 | 21 | 48 |
| 25 | GO:0050794 | regulation of cellular process | Group17 | 24 | 48 |
| 26 | GO:0043603 | cellular amide metabolic process | Group16, Group17 | 22 | 47 |
| 27 | GO:0043604 | amide biosynthetic process | Group16, Group17 | 22 | 46 |
| 28 | GO:0019222 | regulation of metabolic process | Group17 | 18 | 46 |
| 29 | GO:0060255 | regulation of macromolecule metabolic process | Group17 | 18 | 46 |
| 30 | GO:0019752 | carboxylic acid metabolic process | Group15, Group16 | 24 | 45 |
| 31 | GO:0043043 | peptide biosynthetic process | Group16, Group17 | 21 | 45 |
| 32 | GO:0031323 | regulation of cellular metabolic process | Group17 | 17 | 45 |
| 33 | GO:0006518 | peptide metabolic process | Group16, Group17 | 21 | 44 |
| 34 | GO:0010468 | regulation of gene expression | Group17 | 18 | 44 |
| 35 | GO:0010556 | regulation of macromolecule biosynthetic process | Group17 | 17 | 44 |
| 36 | GO:0031326 | regulation of cellular biosynthetic process | Group17 | 17 | 44 |
| 37 | GO:2000112 | regulation of cellular macromolecule biosynthetic process | Group17 | 17 | 44 |
| 38 | GO:0006082 | organic acid metabolic process | Group15, Group16 | 24 | 43 |
| 39 | GO:0043436 | oxoacid metabolic process | Group15, Group16 | 24 | 43 |
| 40 | GO:0009889 | regulation of biosynthetic process | Group17 | 17 | 43 |
| 41 | GO:0051171 | regulation of nitrogen compound metabolic process | Group17 | 17 | 43 |
| 42 | GO:0080090 | regulation of primary metabolic process | Group17 | 17 | 42 |
| 43 | GO:0006520 | cellular amino acid metabolic process | Group16 | 17 | 40 |
| 44 | GO:0032774 | RNA biosynthetic process | Group17 | 14 | 39 |
| 45 | GO:0006399 | tRNA metabolic process | Group07, Group16, Group17 | 14 | 38 |
| 46 | GO:0034660 | ncRNA metabolic process | Group07, Group16, Group17 | 14 | 38 |
| 47 | GO:0034220 | ion transmembrane transport | Group09, Group10, Group14 | 6 | 36 |
| 48 | GO:0098660 | inorganic ion transmembrane transport | Group09, Group10, Group14 | 6 | 35 |
| 49 | GO:0007165 | signal transduction | Group17 | 15 | 35 |
| 50 | GO:0035556 | intracellular signal transduction | Group17 | 15 | 35 |
| 51 | GO:0000160 | phosphorelay signal transduction system | Group17 | 15 | 34 |
| 52 | GO:0016053 | organic acid biosynthetic process | Group15, Group16 | 10 | 29 |
| 53 | GO:0006811 | ion transport | Group09, Group10, Group12, Group14 | 13 | 28 |
| 54 | GO:0046394 | carboxylic acid biosynthetic process | Group15, Group16 | 10 | 28 |
| 55 | GO:0043039 | tRNA aminoacylation | Group16, Group17 | 10 | 28 |
| 56 | GO:0006820 | anion transport | Group09, Group14 | 6 | 27 |
| 57 | GO:0044283 | small molecule biosynthetic process | Group15, Group16 | 12 | 27 |
| 58 | GO:0043038 | amino acid activation | Group16, Group17 | 10 | 27 |
| 59 | GO:0006259 | DNA metabolic process | Group03, Group05 | 14 | 26 |
| 60 | GO:0055086 | nucleobase-containing small molecule metabolic process | Group08, Group13 | 8 | 26 |
| 61 | GO:0046942 | carboxylic acid transport | Group09, Group14 | 5 | 26 |
| 62 | GO:0006732 | coenzyme metabolic process | Group13, Group15 | 6 | 26 |
| 63 | GO:0006865 | amino acid transport | Group09, Group14 | 5 | 25 |
| 64 | GO:0071705 | nitrogen compound transport | Group09, Group14 | 7 | 25 |
| 65 | GO:0006418 | tRNA aminoacylation for protein translation | Group16 | 9 | 25 |
| 66 | GO:0043412 | macromolecule modification | Group04, Group07 | 13 | 24 |
| 67 | GO:0006753 | nucleoside phosphate metabolic process | Group08, Group13 | 5 | 24 |
| 68 | GO:0015711 | organic anion transport | Group09, Group14 | 5 | 24 |
| 69 | GO:0015849 | organic acid transport | Group09, Group14 | 5 | 24 |
| 70 | GO:0009108 | coenzyme biosynthetic process | Group15 | 5 | 24 |
| 71 | GO:0051188 | cofactor biosynthetic process | Group15 | 6 | 24 |
| 72 | GO:0015698 | inorganic anion transport | Group09, Group14 | 4 | 23 |
| 73 | GO:0098656 | anion transmembrane transport | Group14 | 3 | 23 |
| 74 | GO:0098661 | inorganic anion transmembrane transport | Group14 | 3 | 23 |
| 75 | GO:1905039 | carboxylic acid transmembrane transport | Group14 | 3 | 23 |
| 76 | GO:0009117 | nucleotide metabolic process | Group08, Group13 | 5 | 22 |
| 77 | GO:0030001 | metal ion transport | Group09, Group10, Group12 | 5 | 22 |
| 78 | GO:0050801 | ion homeostasis | Group12 | 3 | 22 |
| 79 | GO:0055065 | metal ion homeostasis | Group12 | 3 | 22 |
| 80 | GO:0099131 | ATP hydrolysis coupled ion transmembrane transport | Group14 | 3 | 22 |
| 81 | GO:1903825 | organic acid transmembrane transport | Group14 | 3 | 22 |
| 82 | GO:0032787 | monocarboxylic acid metabolic process | Group15 | 6 | 22 |
| 83 | GO:1901575 | organic substance catabolic process | Group02, Group08, Group13 | 10 | 21 |
| 84 | GO:0006873 | cellular ion homeostasis | Group12 | 3 | 21 |
| 85 | GO:0006875 | cellular metal ion homeostasis | Group12 | 3 | 21 |
| 86 | GO:0019725 | cellular homeostasis | Group12 | 5 | 21 |
| 87 | GO:0030003 | cellular cation homeostasis | Group12 | 3 | 21 |
| 88 | GO:0042592 | homeostatic process | Group12 | 5 | 21 |
| 89 | GO:0046916 | cellular transition metal ion homeostasis | Group12 | 3 | 21 |
| 90 | GO:0048878 | chemical homeostasis | Group12 | 3 | 21 |
| 91 | GO:0055080 | cation homeostasis | Group12 | 3 | 21 |
| 92 | GO:0055082 | cellular chemical homeostasis | Group12 | 3 | 21 |
| 93 | GO:0072521 | purine-containing compound metabolic process | Group13 | 4 | 21 |
| 94 | GO:0003333 | amino acid transmembrane transport | Group14 | 3 | 21 |
| 95 | GO:0090662 | ATP hydrolysis coupled transmembrane transport | Group14 | 3 | 21 |
| 96 | GO:0099133 | ATP hydrolysis coupled anion transmembrane transport | Group14 | 3 | 21 |
| 97 | GO:0000041 | transition metal ion transport | Group10, Group12 | 3 | 20 |
| 98 | GO:0055076 | transition metal ion homeostasis | Group12 | 3 | 20 |
| 99 | GO:0098771 | inorganic ion homeostasis | Group12 | 3 | 20 |
| 100 | GO:1901135 | carbohydrate derivative metabolic process | Group13 | 11 | 20 |
| 101 | GO:0006812 | cation transport | Group09, Group10, Group12, Group14 | 7 | 19 |
| 102 | GO:0055085 | transmembrane transport | Group09, Group14 | 13 | 19 |
| 103 | GO:0006879 | cellular iron ion homeostasis | Group12 | 3 | 19 |
| 104 | GO:0019637 | organophosphate metabolic process | Group08, Group13 | 7 | 18 |
| 105 | GO:0006417 | regulation of translation | Group06 | 4 | 17 |
| 106 | GO:0044248 | cellular catabolic process | Group08 | 8 | 17 |
| 107 | GO:0072522 | purine-containing compound biosynthetic process | Group13 | 3 | 17 |
| 108 | GO:0051186 | cofactor metabolic process | Group15 | 7 | 17 |
| 109 | GO:0072330 | monocarboxylic acid biosynthetic process | Group15 | 3 | 17 |
| 110 | GO:0006508 | proteolysis | Group16 | 15 | 17 |
| 111 | GO:0051246 | regulation of protein metabolic process | Group06 | 4 | 16 |
| 112 | GO:0006396 | RNA processing | Group07, Group16 | 6 | 16 |
| 113 | GO:0009123 | nucleoside monophosphate metabolic process | Group13, Group15 | 3 | 16 |
| 114 | GO:1901137 | carbohydrate derivative biosynthetic process | Group13 | 5 | 16 |
| 115 | GO:0008652 | cellular amino acid biosynthetic process | Group15, Group16 | 6 | 16 |
| 116 | GO:0006464 | cellular protein modification process | Group04, Group07 | 7 | 15 |
| 117 | GO:0036211 | protein modification process | Group04, Group07 | 7 | 15 |
| 118 | GO:0006414 | translational elongation | Group06 | 5 | 15 |
| 119 | GO:0032268 | regulation of cellular protein metabolic process | Group06 | 4 | 15 |
| 120 | GO:0034248 | regulation of cellular amide metabolic process | Group06 | 4 | 15 |
| 121 | GO:0008033 | tRNA processing | Group07, Group16 | 5 | 15 |
| 122 | GO:0009451 | RNA modification | Group07, Group16 | 5 | 15 |
| 123 | GO:0034470 | ncRNA processing | Group07, Group16 | 5 | 15 |
| 124 | GO:0046700 | heterocycle catabolic process | Group08, Group13 | 4 | 15 |
| 125 | GO:1901361 | organic cyclic compound catabolic process | Group08, Group13 | 4 | 15 |
| 126 | GO:0098655 | cation transmembrane transport | Group10, Group14 | 3 | 15 |
| 127 | GO:0009161 | ribonucleoside monophosphate metabolic process | Group13, Group15 | 3 | 15 |
| 128 | GO:0009259 | ribonucleotide metabolic process | Group13, Group15 | 3 | 15 |
| 129 | GO:0006400 | tRNA modification | Group07, Group16 | 5 | 14 |
| 130 | GO:0044270 | cellular nitrogen compound catabolic process | Group08, Group13 | 5 | 14 |
| 131 | GO:0098662 | inorganic cation transmembrane transport | Group10, Group14 | 3 | 14 |
| 132 | GO:0016310 | phosphorylation | Group13 | 8 | 14 |
| 133 | GO:1901605 | alpha-amino acid metabolic process | Group15, Group16 | 6 | 14 |
| 134 | GO:0006631 | fatty acid metabolic process | Group15 | 3 | 14 |
| 135 | GO:0044272 | sulfur compound biosynthetic process | Group15 | 3 | 14 |
| 136 | GO:0006448 | regulation of translational elongation | Group06 | 3 | 13 |
| 137 | GO:0010608 | posttranscriptional regulation of gene expression | Group06 | 4 | 13 |
| 138 | GO:0015672 | monovalent inorganic cation transport | Group10, Group14 | 3 | 13 |
| 139 | GO:0015980 | energy derivation by oxidation of organic compounds | Group10, Group14 | 3 | 13 |
| 140 | GO:0045333 | cellular respiration | Group10, Group14 | 3 | 13 |
| 141 | GO:0043101 | purine-containing compound salvage | Group13 | 3 | 13 |
| 142 | GO:1901607 | alpha-amino acid biosynthetic process | Group15, Group16 | 5 | 13 |
| 143 | GO:0006790 | sulfur compound metabolic process | Group15 | 3 | 13 |
| 144 | GO:0009110 | vitamin biosynthetic process | Group15 | 3 | 13 |
| 145 | GO:0044255 | cellular lipid metabolic process | Group15 | 4 | 13 |
| 146 | GO:0019439 | aromatic compound catabolic process | Group08, Group13 | 3 | 12 |
| 147 | GO:1901565 | organonitrogen compound catabolic process | Group08 | 5 | 12 |
| 148 | GO:0009060 | aerobic respiration | Group10, Group14 | 3 | 12 |
| 149 | GO:0006629 | lipid metabolic process | Group15 | 4 | 12 |
| 150 | GO:0018193 | peptidyl-amino acid modification | Group04, Group07 | 4 | 11 |
| 151 | GO:0006996 | organelle organization | Group05 | 6 | 11 |
| 152 | GO:0051276 | chromosome organization | Group05 | 5 | 11 |
| 153 | GO:0071103 | DNA conformation change | Group05 | 5 | 11 |
| 154 | GO:0006450 | regulation of translational fidelity | Group06 | 3 | 11 |
| 155 | GO:0006091 | generation of precursor metabolites and energy | Group10 | 4 | 11 |
| 156 | GO:0043094 | cellular metabolic compound salvage | Group13 | 3 | 11 |
| 157 | GO:0006766 | vitamin metabolic process | Group15 | 3 | 11 |
| 158 | GO:0006767 | water-soluble vitamin metabolic process | Group15 | 3 | 11 |
| 159 | GO:0042364 | water-soluble vitamin biosynthetic process | Group15 | 3 | 11 |
| 160 | GO:0005975 | carbohydrate metabolic process | Group00 | 9 | 10 |
| 161 | GO:0090305 | nucleic acid phosphodiester bond hydrolysis | Group01 | 6 | 10 |
| 162 | GO:0006265 | DNA topological change | Group05 | 4 | 10 |
| 163 | GO:0006281 | DNA repair | Group03, Group05 | 4 | 9 |
| 164 | GO:0006974 | cellular response to DNA damage stimulus | Group03, Group05 | 4 | 9 |
| 165 | GO:0018208 | peptidyl-proline modification | Group04 | 3 | 9 |
| 166 | GO:0006260 | DNA replication | Group05 | 6 | 9 |
| 167 | GO:0090501 | RNA phosphodiester bond hydrolysis | Group01 | 3 | 8 |
| 168 | GO:0033554 | cellular response to stress | Group03 | 5 | 8 |
| 169 | GO:0000413 | protein peptidyl-prolyl isomerization | Group04 | 3 | 8 |
| 170 | GO:1901615 | organic hydroxy compound metabolic process | Group02 | 4 | 7 |
| 171 | GO:0090503 | RNA phosphodiester bond hydrolysis, exonucleolytic | Group01 | 3 | 6 |
| 172 | GO:0044282 | small molecule catabolic process | Group02, Group08 | 3 | 6 |
| 173 | GO:0006066 | alcohol metabolic process | Group02 | 3 | 6 |
| 174 | GO:0016311 | dephosphorylation | Group00 | 3 | 4 |

**Supplementary Table S7: Secreted zinc binding proteins**

| **S. No.** | **Sequence Id** | **Signal Peptide** | **Tat motif** | **Position** | **Sec score** | **Transmembrane helix** |
| --- | --- | --- | --- | --- | --- | --- |
| 1 | WP_003346032.1 | Y | - | 30 | - | Nil |
| 2 | WP_003425596.1 | Y | - | 27 | - | Nil |
| 3 | WP_003317349.1 | Y | - | 26 | - | Nil |
| 4 | WP_003424807.1 | Y | - | 22 | - | Nil |
| 5 | WP_003428827.1 | Y | - | 24 | - | Nil |
| 6 | WP_044313164.1 | - | - | - | 0.92565 | Nil |
| 7 | WP_057406454.1 | Y | - | 23 | - | Nil |
| 8 | WP_057406536.1 | Y | - | 30 | - | Nil |
| 9 | WP_003318111.1 | - | - | - | 0.9107 | Nil |
| 10 | WP_057405715.1 | Y | - | 24 | - | Nil |
| 11 | WP_057406194.1 | Y | - | 23 | - | Nil |
| 12 | WP_057406522.1 | Y | - | 22 | - | Nil |
| 13 | WP_057406766.1 | Y | - | 26 | - | Nil |
| 14 | WP_057406898.1 | Y | Y | 25 (Tat motif starting at position 60) | - | Nil |
| 15 | WP_057406924.1 | - | - | - | 0.87032 | Nil |
| 16 | WP_057407520.1 | Y | - | 27 | - | Nil |
| 17 | WP_058877211.1 | Y | - | 25 | - | Nil |
| 18 | WP_057407431.1 | Y | - | 22 | - | Nil |
| 19 | WP_003316096.1 | Y | - | 21 | - | Nil |
| 20 | WP_003422785.1 | - | - | - | 0.95363 | Nil |
| 21 | WP_057405551.1 | - | - | - | 0.94151 | Nil |
| 22 | WP_057406372.1 | Y | - | 20 | - | Nil |
| 23 | WP_044312611.1 | Y | - | 20 | - | Nil |
| 24 | WP_057406961.1 | - | - | - | 0.90378 | Nil |
| 25 | WP_003411605.1 | - | - | - | 0.95431 | Nil |
| 26 | WP_057406970.1 | - | - | - | 0.9312 | Nil |
| 27 | WP_057407465.1 | - | - | - | 0.90617 | Nil |
| 28 | WP_057406401.1 | Y | - | 20 | - | Nil |
| 29 | WP_057407566.1 | Y | - | 21 | - | Nil |
| 30 | WP_003317093.1 | - | - | - | 0.91074 | Nil |
| 31 | WP_016567277.1 | - | - | - | 0.75461 | Nil |

**Supplementary Table S8: List of interacting residues in secreted zinc binding proteins**

| **S. No.** | **Sequence Id** | **Template** | **Interacting residues** |
| --- | --- | --- | --- |
| 1 | WP_003346032.1 | 1GUD | PHE32, GLU88, LYS162, GLU299 |
| 2 | WP_003425596.1 | 4ZPJ | GLU72, TYR76, ASP114, ALA160, ASP173, GLY178, ASP208, LYS217, ALA284, ALA287, GLN297 |
| 3 | WP_003317349.1 | 2YJP | ALA160, GLU162, ALA177, ALA181 |
| 4 | WP_003424807.1 | 2Y7I | LYS27, ALA41, ASP43, ASP73, ALA85, GLY161 |
| 5 | WP_003428827.1 | 2YJP | ASN170, ASP172, GLN190, SER194 |
| 6 | WP_044313164.1 | 2YJP | HIS160, ASP162, GLN176, ASN180 |
| 7 | WP_057406454.1 | 2Y7I | ASN50, LYS52, ASP82, ASP94, ASP182 |
| 8 | WP_057406536.1 | 2Y7I | ASP51, PRO82, ASP94 |
| 9 | WP_003318111.1 | 1UQW | ASP216, LYS229, ASP275, ARG297, ASP370 |
| 10 | WP_057405715.1 | 1UQW | THR191, GLY240, VAL262, ASP340, TYR484 |
| 11 | WP_057406194.1 | 1UQW | VAL186, ASP231, ASN253, ASP341 |
| 12 | WP_057406522.1 | 1XOC | ASP 51, GLU149, ALA153, THR159, ASP223, ASP242, THR323, GLU325, GLY375, THR 459, ALA502 |
| 13 | WP_057406766.1 | 1XOC | ASP83, TYR158, ILE166, PRO170, LYS176, HIS198, LYS236, ASP262, ASN361, GLU363, ASP425, MET428, ASP430, GLU448, ARG450, ALA525 |
| 14 | WP_057406898.1 | 1XOC | GLN84, GLN160, GLU168, PRO172, ASP178, HIS200, PRO239, ASP265, ASN364, GLU366, LYS431, ASP429, LEU431, ASN433, ALA528, SER571 |
| 15 | WP_057406924.1 | 1UQW | GLN132, SER187, GLU205, GLN258, THR518, ASP356 |
| 16 | WP_057407520.1 | 1XOC | GLU86, TYR159, THR170, PRO174, ASP267, PHE356, GLU360, LYS432, LEU433, ASN 435, ALA534 |
| 17 | WP_058877211.1 | 1UQW | LYS213, GLU259, LYS281, ASP361 |
| 18 | WP_057407431.1 | 4WBG | ASN43, ARG149, HIS167, MET180, LYS226, TYR279 |
| 19 | WP_003316096.1 | 2HSI | ASP182, HIS265, HIS267 |
| 20 | WP_003422785.1 | 2GU1 | HIS337, ASP341, HIS420 |
| 21 | WP_057405551.1 | 2GU1 | ASP192, HIS269 |
| 22 | WP_057406372.1 | 2HSI | HIS172, ASP176, HIS251, HIS253 |
| 23 | WP_044312611.1 | 4D9Q | GLU149 |
| 24 | WP_057406961.1 | 2VQX | HIS176, HIS180, GLU200 |
| 25 | WP_003411605.1 | 1OMJ | HIS182, HIS186, HIS192 |
| 26 | WP_057406970.1 | 1H71 | HIS154, HIS158, HIS164 |
| 27 | WP_057407465.1 | 1ONS | LYS35, ASP40, ALA74 |
| 28 | WP_057406401.1 | 1EQW | HIS92, HIS101, HIS109, ASP112 |
| 29 | WP_057407566.1 | 1NWP | ASP82, HIS103 |
| 30 | WP_003317093.1 | 1UE1 | LYS62, GLU65 |
| 31 | WP_016567277.1 | 1EEF | LYS62, GLU65 |

**Supplementary Table S9 : Subcellular localization and Functional domain analysis of copper binding proteins**

| **S. No.** | **Sequence ID** | **Subcellular localization** | **Functional domain/family** | **Description** | **Broad Class** |
| --- | --- | --- | --- | --- | --- |
| 1 | WP_003345939.1 | Cytoplasmic | Carbonic anhydrase | Reversible hydration of carbon dioxide | Metabolic process |
| 2 | WP_004409041.1 | Cytoplasmic | Carbonic anhydrase | Reversible hydration of carbon dioxide | Metabolic process |
| 3 | WP_032659812.1 | Cytoplasmic | Glutathione S-transferase, N-terminal domain | Catalyses nucleophilic transfer of tripeptide glutathione to the substrate having reactive eletrophilic functional groups and are involved in biodegradative metabolism of xenobiotic compounds | Metabolic process |
| 4 | WP_057406698.1 | Cytoplasmic | Glutathione S-transferase, N-terminal domain | Catalyses nucleophilic transfer of tripeptide glutathione to the substrate having reactive eletrophilic functional groups and are involved in biodegradative metabolism of xenobiotic compounds | Metabolic process |
| 5 | WP_046719726.1 | Cytoplasmic | Hydroxyacylglutathione hydrolase C-terminus | Catalyses last step in methylgyoxal conversion to lactic acid and reduced glutathione | Metabolic process |
| 6 | WP_057407099.1 | Cytoplasmic | Metal-dependent hydrolase | Hydrolytic cleavage of wide spectrum of substrates | Metabolic process |
| 7 | WP_057405848.1 | Cytoplasmic | Aldehyde oxidase/xanthine dehydrogenase, molybdopterin binding | Involve in oxidation-reduction reactions to catalyze various metabolic processes | Metabolic process |
| 8 | WP_057407039.1 | Cytoplasmic | Aldehyde oxidase/xanthine dehydrogenase, molybdopterin binding | Involve in oxidation-reduction reactions to catalyze various metabolic processes | Metabolic process |
| 9 | WP_003316157.1 | Cytoplasmic | Alcohol dehydrogenase GroES-like domain | Catalyses oxidation reduction reactions during fermentation its GroES fold help in maintaining structural integrity which promote productive folding | Metabolic process |
| 10 | WP_003318011.1 | Cytoplasmic | Alcohol dehydrogenase GroES-like domain | Catalyses oxidation reduction reactions during fermentation its GroES fold help in maintaining structural integrity which promote productive folding | Metabolic process |
| 11 | WP_003339560.1 | Cytoplasmic | Alcohol dehydrogenase GroES-like domain | Catalyses oxidation reduction reactions during fermentation its GroES fold help in maintaining structural integrity which promote productive folding | Metabolic process |
| 12 | WP_003365244.1 | Cytoplasmic | Alcohol dehydrogenase GroES-like domain | Catalyses oxidation reduction reactions during fermentation its GroES fold help in maintaining structural integrity which promote productive folding | Metabolic process |
| 13 | WP_003406175.1 | Cytoplasmic | Alcohol dehydrogenase GroES-like domain | Catalyses oxidation reduction reactions during fermentation its GroES fold help in maintaining structural integrity which promote productive folding | Metabolic process |
| 14 | WP_004417774.1 | Cytoplasmic | Alcohol dehydrogenase GroES-like domain | Catalyses oxidation reduction reactions during fermentation its GroES fold help in maintaining structural integrity which promote productive folding | Metabolic process |
| 15 | WP_057406151.1 | Cytoplasmic | Alcohol dehydrogenase GroES-like domain | Catalyses oxidation reduction reactions during fermentation its GroES fold help in maintaining structural integrity which promote productive folding | Metabolic process |
| 16 | WP_057406556.1 | Cytoplasmic | Alcohol dehydrogenase GroES-like domain | Catalyses oxidation reduction reactions during fermentation its GroES fold help in maintaining structural integrity which promote productive folding | Metabolic process |
| 17 | WP_057406576.1 | Cytoplasmic | Alcohol dehydrogenase GroES-like domain | Catalyses oxidation reduction reactions during fermentation its GroES fold help in maintaining structural integrity which promote productive folding | Metabolic process |
| 18 | WP_057407601.1 | Cytoplasmic | Alcohol dehydrogenase GroES-like domain | Catalyses oxidation reduction reactions during fermentation its GroES fold help in maintaining structural integrity which promote productive folding | Metabolic process |
| 19 | WP_003424002.1 | Periplasmic | Copper chaperone SCO1/SenC | Required for the assembly of cytochrome c oxidase subunits and protection of bacteria against stress | Protein folding |
| 20 | WP_057407280.1 | Periplasmic | Copper chaperone SCO1/SenC | Required for the assembly of cytochrome c oxidase subunits and protection of bacteria against stress | Protein folding |
| 21 | WP_003347894.1 | Cytoplasmic | UreE urease accessory protein, C-terminal domain | Required for the biogenesis and assembly of urease metallocentre | Protein folding |
| 22 | WP_003349508.1 | Cytoplasmic | Trx-like domain/YbbN | Promote assembly of proteins involved in and stress response and DNA replication | Protein folding |
| 23 | WP_044312661.1 | Cytoplasmic | Trx-like domain/YbbN | Promote assembly of proteins involved in and stress response and DNA replication | Protein folding |
| 24 | WP_057406106.1 | Cytoplasmic | Trx-like domain/YbbN | Promote assembly of proteins involved in and stress response and DNA replication | Protein folding |
| 25 | WP_044312611.1 | Periplasmic | Trypsin | Cleavage of polypeptide chain mainly at carboxyl terminal of lysine or arginine | Proteolysis |
| 26 | WP_004416738.1 | Periplasmic | AhpC/TSA family | Catalyses oxidation-reduction reactions and have antioxidant activity to maintain cell redox homeostasis | Response to oxidative stress |
| 27 | WP_057407566.1 | Periplasmic | Azurin family | Act as redox stress sensor and protect the cell from radical brust | Response to oxidative stress |
| 28 | WP_057406401.1 | Periplasmic | Copper/zinc superoxide dismutase (SODC) | Catalyses the dismutation of superoxide radicals to hydrogen peroxide or molecular oxygen (O_2_ ) | Response to oxidative stress |
| 29 | WP_057405779.1 | Cytoplasmic | Heavy-metal-associated domain | Bacterial resistance to toxic metals | Response to oxidative stress |
| 30 | WP_004403157.1 | Periplasmic | Multicopper oxidase | Catalyses oxidation reduction reactions and are involved copper tolerance | Response to oxidative stress |
| 31 | WP_057405503.1 | Periplasmic | Multicopper oxidase | Catalyses oxidation reduction reactions and are involved copper tolerance | Response to oxidative stress |
| 32 | WP_010434512.1 | Cytoplasmic | MerR HTH family regulatory protein | DNA binding transcription regulators which responds to external stimuli such as metal, antibiotics and oxidative stress | Transcription regulation |
| 33 | WP_003393431.1 | Cytoplasmic | MerR, DNA binding | DNA binding transcription regulators which responds to external stimuli such as metal, antibiotics and oxidative stress | Transcription regulation |
| 34 | WP_057406514.1 | Periplasmic | Bacterial extracellular solute-binding protein | Act as receptors for transport and signal transduction system | Transport |
| 35 | WP_003341876.1 | InnerMembrane | Cytochrome C and Quinol oxidase polypeptide I | Catalyses the last step in electron transport chain i.e reduction of oxygen to water after obtaining the electrons from cytochrome c and transfer them to oxygen | Transport |
| 36 | WP_003421829.1 | InnerMembrane | Cytochrome C oxidase subunit II, transmembrane domain | Catalyses the last step in electron transport chain i.e reduction of oxygen to water after obtaining the electrons from cytochrome c and transfer them to oxygen | Transport |
| 37 | WP_057405780.1 | InnerMembrane | P-type ATPase | ATP dependent transport of metal ions and other compounds across the membrane | Transport |
| 38 | WP_057407383.1 | InnerMembrane | P-type ATPase | ATP dependent transport of metal ions and other compounds across the membrane | Transport |

**Supplementary Table S10: The details of functionally enriched GO network of copper binding proteins**

| **S. No.** | **GOID** | **GOTerm** | **GOGroups** | **Nr. Genes** | **Degree** |
| --- | --- | --- | --- | --- | --- |
| 1 | GO:0009893 | positive regulation of metabolic process | Group3 | 2 | 21 |
| 2 | GO:0010604 | positive regulation of macromolecule metabolic process | Group3 | 2 | 20 |
| 3 | GO:0031325 | positive regulation of cellular metabolic process | Group3 | 2 | 20 |
| 4 | GO:1902680 | positive regulation of RNA biosynthetic process | Group3 | 2 | 20 |
| 5 | GO:0009891 | positive regulation of biosynthetic process | Group3 | 2 | 19 |
| 6 | GO:0010557 | positive regulation of macromolecule biosynthetic process | Group3 | 2 | 19 |
| 7 | GO:0031328 | positive regulation of cellular biosynthetic process | Group3 | 2 | 19 |
| 8 | GO:0045935 | positive regulation of nucleobase-containing compound metabolic process | Group3 | 2 | 19 |
| 9 | GO:0051254 | positive regulation of RNA metabolic process | Group3 | 2 | 19 |
| 10 | GO:0010628 | positive regulation of gene expression | Group3 | 2 | 18 |
| 11 | GO:0045893 | positive regulation of transcription, DNA-templated | Group3 | 2 | 18 |
| 12 | GO:0048518 | positive regulation of biological process | Group3 | 2 | 18 |
| 13 | GO:0048522 | positive regulation of cellular process | Group3 | 2 | 18 |
| 14 | GO:0051173 | positive regulation of nitrogen compound metabolic process | Group3 | 2 | 18 |
| 15 | GO:1903508 | positive regulation of nucleic acid-templated transcription | Group3 | 2 | 18 |
| 16 | GO:0015992 | proton transport | Group2 | 2 | 11 |
| 17 | GO:0019725 | cellular homeostasis | Group1 | 4 | 10 |
| 18 | GO:0098662 | inorganic cation transmembrane transport | Group2 | 2 | 10 |
| 19 | GO:1902600 | hydrogen ion transmembrane transport | Group2 | 2 | 10 |
| 20 | GO:0042592 | homeostatic process | Group1 | 4 | 9 |
| 21 | GO:0045454 | cell redox homeostasis | Group1 | 4 | 9 |
| 22 | GO:0006818 | hydrogen transport | Group2 | 2 | 9 |
| 23 | GO:0015672 | monovalent inorganic cation transport | Group2 | 2 | 9 |
| 24 | GO:0098655 | cation transmembrane transport | Group2 | 2 | 9 |
| 25 | GO:0006091 | generation of precursor metabolites and energy | Group2 | 2 | 8 |
| 26 | GO:0098754 | detoxification | Group0 | 2 | 7 |
| 27 | GO:1990748 | cellular detoxification | Group0 | 2 | 7 |
| 28 | GO:0006662 | glycerol ether metabolic process | Group1 | 2 | 7 |
| 29 | GO:0018904 | ether metabolic process | Group1 | 2 | 7 |
| 30 | GO:0009636 | response to toxic substance | Group0 | 2 | 6 |
| 31 | GO:0098869 | cellular oxidant detoxification | Group0 | 2 | 6 |

**Supplementary Table S11: Secreted copper binding proteins**

| **S. No.** | **Sequence Id** | **Signal Peptide** | **Tat motif** | **Cleavage site** | **Sec score** | **Transmembrane helix** |
| --- | --- | --- | --- | --- | --- | --- |
| 1 | WP_057406401.1 | Y | - | 20 | - | Nil |
| 2 | WP_057407566.1 | Y | - | 21 | - | Nil |
| 3 | WP_057406514.1 | Y | - | 23 | - | Nil |
| 4 | WP_044312611.1 | Y | - | 20 | - | Nil |
| 5 | WP_003424002.1 | - | Y | Tat motif starting at position 7 | - | Nil |

**Supplementary Table S12: List of interacting residues in secreted copper binding proteins**

| **S. No.** | **Sequence Id** | **Template** | **Interacting residues** |
| --- | --- | --- | --- |
| 1 | WP_057407566.1 | 1NWP | HIS66, CYS132, HIS137, MET141 |
| 2 | WP_057406401.1 | 1EQW | HIS67, HIS69, HIS147 |
| 3 | WP_044312611.1 | 1AND | HIS64 |
| 4 | WP_057406514.1 | 3TYH | TYR220, TYR221 |
| 5 | WP_003424002.1 | 4WBR | CYS77, CYS81, HIS164 |
